# Cell-derived lactate triggers local hydrogelation and phenotype remodelling

**DOI:** 10.1038/s41467-026-77702-x

**Published:** 2026-09-28

**Authors:** Marius G. Braun, Tom C. Schneider, Kaloian Koynov, Sophie Gieß, Leon Driehaus-Ortiz, Lisa Förch, Maximilian Schuler, Manfred Wagner, Christopher V. Synatschke, Anne Régnier-Vigouroux, Laura De Laporte, David Y. W. Ng, Tanja Weil

**Affiliations:** 1 Max Planck Institute for Polymer Research, Mainz, Germany; 2 DWI-Leibniz Institute for Interactive Materials, Aachen, Germany; 3 Institute of Technical and Macromolecular Chemistry (ITMC), Chair of Macromolecular Materials for Medicine, RWTH Aachen University, Aachen, Germany; 4 Institute of Applied Medical Engineering (AME), Department of Advanced Materials for Biomedicine (AMB), University Hospital RWTH Aachen, Center for Biohybrid Medical Systems (CMBS), Aachen, Germany; 5 Institute for Developmental Biology and Neurobiology, Johannes Gutenberg University, Mainz, Germany; 6 Max Planck School Matter to Life, Heidelberg, Germany

**Keywords:** Biomaterials, Cancer microenvironment, Gels and hydrogels, Biopolymers

## Abstract

Many tumours release lactate as a major metabolic product and signalling molecule. Here we use lactate flux as an endogenous trigger for covalent hydrogel formation. Phenol-functionalised alginate remains soluble until cell-derived lactate is oxidised by lactate oxidase; the generated hydrogen peroxide is used by horseradish peroxidase to crosslink phenols and form a matrix at metabolically active sites. Hydrogels form within minutes and reach storage moduli from approximately 10 Pa to 5 kPa. In tumour-spheroid models, including a patient-derived glioblastoma spheroid model, local lactate production generates spatially confined gelation that extended up to around 1 mm from the spheroid surface. Nanoindentation and fluorescence correlation spectroscopy reveal radial stiffness and polymer-density gradients, while proteome profiling and imaging indicate reduced proliferative signalling and increased markers associated with migration and matrix remodelling. These findings establish metabolite-gated hydrogelation as a strategy to couple cellular metabolism to covalent matrix assembly, mechanics and transport, providing a bio-synthetic platform for modelling cell–matrix reciprocity and tumour confinement.

## Abstract (web summary)

Synthetic hydrogels are widely used to mimic key extracellular matrix functions, but their biochemical and mechanical properties are typically predefined, requiring cells to adapt to a fixed environment. Here, the authors introduce a phenol-functionalized alginate hydrogel that undergoes in situ crosslinking in response to lactate secreted by cells. By coupling material formation to cellular metabolism, this system generates local mechanical gradients around metabolically active cells and spheroids, enabling dynamic, self-regulated matrix remodelling driven by metabolic fluxes.

## Introduction

The extracellular matrix (ECM) orchestrates cellular behaviour by integrating structural, biochemical, and mechanical cues^1^. While synthetic hydrogels emulate key ECM functions and are foundational to three-dimensional culture models^2^, most synthetic systems communicate with cells in a single direction; their chemistry and mechanics are preset, and cells must adapt to these imposed conditions. What remains missing is adaptive reciprocity, where materials sense endogenous changes in cellular activity and reconfigure their own state accordingly^3^.

Fluctuations in cellular metabolism provide a direct and quantitative readout of cell state^4^. In tumours, elevated glycolytic flux drives lactate accumulation, acidification, and lactate-dependent signalling that can reprogramme immunity and extracellular remodelling^5^. Compared with exogenous triggers such as pH, temperature, or light^6–8^, cell-derived metabolites provide an intrinsic, spatiotemporally-resolved handle to regulate covalent crosslinking reactions in adaptive matrix materials. In such systems, metabolites are not only biochemical readouts, but can also act as local chemical fuels that couple cell state to material formation. This insight frames our concept: cellular metabolism sets crosslinking kinetics; crosslinking defines matrix mechanics and transport; and mechanical states feed back to modulate cell phenotype^9–12^.

**Fig. 1:**
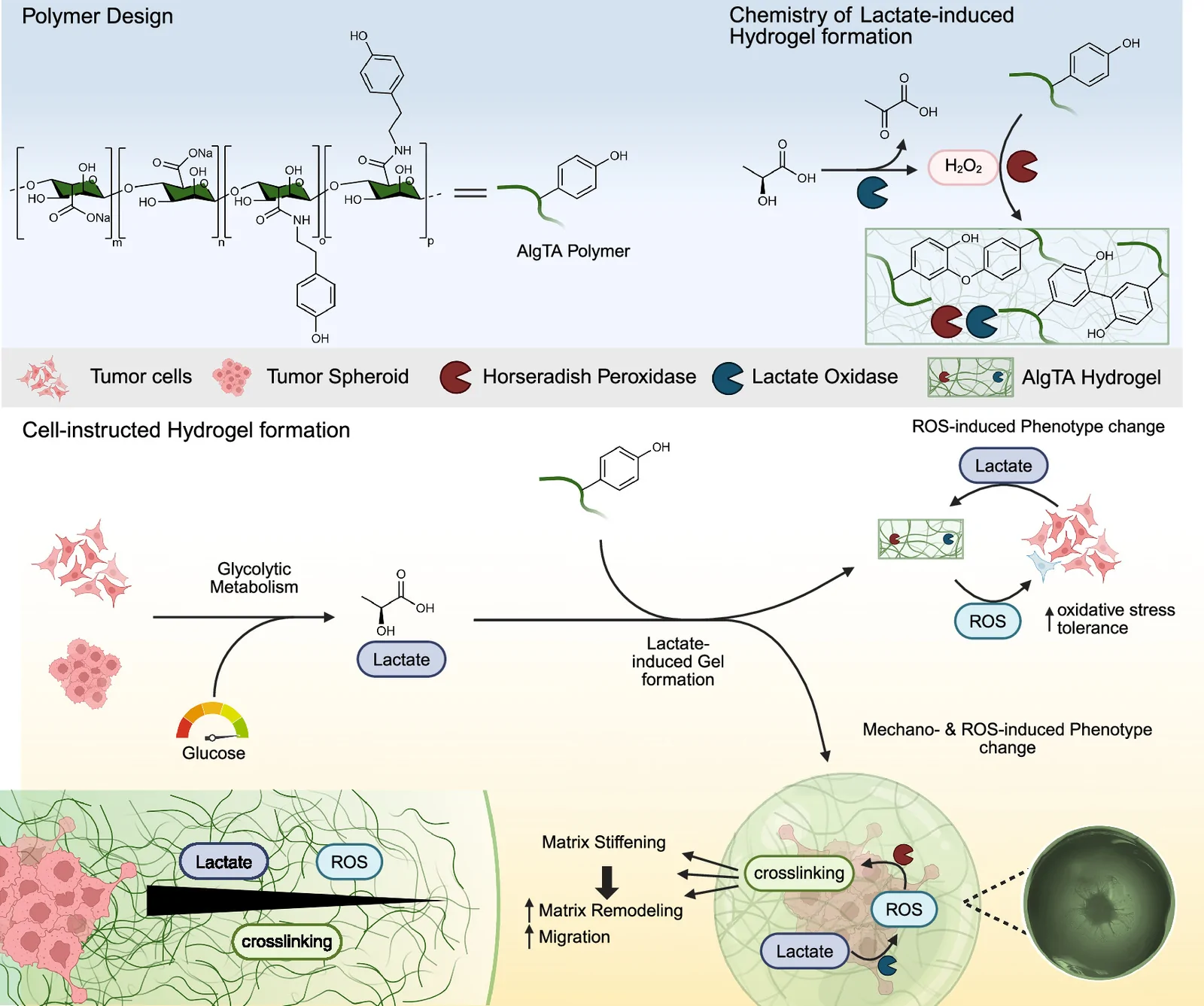
Metabolite-gated covalent crosslinking enables cell-directed gelation of phenol-functionalised alginate (AlgTA). Cell-secreted lactate is oxidised by lactate oxidase to generate hydrogen peroxide, which horseradish peroxidase uses to oxidatively couple pendant phenols into covalent crosslinks. Local lactate gradients drive spatially patterned hydrogelation and stiffness gradients around two-dimensional cultures and encapsulated three-dimensional tumour spheroids. The resulting matrix locally couples redox chemistry, transport and mechanics, generating feedback cues that alter cellular signalling, proteomic profiles and phenotype. Created in BioRender. Braun, M (2026) https://BioRender.com/569s2eu.

Here, we introduce a mode of cell-material communication that utilises a phenol-functionalised alginate, which crosslinks in situ in response to lactate secreted by cells (Fig. 1). Embedding lactate oxidase (LOx) and horseradish peroxidase (HRP) establishes a cascade that converts lactate to hydrogen peroxide, which oxidatively couples pendant phenols to form covalent network junctions in both two- and three-dimensional cultures. Because hydrogen peroxide is generated from lactate and consumed during phenol coupling, the cascade links redox flux to local matrix formation rather than relying on uniform external peroxide addition. Unlike HRP/H_2_O_2_-crosslinkable hydrogels that require exogenous peroxide^13^ or glucose-oxidase fuelling^14^, gelation here is gated by endogenous lactate, connecting material formation directly to cellular metabolism and building mechanical gradients around metabolically active cells and spheroids. These gradients in turn trigger cellular adaptation, with phenotypic changes at the proteome and morphological levels that are consistent with reduced proliferative signalling and increased migration- and matrix-remodelling-associated programmes. In contrast to enzyme-instructed supramolecular assemblies^15^, the resulting network is covalent, mechanically tuneable, and stable, yet remains cell-instructed through metabolic flux.

## Results

### Phenol-functionalised alginate supports peroxide-triggered covalent hydrogelation

**Fig. 2:**
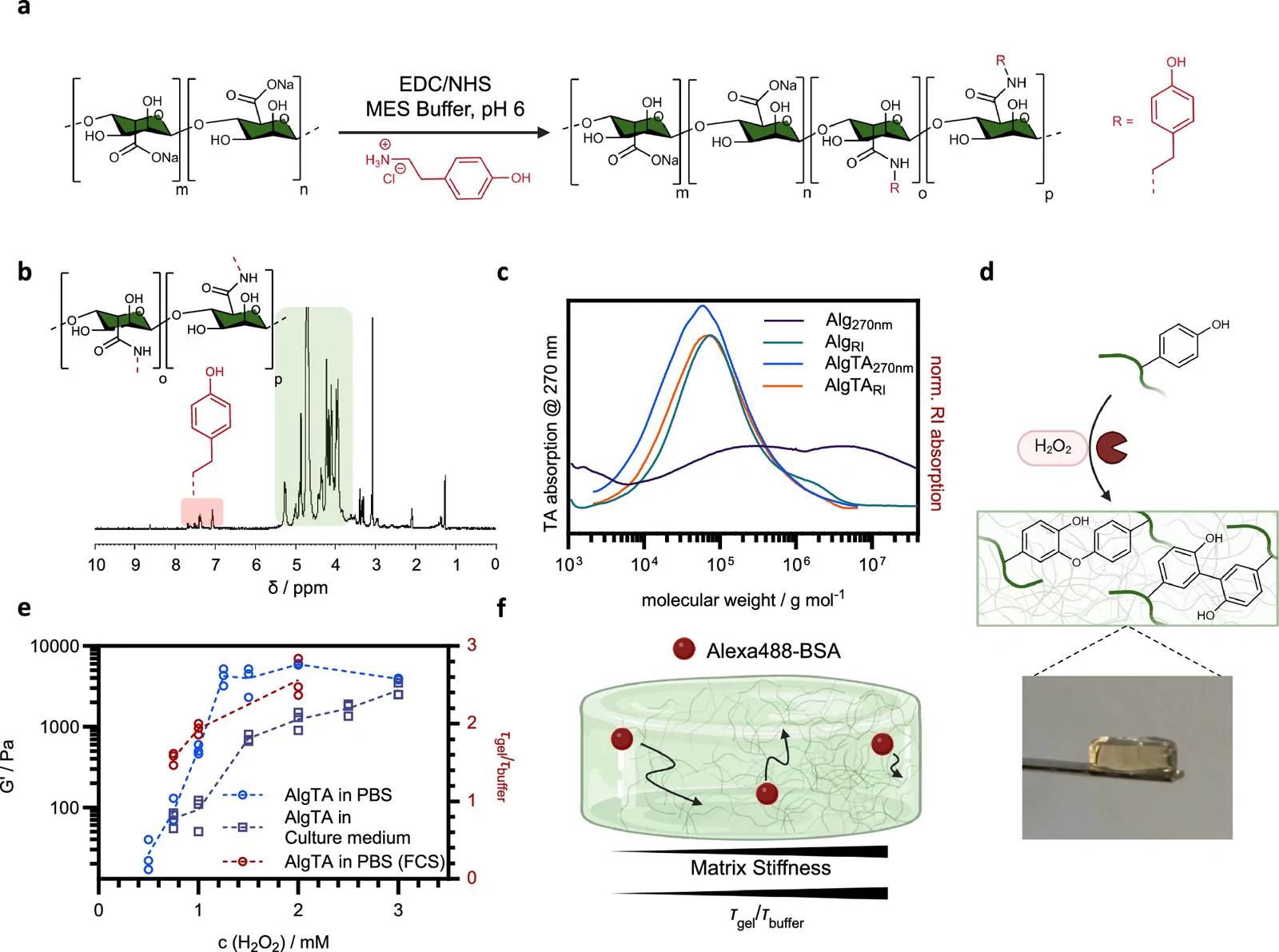
Synthesis and peroxide-triggered gelation of phenol-functionalised alginate (AlgTA). **a** EDC/NHS-mediated coupling of tyramine to sodium alginate introduces pendant phenols to generate AlgTA. **b** ^1^H NMR spectrum (700 MHz, D_2_O, 323 K); aromatic tyramine resonances (7.10–7.60 ppm) were used to quantify the degree of substitution. **c** Aqueous SEC/GPC traces (RI and UV 270 nm) for alginate and AlgTA (eluent: 0.07 M Na_2_HPO_4_; pullulan standards; conventional calibration). Co-elution with the UV signal supports polymer-bound tyramine; the small apparent molar-mass shift is consistent with low substitution and altered hydrodynamic volume after functionalisation. **d** H_2_O_2_/HRP-mediated oxidative coupling forms covalent crosslinks, yielding gels with tuneable stiffness. Created in BioRender. Braun, M. (2026) https://BioRender.com/569s2eu. **e** Plateau storage modulus *G*′ of AlgTA as a function of H_2_O_2_ concentration. Formulations: 3 wt% AlgTA, 10 U mL^−1^ HRP; gels formed in PBS or in DMEM supplemented with FBS (*n* = 3 independent gel formulations). Diffusion slowdown in AlgTA as a function of H_2_O_2_ concentration measured by fluorescence correlation spectroscopy (FCS); diffusion-time ratio (*τ*_gel_/*τ*_buffer_) determined in PBS-formed gels (3 wt% AlgTA, 10 U mL^−1^ HRP). This calibration links matrix stiffness to molecular transport and provides the basis for spatial FCS mapping in cell-driven gelation experiments. Data are mean ± s.d., with individual values shown (*n* = 3 positions per gel). Connecting lines are visual guides only; points show individual replicates. **f** Relationship between matrix stiffness and diffusion slowdown measured by fluorescence correlation spectroscopy (FCS). Created in BioRender. Braun, M. (2026) https://BioRender.com/569s2eu.

Phenol and catechol chemistries are central motifs in bioinspired polymer design and living materials. Their robust oxidative coupling under biological conditions, exemplified by mussel adhesion, cuticle hardening and melanin formation^16–18^, has motivated their integration into covalently crosslinkable hydrogels^13^. To translate this chemistry into a metabolism-responsive matrix material, we designed and synthesised a phenol-functionalised alginate (AlgTA) by carbodiimide-mediated conjugation of tyramine to sodium alginate (1-ethyl-3-(3-dimethylaminopropyl)carbodiimide/N-hydroxysuccinimide, EDC/NHS chemistry; modified from Schulz et al.^13^), introducing pendant phenols along the polysaccharide backbone (Fig. 2a). The product was purified by extensive dialysis to remove residual reagents and soluble side-products and isolated in >95% yield across three independent syntheses. The degree of substitution (DS) averaged ~2.5 mol%, with narrow batch-to-batch variation, as determined by ¹H NMR spectroscopy (2.3–2.6 mol%). This low but reproducible modification density preserves polymer solubility while providing pendant phenols for enzymatic oxidative coupling.

¹H NMR spectroscopy confirmed successful tyramine conjugation (Fig. 2b). Aromatic resonances at 7.10–7.60 ppm integrated relative to the guluronic-acid anomeric signal at 5.35 ppm yielded a DS of ~2.5  mol% tyramine per uronate unit. (Supplementary Figs. 1 and 2, Supplementary Equations 1 and 2; further discussion of alginate characterisation and functionalisation is provided in the Supporting Information)^19^. ¹H diffusion-ordered NMR (DOSY) further supported polymer-bound tyramine by showing that the aromatic signals diffuse with the alginate backbone (D ≈ 10^−10.5^ m^2^ s^−1^; Supplementary Fig. 3). Minor resonances at 3.40–3.30, 2.10, and 1.27 ppm were assigned to residual EDC-derived N-acylurea side-products, as reported^20^. Importantly, the purified AlgTA material used in subsequent experiments, including these residual signals, retained HRP-mediated crosslinkability and showed no detectable reduction in cell viability under the tested material-exposure conditions (Supplementary Figs. 4–7).

Size-exclusion chromatography (SEC/GPC) further supported polymer-bound tyramine: co-elution of the alginate refractive index and UV (270 nm) signals indicated that the aromatic signal was associated with the polymer fraction, with a small shift in apparent molar mass from 68.8 ± 2.9 kg mol^−1^ (alginate) to 60.0 ± 0.9 kg mol^−1^ (AlgTA), consistent with the low degree of substitution (Fig. 2c, Supplementary Table 1). In the presence of hydrogen peroxide and horseradish peroxidase, pendant phenols undergo oxidative coupling to form covalent network junctions (Fig. 2d). Using defined H_2_O_2_ concentrations first decoupled the crosslinking reaction from cellular metabolism and established the accessible mechanical and transport-response window of AlgTA gels before introducing lactate-producing cells. This reaction therefore provides the chemical basis for metabolite-gated gel formation, in which cell-derived lactate is enzymatically converted to H_2_O_2_ to initiate polymer crosslinking.

Peroxide-mediated crosslinking defines the mechanical window for lactate-gated gelation. To translate metabolic activity into material formation, we first examined peroxide-mediated crosslinking of AlgTA (~2.5 mol% tyramine per uronate unit) at 3 wt%. AlgTA and horseradish peroxidase (HRP, 10 U mL^−1^) were mixed and exposed to 0.5–3 mM hydrogen peroxide to yield covalently crosslinked hydrogels within 30 min (Supplementary Fig. 8). Gelation kinetics and mechanics were monitored by oscillatory shear rheology. Strain-sweep experiments defined the linear viscoelastic region, frequency sweeps characterised the final network response across different timescales and time sweeps monitored gel formation at 1% strain and 1 Hz (Supplementary Figs. 9 and 10). The linear viscoelastic region extended to at least 10% strain, and the gels showed a predominantly frequency-independent storage modulus (*G*′), consistent with formation of an elastic covalent network. At 0.75 mM H_2_O_2_, *G*′ remained frequency-independent up to 2 Hz, whereas this response extended to 10 Hz at full crosslinking (3 mM). The plateau storage modulus extracted from time sweeps (1% strain, 1 Hz) revealed a pronounced H_2_O_2_ dose response (Fig. 2e). Control experiments lacking either HRP or H_2_O_2_ remained at *G*′ ≈ 10 Pa, comparable to unmodified 3 wt% alginate, demonstrating that both enzymatic oxidation and peroxide fuel are required for covalent network formation. Based on ~3.5 mM phenol groups per 3 wt% AlgTA, complete phenol pairing would correspond to a maximum junction concentration of ~1.75 mM H_2_O_2_ (two phenols per covalent junction). Consistent with this stoichiometric estimate and reproducible across batches, gels stiffened sharply between 0.5 mM and 1.5 mM H_2_O_2_: *G*′ increased from 30 ± 10 Pa to 4.8 ± 0.5 kPa, approaching a plateau at ~5 kPa (Fig. 2e).

To benchmark the rheological behaviour under cell-culture conditions, gelation was performed in DMEM (Dulbecco’s Modified Eagle’s Medium) supplemented with 10% foetal bovine serum (FBS) and 1% penicillin/streptomycin. Detectable elastic gels formed at H_2_O_2_ concentrations ≥0.75 mM, reaching *G*′ = 70 ± 20 Pa at 0.75 mM and 2.8 ± 0.5 kPa at 3 mM. The attenuated stiffening in medium is consistent with phenolic competitors and peroxide scavenging: DMEM contains ~0.4 mM tyrosine, and FBS proteins contribute additional phenolic residues that can compete for HRP-mediated oxidation and consume peroxide-derived oxidants. Despite these interferences, the resulting stiffness range from sub-kPa to several kPa spans mechanical regimes relevant to lung and fibrotic tissues^21–23^. The hydrogels remained stable under cell-culture conditions and showed no detectable degradation upon incubation with cell lysate over 12 days (Supplementary Figs. 11 and 12).

To implement the full metabolite-responsive cascade, AlgTA was combined with HRP (10 U mL^−1^) and lactate oxidase (LOx, 1 U mL^−1^). Upon addition of sodium lactate (10 mM), rheology revealed gelation after a short induction period (Supplementary Fig. 13). The low initial modulus before the rise in *G*′ is consistent with a physically entangled AlgTA solution, whereas the subsequent increase in *G*′ reflects accumulation of covalent phenol junctions. Because LOx produces H_2_O_2_ enzymatically upstream of HRP-mediated phenol coupling, gelation proceeded more slowly than with direct H_2_O_2_ addition but reached comparable plateau moduli for equivalent oxidant yield (Supplementary Fig. 8). The low LOx loading was selected to limit extracellular peroxide accumulation while preserving mechanical tunability for subsequent cell experiments.

#### FCS maps local transport as a readout of network density

While classical oscillatory rheology reports bulk behaviour, it cannot resolve local heterogeneities under culture conditions. We therefore employed fluorescence correlation spectroscopy (FCS) as a local probe of network density and transport restriction^24–26^. FCS quantifies fluorescent tracer diffusion within a confocal sub-femtolitre volume, providing a sensitive readout of mesh size and molecular transport (Fig. 2f)^24,25,27^. Alexa Fluor 488–labelled bovine serum albumin (BSA, hydrodynamic radius ≈3.5 nm) served as tracer. Gels were equilibrated for 24 h in PBS containing the tracer prior to measurement. Autocorrelation curves (Supplementary Fig. 14) were fitted (Eq. 1) to extract diffusion times (*τ*), and diffusion slowdown was expressed as **τ**_gel_/***τ***_buffer_. Diffusion was hindered progressively with increasing crosslinking: *τ*_gel_/*τ*_buffer_ = 1.6 ± 0.1 for *G*′ = 90 ± 35 Pa, 1.9 ± 0.1 at 520 ± 70 Pa, and 2.6 ± 0.25 at 4.8 ± 0.5 kPa (Fig. 2e). Thus, tracer diffusion slowdown increased monotonically with storage modulus, establishing FCS as a calibrated, spatially resolved readout for local network density in AlgTA hydrogels (Fig. 2f). This calibration provided the basis for mapping cell-driven gelation around metabolically active spheroids.

### Endogenous lactate drives remote gelation and redox signalling

**Fig. 3:**
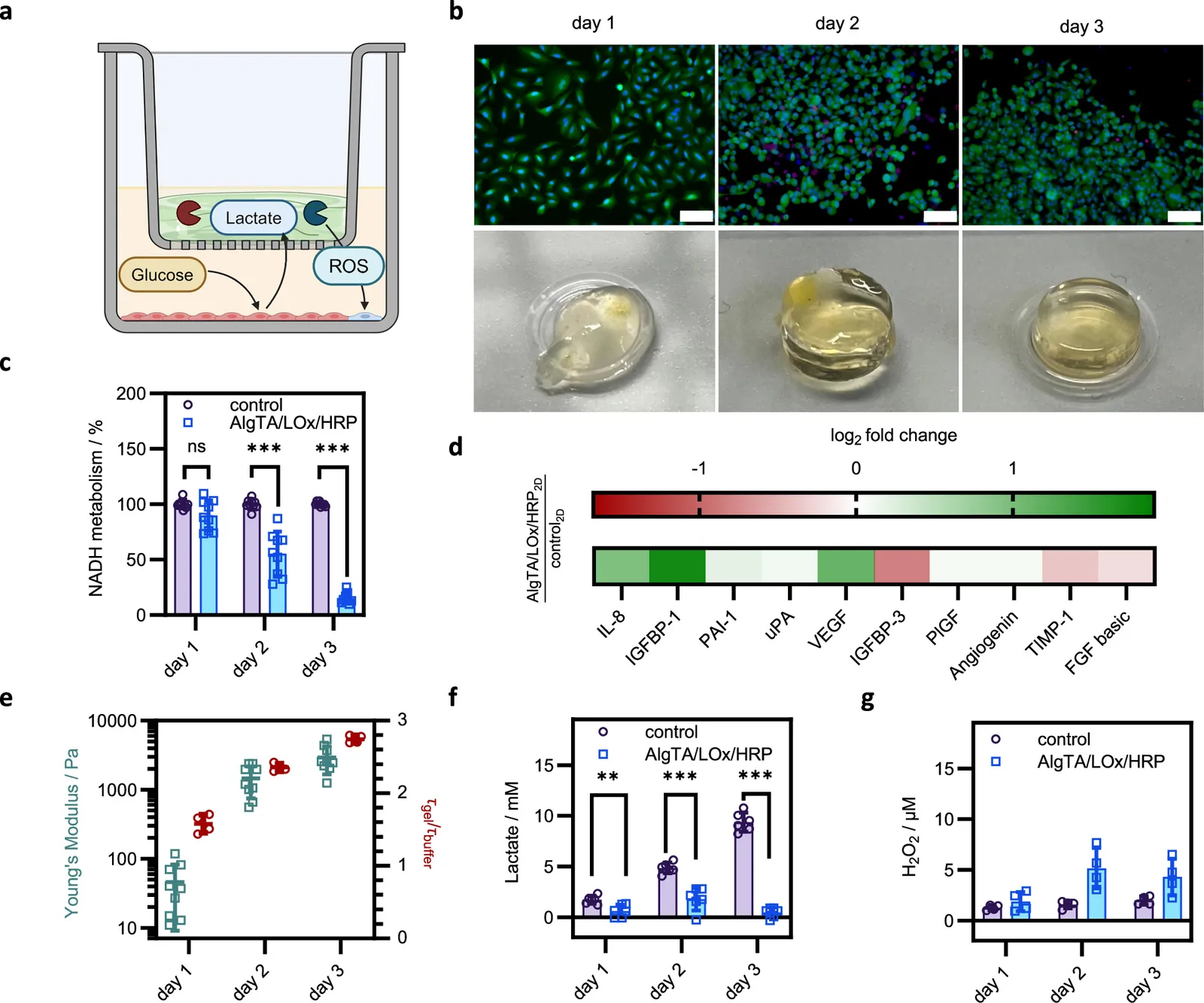
Endogenous lactate drives peroxide-mediated crosslinking and in situ gelation. Unless stated otherwise, data are mean ± s.d. Statistics: unpaired two-tailed Welch’s *t*-test; *P* < 0.05 (*), *P* < 0.01 (**), *P* < 0.001 (***), *P* > 0.05 (ns, not significant). **a** Transwell assay schematic: A549 cells in the lower well; AlgTA + lactate oxidase (LOx) + horseradish peroxidase (HRP) in the insert. Cell-secreted lactate diffuses into the insert and is oxidised by LOx to generate H_2_O_2_, which HRP uses to oxidatively couple AlgTA phenols and form a gel (see also Fig. 2d). Created in BioRender. Braun, M. (2026) https://BioRender.com/569s2eu. **b** Representative fluorescence micrographs of A549 cells at days 1–3. Stains: Calcein AM (live, green), NucBlue (nuclei, blue), propidium iodide (dead, red), *n* = 3 independent experiments. Scale bars, 100 µm. **c** Resazurin-based metabolic activity (*n* = 9 wells) from 3 independent experiments. Shown *P*-values: day 1, *P* = 0.054, day 2, *P* < 0.001; day 3, *P* < 0.001. **d** Relative proteome profiling of selected tumour-associated proteins in AlgTA/LOx/HRP-treated cultures at day 2 versus matched controls (*n* = 3 biological replicates, 10 wells each, 2 on-membrane replicates). Shades of red indicate decreased, and shades of green indicate increased relative protein abundance. **e** Evolution of mechanics and transport: Young’s modulus *E* measured by nanoindentation (*n* = 9 indentations from 3 gels; see the “Methods” section) and fluorescence correlation spectroscopy (FCS) diffusion-time ratio (*τ*_gel_/*τ*_buffer_) for gels formed at days 1–3 after equilibration in tracer-containing PBS (*n* = 4 positions per gel). **f** Lactate concentration under the indicated conditions at days 1–3. (*n* = 6 samples from 2 independent experiments). Shown *P*-values: day 1, *P* = 0.005; day 2, *P* < 0.001; day 3, *P* < 0.001. **g** Extracellular H_2_O_2_ quantified by a fluorimetric hydrogen peroxide assay at days 1–3 (*n* = 4 samples).

To test whether cell metabolism alone can initiate material formation, we assessed endogenous lactate-driven gelation using A549 lung adenocarcinoma cells, a highly glycolytic model with elevated lactate production under aerobic glycolysis^28^. Glucose-deprived cultures served as negative controls, as lactate production depends on glucose metabolism^29^. To physically separate gel formation from the cell layer and initially exclude direct mechanobiological feedback from an adjacent gel, AlgTA, LOx, and HRP were confined to a Transwell insert, while cells were cultured in the lower chamber (25,000 cells per well; 80–90% confluence by day 4) (Fig. 3a). Lactate quantification confirmed continuous accumulation in glucose-fed cultures, but not in glucose-free controls (Fig. 3f; Supplementary Fig. 15). In the presence of AlgTA/LOx/HRP, lactate was progressively depleted, by 45 ± 25% at 24 h, 58 ± 12% at 48 h, and 93 ± 9% at 72 h, relative to matched cell-only controls, consistent with enzymatic conversion to hydrogen peroxide and pyruvate. Correspondingly, extracellular H_2_O_2_ concentrations increased up to threefold (Fig. 3g; Supplementary Fig. 16), supporting the conclusion that the metabolic flux powered the oxidative cascade.

#### Remote lactate-driven gelation produces mechanically maturing hydrogels

In glucose-containing medium, AlgTA precursors in the Transwell insert gradually transitioned from viscous fluids to self-supporting gels over 1–3 days: weak, viscous networks formed by day 1 (*E* = 50 ± 40 Pa, nanoindentation) matured to stable gels by day 2 (*E* = 1.5 ± 0.7 kPa) and to well-defined hydrogels by day 3 (*E* = 2.9 ± 1.3 kPa) (Fig. 3b, e). Increasing yellow colouration was consistent with progressive phenol oxidation^30^. FCS corroborated this mechanical maturation, with tracer diffusion slowing from *τ*_gel_/*τ*_buffer_ = 1.58 ± 0.13 (day 1) to 2.36 ± 0.05 (day 2) and subsequently 2.75 ± 0.05 (day 3) (Fig. 3e; Supplementary Fig. 17). In glucose-free conditions, no gel formed, consistent with absent lactate and lack of fuel for the enzymatic crosslinking cascade.

#### Lactate-fuelled peroxide generation alters cellular metabolism and stress-associated signalling

As lactate is processed by the AlgTA/LOx/HRP cascade, extracellular H_2_O_2_ accumulates to micromolar concentrations (Fig. 3g), overlapping with the ranges typical of epithelial lining fluids and vascular lumens^31^. Whereas exogenous H_2_O_2_ at ≥100 µM typically induces overt cytotoxicity in A549 epithelial cells^32,33^, the extracellular H_2_O_2_ levels generated here remain within the lower ~1–50 µM range associated with redox signalling and adaptive responses in epithelial cells^34^. Nevertheless, cumulative exposure may lead to cytotoxic effects: metabolic activity (resazurin assay) and relative calcein positive cells decreased progressively under AlgTA/LOx/HRP conditions over 3 days, indicating progressive metabolic suppression and loss of viable cell fraction during prolonged exposure (Fig. 3c, Supplementary Fig. 18). At day 2, relative proteome profiling revealed marked increases in insulin-like growth factor-binding protein 1 (IGFBP-1) and vascular endothelial growth factor (VEGF), accompanied by decreased insulin-like growth factor-binding protein 3 (IGFBP-3) relative to the control. IGFBP-1 upregulation has been linked to activation of mitochondrial superoxide dismutase (SOD2), enhancing oxidative-stress tolerance^35^; loss of IGFBP-3 can remove a growth-inhibitory brake^36^, while elevated VEGF promotes angiogenic signalling to maintain nutrient supply^35,37,38^. Together, these proteomic changes are consistent with reactive oxygen species (ROS)-associated signalling in response to lactate-fuelled, spatially decoupled oxidative crosslinking (Fig. 3d, Supplementary Figs. 19 and 20, Supplementary Table 2).

The 2D cell cultures, together with the remote gelation setup, allow spatially separated analysis of how hydrogel formation and cell-derived signals communicate across space. However, 2D cell monolayers lack the mature extracellular matrix and cell-cell interactions that define tumour architecture. A 3D tumour model is therefore required to assess how lactate-gated gelation shapes the mechanobiological landscape in the immediate vicinity of metabolically active cell aggregates.

### Spheroid-derived lactate builds radial 3D gelation gradients and remodels cell state

#### Lactate-fuelled gelation around spheroids

**Fig. 4:**
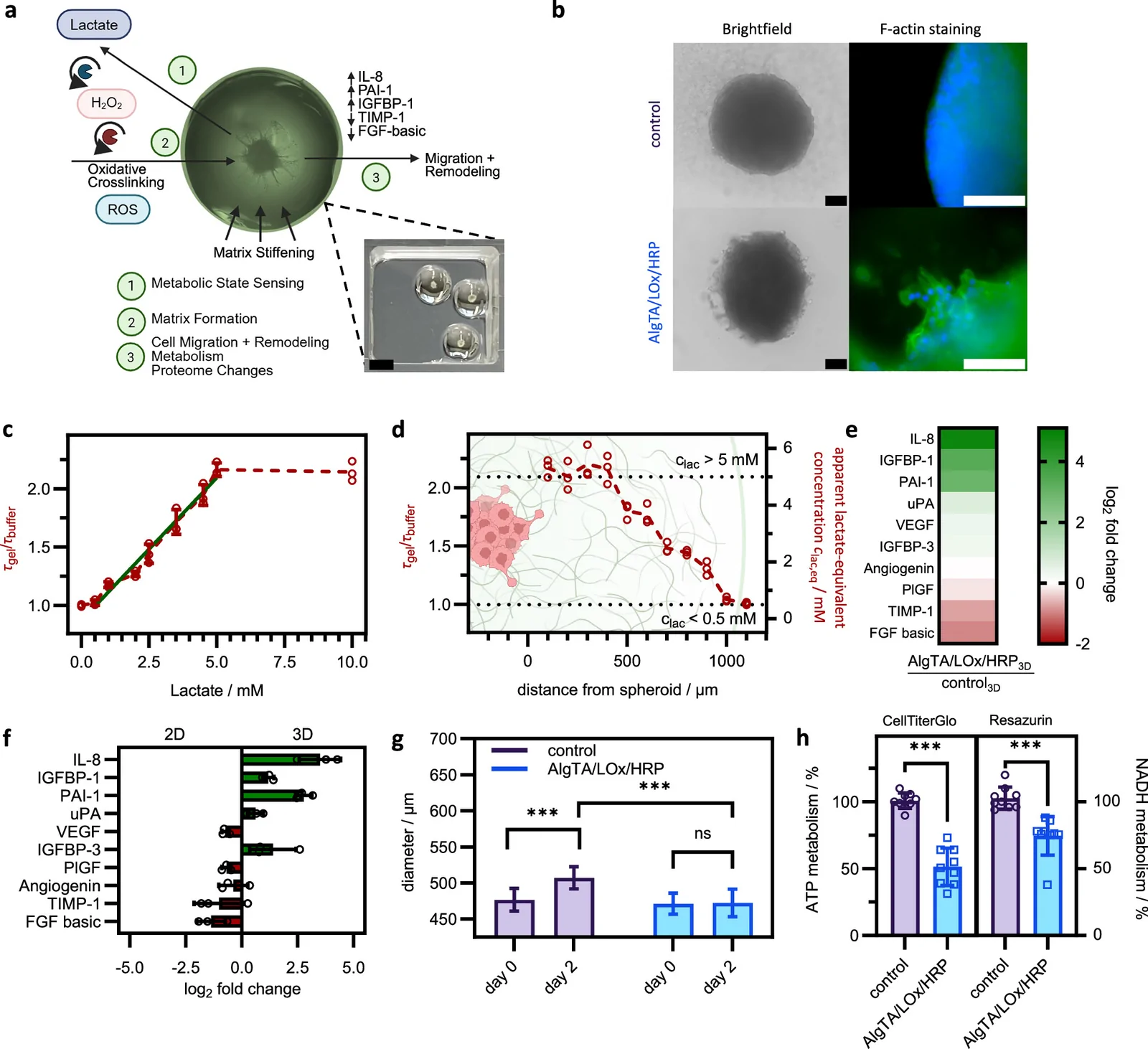
Lactate-gated AlgTA encapsulation of A549 spheroids redirects cell state. Unless stated otherwise, data are mean ± s.d. (day 2). Statistics: unpaired two-tailed Welch’s *t*-test; *P* < 0.05 (*), *P* < 0.01 (**), *P* < 0.001 (***), *P* > 0.05 (ns, not significant). Formulation: 3 wt% AlgTA, 10 U mL^−1^ HRP, 1 U mL^−1^ LOx; control: DMEM supplemented with FBS. **a** Communication cascade: spheroid-secreted lactate is converted by LOx/HRP to H_2_O_2_, driving phenol coupling and in situ AlgTA gelation around the spheroid (schematic) and photograph of encapsulated spheroids. Scale bar, 2 mm. Created in BioRender. Braun, M. (2026) https://BioRender.com/569s2eu. **b** Representative fluorescence micrographs of A549 spheroids: F-actin (phalloidin, green) and nuclei (NucBlue, blue), *n* = 3 independent experiments. Top: control (regular medium). Bottom: AlgTA/LOx/HRP, showing actin-rich protrusions. Scale bars, 100 µm. **c** Calibration of hydrogel crosslinking with defined lactate concentrations (green). Correlation between the diffusion-time ratio of Alexa488-BSA (*τ*_gel_/*τ*_buffer_) and known concentrations of lactate (0–10 mM sodium lactate). **d** Spatial transport mapping by FCS: diffusion-time ratio (*τ*_gel_/*τ*_buffer_) and apparent lactate-equivalent concentration (*c*_lac_,_eq_) *versus* distance from the spheroid surface. Individual values were taken at three parallel offset distances and are shown with a line representing the mean. **e** Proteomics (3D, day 2): relative abundance of selected tumour-associated proteins in AlgTA/LOx/HRP versus control (*n* = 3 biological replicates, 12 spheroids each, 2 on-membrane replicates). Red indicates decreased, and green indicates increased relative protein abundance. **f** Proteomic profiling of treated AlgTA/LOx/HRP 2D Transwell and 3D spheroid cultures. Filled bars indicate the condition showing the stronger relative response. 3D: 3 biological replicates, 12 spheroids each, 2 on-membrane replicates; 2D: *n* = 3 biological replicates, 10 wells each, 2 on-membrane replicates. **g** Representative spheroid diameter with and without AlgTA/LOx/HRP; 16 spheroids per condition. Shown *P*-values: AlgTA/LOx/HRP day 0 vs day 2, *P* = 0.546; control day 0 vs day 2, *P* < 0.001; AlgTA/LOx/HRP day 2 vs control day 0, *P* < 0.001, *n* = 3 experiments. **h** Metabolism under encapsulation (*n* = 3 independent experiments): CellTiter-Glo® 3D (ATP, *n* = 9 wells, *P* < 0.001) and resazurin (NADH, *n* = 9 wells, *P* < 0.001) assays.

Unlike in 2D cell cultures, where lactate dissipates into the bulk medium, 3D tumour spheroids generate steep gradients that radiate from the surface of these densely packed cell clusters. When embedded in DMEM containing AlgTA, LOx, and HRP, spheroids locally converted lactate to hydrogen peroxide, initiating oxidative coupling of AlgTA phenols and forming a covalently crosslinked pericellular gel within one day. As lactate accumulated and was enzymatically consumed, gelation progressed outward, yielding stable, self-supporting gel–spheroid composites (Fig. 4a). After two days, the mean gel–spheroid composite diameter reached 2100 ± 100 µm (Supplementary Fig. 21).

#### FCS resolves radial network density and lactate-equivalent gradients

To convert diffusion slowdown into a lactate-dependent crosslinking readout, (*τ*_gel_/*τ*_buffer_) was calibrated using preformed gels generated with defined lactate concentrations under matched LOx/HRP conditions. We therefore report an apparent lactate-equivalent concentration (*c*_lac,eq_), rather than a true time-integrated concentration (Fig. 4c). Diffusion slowdown (*τ*_gel_/*τ*_buffer_) served as a local proxy for network density. Within 100–400 µm from the spheroid surface, *τ*_gel_/*τ*_buffer_ = 2.2 ± 0.1 indicated a densely crosslinked network with a lactate-equivalent concentration *c*_lac,eq_ ≥ 5 mM (Fig. 4d). The ratio gradually decreased to 1.8 ± 0.1 and to 3.8 ± 0.3 mM *c*_lac,eq_ at 500 µm reaching 1.00 ± 0.01 corresponding to *c*_lac,eq_ ≤ 0.5 mM at 1000 µm, marking the outer gel boundary. Spheroid-derived lactate thus spatially instructs covalent crosslinking and defines the hydrogel architecture within its microenvironment.

#### Local gelation redirects proteomic and morphological responses

The proteomic response under lactate-gated 3D encapsulation differed markedly from that observed in the separated 2D Transwell experiments. In 2D, cells were exposed to diffusible H_2_O_2_ generated by the AlgTA/LOx/HRP cascade, leading to a pronounced redox-signalling signature. In 3D spheroids, however, locally produced H_2_O_2_ can be rapidly consumed during phenol coupling at the spheroid surface, which limits peroxide accumulation and shifts the dominant cue toward pericellular network formation, transport restriction and mechanical confinement. Consistent with this, relative-abundance proteomics revealed a distinct expression profile during 3D encapsulation (Fig. 4e, Supplementary Figs. 20 and 23, Supplementary Table 2): IL-8 (Interleukin-8) increased strongly, whereas IGFBP-1, IGFBP-3, and PAI-1 (plasminogen activator inhibitor-1) showed moderate upregulation. VEGF remained essentially unchanged, while tissue inhibitor of metalloproteinases-1 (TIMP-1) and basic fibroblast growth factor (bFGF) decreased significantly. Functionally, elevated IL-8 and PAI-1 are associated with a pro-inflammatory and pro-migratory programme, and reduced TIMP-1 may relieve metalloproteinase inhibition to promote matrix remodelling^37,39,40^. The stable VEGF levels, in contrast to the strong VEGF induction in 2D, support the interpretation that soluble ROS-mediated signalling is attenuated in 3D, where peroxide is locally consumed by the crosslinking reaction rather than accumulating in the bulk medium (Fig. 4f)^41^.

Morphologically, phalloidin staining revealed actin-rich protrusions and invasive extensions from spheroid edges only in the presence of AlgTA/LOx/HRP, consistent with a shift from growth-dominated behaviour toward migration- and matrix-remodelling-associated phenotypes in response to the pericellular gel network^42^. Notably, the protrusions were absent in softer gels (1 or 2 wt% AlgTA; 35 ± 9 or 600 ± 100 Pa), suggesting that a threshold of mechanical stiffness is required to trigger these morphogenetic changes (Supplementary Figs. 24 and 25)^43–45^.

To isolate the chemical contribution of H_2_O_2_ to this encapsulation response, HRP was replaced with catalase (Cat), which decomposes H_2_O_2_ into water and oxygen and thereby prevents phenol coupling. Eliminating oxidative crosslinking abolished gel formation and yielded proteomic profiles closely resembling untreated controls (Supplementary Figs. 20, 26 and 27, Supplementary Table 2).

#### Pericellular gelation suppresses spheroid expansion and metabolic activity

**Fig. 5:**
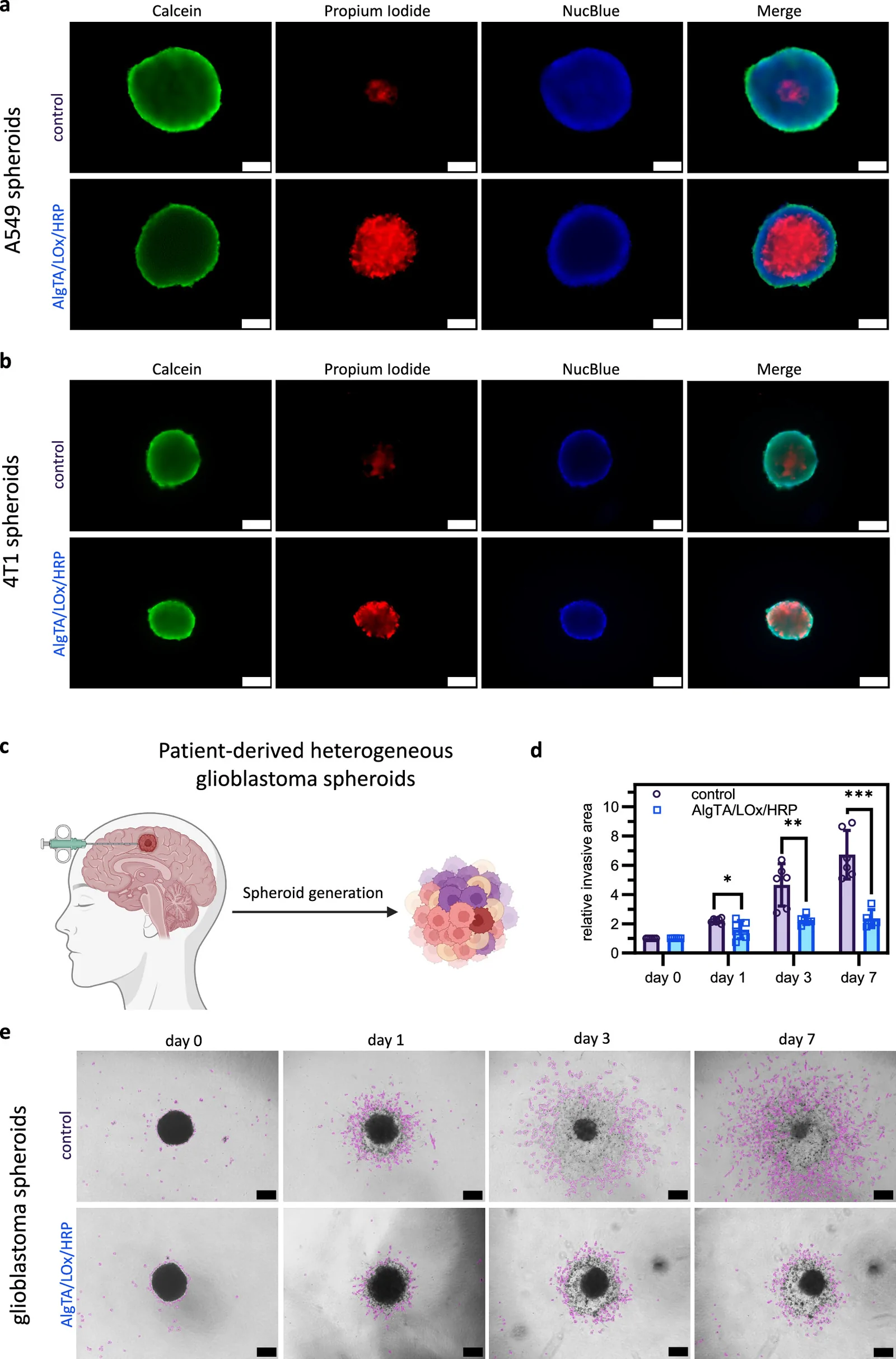
Applicability across glycolytic tumour models. Unless stated otherwise, data are mean ± s.d. (day 2). Statistics: unpaired two-tailed Welch’s *t*-test; *P* < 0.05 (*), *P* < 0.01 (**), *P* < 0.001 (***), *P* > 0.05 (ns, not significant). Formulation: 3 wt% AlgTA, 10 U mL^−1^ HRP, 1 U mL^−1^ LOx; control: medium without AlgTA/LOx/HRP. **a** Representative live/dead staining micrographs of lactate-gated AlgTA-encapsulated A549 spheroids after 2 days of incubation (*n* = 3 independant experiments). Stains: Calcein AM (live, green), NucBlue (nuclei, blue), propidium iodide (dead, red). Scale bars, 200 µm. **b** Representative live/dead staining micrographs of lactate-gated AlgTA-encapsulated 4T1 spheroids after 2 days of incubation (*n* = 3 independant experiments). Stains: Calcein AM (live, green), NucBlue (nuclei, blue), propidium iodide (dead, red). Scale bars, 200 µm. **c** Formation of patient-derived heterogeneous glioblastoma spheroids. Following surgical tumour resection, glioblastoma specimens are dissociated into single cells, which are subsequently used to generate stable spheroids. Patient-derived material was obtained under institutional ethical approval and informed consent; donor/sample information is provided in the “Methods” section. Created in BioRender. Braun, M. (2026) https://BioRender.com/569s2eu. **d** Invasive area of patient-derived glioblastoma spheroids with and without AlgTA/LOx/HRP after up to 7 days; 6 spheroids per condition. Representative of *n* *=* 3 independent experiments from different donors. Shown *P*-values: control vs. AlgTA/LOx/HRP; day 1, *P* = 0.042; day 3, *P* = 0.003; day 7, *P* < 0.001. **e** Representative brightfield micrographs of patient-derived glioblastoma spheroids after up to 7 days of incubation (*n* = 3 independent experiments). Purple areas indicate the invasive area. Scale bar, 200 µm.

Encapsulation within the AlgTA/LOx/HRP matrix halted spheroid expansion and reduced metabolic activity. In gel-free controls, spheroids grew from 474 ± 18 µm to 507 ± 17 µm over two days (*P* < 0.001), whereas encapsulated spheroids maintained a constant size (468 ± 22 µm to 472 ± 19 µm, *P* = 0.546; Fig. 4g). The proportion of dead cells, marked by propidium iodide, increased substantially and comprised a major fraction of the spheroid (see Fig. 5a). Consistently, ATP and NADH levels measured by CellTiter-Glo® 3D and resazurin assays were reduced to 51 ± 14% (*P* < 0.001) and 75 ± 15% (*P* < 0.001), respectively, compared to gel-free controls at day 2 (Fig. 4h), reflecting suppressed proliferation and diminished metabolic activity under gel confinement.

#### Lactate-gated gelation extends to additional tumour-spheroid models

To evaluate whether lactate-gated gelation extends beyond A549 cells, we applied the AlgTA/LOx/HRP system to 4T1 murine mammary carcinoma spheroids, a highly glycolytic tumour model that also secretes substantial lactate^46^. Under identical conditions (3 wt% AlgTA, 1 U mL^−1^ LOx, 10 U mL^−1^ HRP), stable pericellular gels formed at the spheroid interface, similar to the behaviour observed for A549 spheroids (Supplementary Fig. 28). Unlike A549, however, 4T1 spheroids did not develop actin-rich protrusions or invasive extensions into the surrounding matrix, indicating a tumour-model-specific morphological response to confinement (Supplementary Fig. 29). Growth was similarly suppressed: treated 4T1 spheroids decreased in diameter from 361 ± 9 µm to 306 ± 7 µm over two days (*P* < 0.001; Supplementary Fig. 30). Propidium iodide staining revealed markedly enlarged necrotic cores in both A549 and 4T1 spheroids following encapsulation (Fig. 5a, b; Supplementary Figs. 31 and 32), and calcein staining confirmed reduced viable cell fractions relative to untreated controls.

To further explore the translational relevance of this concept, we evaluated the system using patient-derived heterogeneous glioblastoma spheroids generated from surgically resected tumour material under institutional ethical approval and informed consent (Fig. 5c). Over a culture period of seven days, the material promoted encapsulation of the spheroids and significantly reduced the invasive area compared to the AlgTA-free control (Fig. 5d, e; Supplementary Fig. 33).

These results indicate that lactate-gated crosslinking is not restricted to a single cancer model and can impose growth- or invasion-limiting confinement across distinct tumour-spheroid systems.

#### Metabolic flux is transduced into matrix assembly and tumour-cell state

Together, the 3D results establish a mechanistic link between cellular metabolism and material state and, ultimately, cell phenotype. Endogenous lactate released by the spheroids is enzymatically oxidised by lactate oxidase to hydrogen peroxide, which fuels the horseradish peroxidase–mediated oxidative coupling of AlgTA phenols. The resulting covalent crosslinking defines the stiffness and local transport properties of the hydrogel, and these evolving material states feed back onto cellular signalling and behaviour. Under lactate-gated encapsulation, A549 spheroids shift from expansion-dominated growth toward a migration- and matrix-remodelling-associated phenotype, while the surrounding network simultaneously constrains bulk expansion (Fig. 4b, g). The proteomic signatures are consistent with the material readouts: as crosslink density increases and molecular diffusion decreases (FCS; Figs. 2f, 3e and 4d), cells upregulate programmes associated with matrix remodelling, proteolysis, and invasion. Although the findings arise from an in vitro model, they demonstrate that metabolic flux can be transduced into covalent material assembly that, in turn, reshapes cell state. This feedback loop, in which metabolism directs crosslinking, crosslinking establishes mechanics and transport, and the resulting matrix state modulates phenotype, recapitulates a core principle of extracellular-matrix reciprocity in a bio-synthetic system.

## Discussion

This work establishes a materials design principle in which cellular metabolism sets covalent crosslinking kinetics, crosslinking defines matrix mechanics and transport, and these material states feed back to modulate cell phenotype. Leveraging lactate, a hallmark of the tumour microenvironment, enables cell-state-specific, spatially patterned gelation that couples material formation directly to metabolic activity. In spatially separated 2D cultures and 3D tumour spheroids, endogenous lactate generated by A549 cells is oxidised by the LOx–HRP cascade to hydrogen peroxide, which oxidatively couples AlgTA phenols to form a covalent hydrogel. Mechanics and transport evolve in tandem: the storage modulus is tuneable from ~10 Pa to ~5 kPa, gelation propagates approximately 1 mm from spheroid surfaces, and fluorescence correlation spectroscopy maps diffusion gradients that reflect local network density and apparent lactate-equivalent concentration, linking metabolic flux semi-quantitatively to matrix architecture and molecular transport.

Relative to HRP/H_2_O_2_ hydrogels dependent on exogenous oxidants or glucose-oxidase fuelling, lactate-gated gelation places endogenous metabolism upstream of material formation, conferring both spatial selectivity and temporal synchrony with metabolic activity. Compared with enzyme-instructed supramolecular assemblies, this approach yields covalent networks with mechanically stable properties that nevertheless remain responsive to cell-derived fluxes. In 3D, relative proteome profiling and morphological readouts indicate that lactate-gated encapsulation suppresses spheroid expansion while inducing features associated with migration and matrix remodelling, including altered abundance of remodelling-associated proteins and actin-rich protrusions. The data also reveal compartment-dependent signalling. In the Transwell geometry, diffusible H_2_O_2_ generated by the AlgTA/LOx/HRP cascade produces a redox-associated stress and adaptation signature. In spheroids, by contrast, locally generated H_2_O_2_ is consumed during phenol coupling near the spheroid surface, shifting the dominant feedback from soluble redox signalling toward pericellular network formation, transport restriction and mechanical confinement. Because prolonged exposure reduced metabolic activity and viable cell fraction, the system should not be described as intrinsically non-cytotoxic; rather, it provides a material response that is compatible with cells over the early gelation window, followed by exposure- and confinement-associated metabolic suppression in tumour models.

Because gelation is gated by a disease-linked metabolite, this bio-synthetic chemistry suggests the possibility of autonomous, state-selective barriers that form preferentially in lactate-rich tumour niches while remaining inactive below a defined metabolic threshold. The AlgTA/LOx/HRP precursors produce a covalently crosslinked matrix that forms preferentially where local lactate exceeds a tuneable threshold, thereby constraining spheroid expansion and invasion in vitro. Such behaviour could inform strategies to limit local tumour-cell dissemination while modulating the transport of cytokines, nutrients and therapeutic agents. Selectivity could be refined by adjusting enzyme loadings to set a lactate threshold, multiplexing triggers such as lactate and hypoxia for Boolean gating, or co-encapsulating scavengers (for example, catalase) to quench excess reactive oxygen species during gelation. In a potential clinical scenario, such as the resection cavity following surgical removal of malignant glioblastoma tumours, the platform could be developed as a feedback-controlled mechanical containment barrier. Unlike conventional static hydrogels or cavity-filling materials, the system strengthens its network in response to elevated local lactate concentrations associated with highly glycolytic tumour regions. In this context, metabolic activity acts as a trigger for structural adaptation, enabling local matrix formation that may restrict the migration of residual infiltrative cells and therefore motivates evaluation as a locoregional strategy to reduce recurrence risk. We therefore envision this platform as a complementary locoregional strategy that could, in future, be combined with established surgical, chemotherapeutic or immunotherapeutic treatments rather than replacing them.

Translation toward therapeutic settings will require substantial further development. Key requirements include biodegradable or reversibly cleavable linkers for controlled matrix removal, pharmacokinetic and biodistribution studies to assess off-target gelation, evaluation in healthy and tumour-bearing tissue models, mitigation of enzyme immunogenicity, and operation under hypoxic conditions given the oxygen dependence of LOx. Patient-derived glioblastoma experiments support translational relevance, but larger donor cohorts, in vivo validation, immune-competent models and orthogonal validation of proteomic markers will be needed before therapeutic conclusions can be drawn.

Beyond containment, the matrix could be engineered as a metabolite-activated sense-and-respond depot in which gelation controls the retention or release of drugs, cytokines or signalling molecules. Linking metabolic flux to programmable materials behaviour opens routes toward materials that sense biochemical fluxes and respond through changes in network architecture. Near-term extensions include substituting other oxidases to gate gelation by diverse disease-associated metabolites, integrating enzyme cascades for Boolean logic over multiple biochemical inputs, and introducing reversible chemistries to incorporate relaxation and repair. Potential applications span tumour modelling, where mechanical properties are written directly from metabolism, morphogenesis, immuno-engineering in which activated immune cells provide the metabolic cue, and biofabrication processes where on-demand stiffening enhances structural fidelity. More broadly, the ability to translate endogenous biochemical fluxes into covalent network architecture establishes a blueprint for metabolism-addressable living materials that probe, pattern, and steer complex cell–matrix interactions.

## Methods

### Gel formation

Phenol-functionalised alginate (AlgTA, 3 wt%) was dissolved in phosphate-buffered saline (PBS) or Dulbecco’s Modified Eagle’s Medium (DMEM), as indicated for each experiment. Horseradish peroxidase (HRP, stock 100 U mL^−1^) was added to a final concentration of 10 U mL^−1^ and gently mixed to avoid bubble formation. For peroxide-triggered gelation, hydrogen peroxide was added immediately before casting to final concentrations of 0–3 mM (stock 0–30 mM H_2_O_2_). For lactate-triggered gelation, lactate oxidase (LOx, stock 10 U mL^−1^) was additionally included at 1 U mL^−1^, and gelation was initiated by addition of sodium lactate at the indicated concentration. Unless stated otherwise, hydrogels were prepared with final concentrations of 3 wt% AlgTA, 10 U mL^−1^ HRP and either 0–3 mM H_2_O_2_ or 1 U mL^−1^ LOx plus sodium lactate. Peroxide-triggered gels formed within minutes, whereas lactate-triggered gels formed over several hours, reflecting enzymatic H_2_O_2_ generation upstream of HRP-mediated phenol coupling.

### Rheological characterisation

The rheological characterisation was performed using a DHR3 rheometer (TA Instruments; TRIOS software 4.0.2.30774), which was equipped with a temperature controller and a solvent trap to minimise hydrogel dehydration. The experiments were conducted using an 8 mm parallel-plate geometry, with hydrogel samples of 35 µL volume, resulting in a gap size ranging from 0.6 to 0.7 mm. The mechanical properties of the hydrogels were characterised at 25 °C.

Measurements were performed in triplicate from one batch, and data are reported as mean ± s.d. Several experimental procedures were conducted:

(i) Strain Sweep: Oscillatory strain sweeps ranging from 0.01% to 100% strain were performed at a fixed frequency of 1 Hz at 25 °C.
(ii) Frequency Sweep: Frequency sweeps spanning from 0.1 Hz to 10 Hz were executed at a fixed strain of 1% at 25 °C.
(iii) Time Sweep: The linear viscoelastic region extended to at least 10% strain at 1 Hz. Oscillatory time sweeps were therefore performed at 1% strain and 1 Hz at 25 °C.

### Gel permeation chromatography (GPC)

GPC measurements were performed on a 1260 Infinity instrument (Polymer Standard Service, Mainz; WinGPC UniChrom V8.40) with UV/Vis (270 nm) and refractive-index detection at 25 °C. The system was operated with 0.07 M Na_2_HPO_4_ at a flow rate of 1 mL/min. Samples were dissolved at a concentration of 1 mg/mL and washed through a PTFE syringe filter (0.45 μm). Each sample injection volume was 200 μL. Suprema columns (Polymer Standard Service, Mainz) with a particle size of 5 μm were used. The GPC system was calibrated using pullulan standards supplied by Polymer Standard Service. The data was processed using WinGPC UniChrom V8.40, and graphs were generated using GraphPad Prism 10.2.2.

### Fluorescence, absorbance and luminescence spectroscopy

Fluorescence, absorbance *&* luminescence spectra were recorded on a SPARK 20 M microplate reader (Tecan Group Ltd; Sparkcontrol V.2.3). Fluorescence measurements were performed in Greiner 384 flat black well plates (Greiner Bio-One, 781906), luminescence measurements in Greiner white half-area well plates (Greiner Bio-One, 675075) and the absorbance measurements in a 384 UV-STAR® well plate (Greiner Bio-One, 781801). Data processing was done using Microsoft Excel and GraphPad Prism 10.2.2.

### Fluorescence correlation spectroscopy (FCS)

FCS experiments were performed using a commercial confocal microscope (LSM 880, Carl Zeiss, ConfoCor3 3.0, Jena, Germany). Alexa Fluor 488–labelled bovine serum albumin (Alexa488-BSA; hydrodynamic radius ≈3.5 nm) was excited with the 488 nm line of an argon laser, fibre-coupled to the microscope and focused into the sample using a C-Apochromat 40×/1.2 W water-immersion objective (Carl Zeiss, Jena, Germany). Emission light was collected through the same objective and, after passing through a confocal pinhole, directed to a spectral detection unit (Quasar, Carl Zeiss), with the detection window set to 500–550 nm. Ibidi µ-Slide 15 Well 3D Glass Bottom were used as sample chambers for both solutions and gels. In the case of spheroid-formed gels, the position of the spheroid was first visualised using confocal laser scanning mode. The FCS observation volume was then positioned at defined locations either within or outside the gel. At each location, five FCS measurements were recorded, with a total acquisition time of 150 s per position. Fluorescence intensity fluctuations, *δI(t)*, were recorded over time and analysed via the autocorrelation function: *G(τ)* = 1 + *δI(t)* · *δI(t* + *τ) >/<I(t)>*^2^. The resulting experimental autocorrelation curves were fitted using a standard analytical model function^4^:

1 $$G(\tau )=1+\left[1+\frac{{f}_{T}}{1-{f}_{T}}{e}^{-\tau /{\tau }_{T}}\right]\frac{1}{N}\mathop{\sum }\limits_{i=1}^{m}\frac{{f}_{i}}{\left[1+\frac{\tau }{{\tau }_{{Di}}}\right]\sqrt{1+\frac{\tau }{{S}^{2}{\tau }_{{Di}}}}}$$

Here, *N* is the average number of diffusing fluorescence species in the observation volume, *f*_*T*_ and *τ*_*T*_ are the fraction and the decay time of the triplet state, *f*_*i*_ and *τ*_*Di*_ are, respectively, the fraction and the diffusion time of the *i-th* diffusion component. *S* = *z*_0_/*r*_0_ is the so-called structure parameter, where *z*_0_ and *r*_0_ represent the axial and radial dimensions of the confocal volume, respectively. The fits yielded the corresponding diffusion times *τ*_*Di*_ and through $D=\frac{{r}_{0}^{2}}{4{\tau }_{D}}$ the diffusion coefficients of the fluorescent species. As the value of *r*_0_ depends on the specific characteristics of the optical setup, calibration experiments were performed using a fluorescent tracer with a known diffusion coefficient, i.e., Alexa 488 in water.

While most experimental autocorrelation curves were well described by a single-component diffusion model, a two-component fit (Eq. 1, *m* = −2) was required in some cases - particularly for measurements conducted in stiffer gels. The second component represented a minor fraction (a few per cent) and was characterised by a significantly slower diffusion time, likely reflecting transient immobilisation of a subset of Alexa488-BSA tracers within the gel matrix^47^. Data processing was done using Microsoft Excel and GraphPad Prism 10.2.2.

### Calibration of hydrogel crosslinking with defined lactate concentrations

Hydrogels of 10 µL per well were prepared in Ibidi µ-Slide 15 Well 3D glass-bottom chambers using 3 wt% AlgTA, 10 U mL^−1^ HRP and 1 U mL^−1^ LOx in DMEM supplemented with FBS. Sodium lactate was added at defined final concentrations of 0–10 mM (stock 0–100 mM). After 6 h, 30 µL of Alexa488-BSA (50 µM) in PBS was added on top of the gels and samples were incubated overnight to allow tracer equilibration. The next day, the diffusion-time ratio of Alexa488-BSA (*τ*_gel_/ *τ*_buffer_) was measured by FCS at 3 positions in the gel. The calibration curve between 0.5 and 5 mM lactate followed the linear equation y = 0.245× + 0.869 with *R*² = 0.9625. This calibration was used to estimate an apparent lactate-equivalent concentration, *c*_lac,eq_, in spheroid-derived gels.

### Nanoindentation

Formed hydrogels were removed from the well plate inserts by carefully cutting the membrane along its curvature. The hydrogel was then detached from the cut-out membrane and placed on a poly-L-lysine (Poly-L-lysine, P4707, Sigma) coated 12-well plate (LABSOLUTE, 7696791, Th. Geyer GmbH & Co. KG). The poly-L-lysine coating was prepared one day in advance by pipetting 500 µL into the centre of each well and allowing the solution to evaporate overnight at room temperature in a fume hood. The hydrogel was allowed to sit on the coated well plate for 2 min. Afterwards, the well plate was filled with PBS, and measurements were conducted using a Pavone Nanoindenter with a spherical probe (radius = 47 µm, spring constant = 0.4 N m^−1^) (both Optics11). Samples were measured at room temperature, with an approach speed of 10 μm s^−1^. Young’s moduli were derived by fitting the obtained force-indentation curves to a Hertz model up to an indentation depth of 5 µm. The contact point was determined at 20% of the maximum force measured. The Poisson ratio was assumed to be that of an incompressible material (*ν* = 0.5). Data analysis was performed using the software DataViewer (V 2.5.7, Optics11). Technical triplicates were prepared for each day of incubation, and each replicate was measured three times, resulting in a total of 9 measurements per day.

2 $$F(\delta )=\frac{4}{3}\frac{E}{(1-{\nu }^{2})}\sqrt{R}\,{\delta }^{\frac{3}{2}}$$

*F* = Force, *E* = Young’s Modulus, *ν* = Poisson’s Ratio, *R* = Tip Radius, *δ* = Indentation Depth.

### Nuclear magnetic resonance (^1^H NMR)

For the ^1^H NMR experiments and the diffusion measurements (DOSY)^48^, a 5 mm triple resonance QXI ^1^H/^13^C/^15^N/^19^F probe equipped with a z-gradient on the 700 MHz Bruker AVANCE NEO system was used. The spectra were measured at 50 °C for functionalisation degree determination and at 90 °C for G/M ratio calculation. The solvent signal of deuterated water was used as a reference (4.79 ppm).

Proton measurements were performed either with water suppression using gradient excitation sculpting or as a normal single-pulse experiment (zg30) with a relaxation delay of 5 s. The number of scans depended on the signal-to-noise ratio and ranged from 8 to 64 with a 90° pulse of 14 μs and a spectral width of 14,000 Hz (20 ppm). The proton and 2D DOSY (^1^H) spectra were referenced to the residual HDO at 4.70 ppm (δ(^1^H)).

The spectroscopic widths of the homonuclear 2D-COSY (cosygpprqf) experiments with water presaturation were typically 16,000 Hz in both dimensions (f1 and f2), and the relaxation delay 2 s.

For the diffusion measurements, a 2D sequence (DOSY, dstebgp3s)^49,50^ was used, with a 2D sequence for diffusion measurement using double stimulated echo for convection compensation and LED using bipolar gradient pulses for diffusion using 3 spoil gradients^49,50^. The temperature was kept at 323 K for all experiments and regulated by a standard ^1^H methanol NMR sample using the TopSpin 4.50 software (Bruker) to ensure the correct temperature (chemical shift distance in ppm from OH and CH_3_ is temperature dependent). The control of the temperature was realised with a VTU (variable temperature unit) with an accuracy of ±0.1 K.

In this work, the gradient strength was varied in 16 steps from 2% to 100%. The diffusion time was optimised at 60 ms and the gradient length at 1.4 ms. The gradient strength was calibrated by analysis of a sample of ^2^H_2_O/^1^H_2_O at a defined temperature and comparison with the theoretical diffusion coefficient of ^2^H_2_O/^1^H_2_O. Diffusion gradient amplitudes were varied linearly from 1 to 53 G/cm (10 to 470 mT/m) over a total of 16 experiments to achieve a strong diffusion weighting. Diffusion coefficients were then calculated for the integrated peak areas using an exponential decay fit function over the 16 spectra (gradients)

3 $${S}_{i}={S}_{0}\cdot \exp \left(-D\cdot {b}_{i}\right)$$

where the diffusion sensitivity factor *b* was calculated as given in the sequence description:

4 $${b}_{i}={\left(2\pi \cdot \gamma \cdot {G}_{i}\cdot \delta \right)}^{2}\cdot \left(\triangle -\frac{\delta }{3}\right)$$

The data were processed with MestReNova 15.0.1 or Bruker TopSpin 3.6.1.

### Electrospray ionisation (ESI)

EDC/NHS model coupling solutions were analysed using an Advion CMS with an electrospray ionisation (ESI) source in positive mode. Measurements were performed under low-fragmentation conditions. Raw data were processed using Advion Data Express 4.0.13.8, and figures were generated in GraphPad Prism 10.2.2.

### Cell culture

A549 human lung adenocarcinoma cells (DSMZ, ACC 107) and 4T1 murine mammary carcinoma cells (ATCC, CRL-2539) were cultured at 37 °C and 5% CO_2_ in DMEM high glucose (Gibco 31053-028) supplemented with 10% dialysed foetal bovine serum (FBS, Gibco A33820-01) and 1% penicillin/streptomycin. Cells were maintained in T75 flasks and passaged two to three times per week. STR analysis according to the global standard ANSI/ATCC ASN-0002.1-2021 resulted in an authentic STR profile in the reference STR database. Certified free of Mycoplasma contamination when bought. No further tests for Mycoplasma for 4T1. A549 Mycoplasma test performed after purchase. Passages used for experiments are between 3 and 15.

### Spheroid formation (A549)

A549 spheroids were generated by seeding A549 (DSMZ, ACC 107) cells at a density of 5000 cells per well in 50 µL of Dulbecco’s Modified Eagle’s Medium (DMEM, high glucose, Gibco 31053-028) supplemented with 10% dialysed foetal bovine serum (FBS, Gibco A33820-01) and 1% Penicillin/Streptomycin within ultra-low attachment 96-well plates (4520, Corning Inc., New York, USA). The plates were then centrifuged at 200 × *g* for 5 min, and the cells were incubated for one week to allow spheroid formation.

### Spheroid formation (4T1)

4T1 spheroids were generated by seeding 4T1 (ATCC, CRL-2539) cells at a density of 7000 cells per well in 50 µL of Dulbecco’s Modified Eagle’s Medium (DMEM, high glucose, Gibco 31053-028) supplemented with 10% dialysed foetal bovine serum (FBS, Gibco A33820-01) and 1% Penicillin/Streptomycin within ultra-low attachment 96-well plates (4520, Corning Inc., New York, USA). The plates were then centrifuged at 200 × *g* for 5 min, and the cells were incubated for one day to allow spheroid formation.

### Spheroid formation from patient-derived glioblastoma cells

Brain tumour specimens were collected during surgical removal of tumour tissue from patients diagnosed with a high-grade brain tumour. Only specimens with pathological confirmation of glioblastoma diagnosis were included in the study. Patient-derived spheroids were generated from 3 donor specimens according to the previously published protocol^51^. Glioblastoma specimens were dissociated into single-cell suspensions by combined mechanical and enzymatic processing using the Brain Tumor Dissociation Kit (Miltenyi Biotec) supplemented with collagenase II. Following filtration, erythrocyte lysis (0.1 mM EDTA, 10 mM KHCO₃, 0.155 M NH_4_Cl) and debris removal (debris removal solution, Miltenyi Biotec), cells were resuspended in GB-cDMEM (DMEM, 10% heat-inactivated FBS, 2 mM L-glutamine, 10 µg/mL gentamicin and 1 mM MgCl_2_). 20,000 cells per well were seeded into ultra-low attachment BIOFLOAT™ 96-well plates (Sarstedt) and maintained at 37 °C and 5% CO_2_ to allow spheroid formation.

### Lactate-gated gelation in a Transwell

25.000 A549 cells were seeded in a 24-well plate and cultured using 600 µL Dulbecco’s Modified Eagle’s Medium (DMEM, high glucose, Gibco 31053-028) supplemented with 10% dialysed foetal bovine serum (FBS, Gibco A33820-01) and 1% Penicillin/Streptomycin for 24 h. The medium was exchanged, the gel precursor-filled Transwell (Greiner Bio-One 662641) was added, and the experiment started. The Transwells were removed at the selected time and stored in PBS. PBS was exchanged 3 times within 2 h to dilute remaining lactate and hydrogen peroxide. The medium was collected from the wells for analysis of lactate and ROS levels.

### Spheroid-induced formation of hydrogels

The medium of the A549 spheroids in the ultra-low attachment 96-well plates (4520, Corning Inc.) was replaced by AlgTA/LOx/HRP dissolved in Dulbecco’s Modified Eagle’s Medium (DMEM, high glucose) supplemented with 10% dialysed foetal bovine serum (FBS) and 1% Penicillin/Streptomycin. Control spheroids were cultured in Dulbecco’s Modified Eagle’s Medium (DMEM, high glucose) supplemented with 10% dialysed FBS and 1% Penicillin/Streptomycin. The spheroids were cultured for 2 days and analysed.

### Gel stability study

Hydrogels (AlgTA, 3 wt%; H_2_O_2_, 3 mM; HRP, 10 U mL^−1^) were prepared directly in the rheometer geometry, and the storage modulus (*G*′) was measured by oscillatory rheometry at 1% strain and 1 Hz. A549 cells were lysed with M-PER™ (Thermo Scientific, 78503) for 5 min and centrifuged (~14,000 × *g*, 10 min) to remove debris. Hydrogels were incubated in the resulting cell lysate or in lysis buffer (control) for 1, 3, 7 or 12 days at 37 °C and re-measured or photos taken. *G*′ values were normalised to the initial modulus and the corresponding lysate-free control (*G*′_Lysate_/*G*′_PBS_).

### Lactate assay

The calorimetric Lactate Assay Kit (Sigma-Aldrich, MAK329) was performed in a transparent 384-well plate. The samples were taken from the medium of the corresponding experiment and directly used. Following the assay, absorbance intensity was measured using a SPARK 20 M microplate reader (Tecan Group Ltd; Sparkcontrol V.2.3). A lactate calibration curve was obtained, and the data were processed using Microsoft Excel. Graphs were generated using GraphPad Prism 10.2.2.

### Hydrogen peroxide assay

The fluorometric hydrogen peroxide assay Kit (Sigma-Aldrich, MAK165) was performed in a black 384-well plate according to the manufacturer’s instructions. Fluorescence was recorded on a SPARK 20 M microplate reader (Tecan Group Ltd; Sparkcontrol V.2.3). Raw data were processed using Microsoft Excel, and graphs were generated in GraphPad Prism 10.2.2.

### Fluorescence-based cell viability assay (2D)

Cell viability was quantified using a resazurin-based assay (Sigma-Aldrich, TOX8) in black 96-well plates according to the manufacturer’s instructions. Cells were incubated with the resazurin solution for 4 h prior to measurement. Fluorescence was recorded on a SPARK 20 M microplate reader (Tecan Group Ltd; Sparkcontrol V.2.3) with an excitation wavelength of 560 nm and an emission wavelength of 590 nm. Raw data were processed using Microsoft Excel, and graphs were generated in GraphPad Prism 10.2.2.

### Calcein-based cell viability assay (2D)

Cell viability was quantified using Calcein AM (Thermo Fisher, C34852) and Propidium iodide (PI; Sigma-Aldrich, 537060). 25.000 A549 cells were seeded in a 24-well plate and cultured using 600 µL Dulbecco’s Modified Eagle’s Medium (DMEM, high glucose, Gibco 31053-028) supplemented with 10% dialysed foetal bovine serum (FBS, Gibco A33820-01) and 1% Penicillin/Streptomycin for 24 h. The medium was exchanged, the gel precursor-filled Transwell (Greiner Bio-One 662641) was added, and the experiment started. The Transwells were removed at the selected time, and staining stock containing Calcein AM and Propidium iodide was added to the samples, leading to a final concentration of 2 µg/ml Calcein AM and 4 µg/ml PI. The samples were incubated for 20 min and subsequently imaged. Raw data were processed using ImageJ v1.54f and Microsoft Excel; graphs were generated in GraphPad Prism 10.2.2.

### Luminescence-based cell viability assay (3D)

The spheroids were treated with 50 µL 2.5 U/mL alginate lyase (A1603, Sigma-Aldrich) in DMEM at 37 °C for 45 min to remove the gel. All samples, including controls, underwent the same enzymatic treatment. Then, the spheroids were washed with PBS (5×) to remove enzyme and dissolved gel. Cell viability was quantified using the CellTiter-Glo® 3D Luminescent Cell Viability Assay (Promega) in half-area, white 96-well plates according to the manufacturer’s instructions. Luminescence was recorded on a SPARK 20 M microplate reader (Tecan Group Ltd). Raw data were processed using Microsoft Excel, and graphs were generated in GraphPad Prism 10.2.2.

### Fluorescence-based cell viability assay (3D)

The spheroids were treated with 50 µL 2.5 U/mL alginate lyase (A1603, Sigma-Aldrich) in DMEM at 37 °C for 45 min to remove the gel. All samples, including controls, underwent the same enzymatic treatment. Then, the spheroids were washed with PBS (5×) to remove enzyme and dissolved gel. Cell viability was quantified using a resazurin-based assay (Sigma-Aldrich, TOX8) in black 96-well plates according to the manufacturer’s instructions. Spheroids were incubated with the resazurin solution for 4 h prior to measurement. Fluorescence was recorded on a SPARK 20 M microplate reader (Tecan Group Ltd; Sparkcontrol V.2.3) with an excitation wavelength of 560 nm and an emission wavelength of 590 nm. Raw data were processed using Microsoft Excel, and graphs were generated in GraphPad Prism 10.2.2.

### Proteome analysis

Proteome Analysis was performed with A549 2D culture and A549 spheroids after 2 days of incubation. Array data were interpreted as relative, semi-quantitative protein-abundance changes. For 2D cultures, three biological replicates were analysed with two on-membrane technical replicates. For 3D spheroids, three biological replicates were analysed, each consisting of 12 pooled spheroids, with two on-membrane technical replicates. For the 3D spheroid control experiment with AlgTA/LOx/Cat, two biological replicates with two on-membrane technical replicates were performed. Because the array was used for targeted exploratory profiling rather than unbiased quantitative proteomics, protein changes were interpreted together with orthogonal morphological and viability readouts.

### Sample preparation

#### 2D

Cells from 10 wells of a 24-well plate were used for each condition and washed with PBS (2×). The cells were lysed with RIPA buffer (100 µL per well; 39244.01, SERVA Electrophoresis) at 4 °C for 1 h and centrifuged at 4 °C for 15 min (10,000 × *g*).

#### 3D

The spheroids (*n* = 12) were transferred to an Eppendorf tube, washed with PBS (2×) and treated with 200 µL of 2.5 U/mL alginate lyase (A1603, Sigma-Aldrich) in DMEM at 37 °C for 45 min to remove the gel. All samples, including controls, underwent the same enzymatic treatment. Then, the spheroids were washed with PBS (5×) to remove enzyme and dissolved gel. The spheroids were lysed with RIPA buffer (100 µL per spheroid; 39244.01, SERVA Electrophoresis) at 4 °C for 1 h and centrifuged at 4 °C for 15 min (10,000 × *g*).

### Profiling procedure

The resulting supernatants were analysed using the Human Angiogenesis Array (R&D Systems, ARY007) according to the manufacturer’s instructions. Briefly, samples were diluted and incubated with biotinylated detection antibodies before application to the nitrocellulose membranes. After washing, Streptavidin-HRP and chemiluminescent reagents were added. Signal intensity was captured via chemiluminescence (ChemiDoc Imaging System, Bio-Rad, Image Lab Touch Software V. 3.0.1.14), and quantified by calculating the background-subtracted mean pixel density of duplicate spots (“Gels” plugin of ImageJ V.1.54f). The mapping of the array coordinates and the corresponding targets is provided in Supplementary Table 2 and Supplementary Fig. 20. Data were normalised to the untreated control cultured in regular medium without AlgTA/LOx/HRP. The data were processed with Microsoft Excel, and graphs were generated using GraphPad Prism 10.2.2.

### (Fluorescence) Microscopy

Images of the cells were taken using a Keyence BZ-X800 microscope (BZ-X800 Viewer V. 1.3.0.1) using a 10× objective (BZ-PA10, NA 0.45, WD 4.0 mm), 20× objective (BZ-PF20LP, NA 0.45, WD 8.8–7.5 mm) and 40× objective (BZ-PF40LP, NA 0.60 WD 3.3–2.2 mm). Cells and spheroids were stained using NucBlue® Live ReadyProbes® reagent, Calcein AM, Alexa Fluor™ 488 phalloidin and/or propidium iodide, as indicated for each experiment. The fluorescence was imaged with 360/40 nm excitation and 460/50 nm emission filter for NucBlue® Live ReadyProbes® Reagent, 470/40 nm excitation and 525/50 nm emission filter for Calcein AM/Alexa Fluor™ 488 phalloidin and 545/25 nm excitation and 605/70 nm emission filter for propidium iodide. Cells in 2D culture were stained in 600 µL FBS supplemented medium containing 2 µg/ml Calcein AM, 4 µg/ml propidium iodide and 2 drops of NucBlue® Live ReadyProbes® reagent for 20 min. 3D spheroids were stained in 100 µL FBS supplemented medium containing 2 µg/ml Calcein AM, 4 µg/ml propidium iodide and 2 drops/mL of NucBlue® Live ReadyProbes® reagent for 1 h. Alexa Fluor™ 488 phalloidin staining was performed according to the manufacturer's protocol for 1 h after NucBlue® Live ReadyProbes® staining and fixation with pre-warmed paraformaldehyde in PBS (4% Roti-Histofix, Carl Roth) for 60 min.

### Measurement of the spheroid size



Spheroid size was quantified from brightfield images using ImageJ v1.54f. For each spheroid, four diameters were measured along the horizontal, vertical and two diagonal axes at day 0 and after 48 h of incubation. The mean of the four measurements was used as the spheroid diameter, following previous literature^15^. Images were analysed using identical scale calibration and measurement settings for all conditions. The scale bar is 200 µm.

### Invasion assay (patient-derived glioblastoma)

Primary glioblastoma spheroids were embedded into a collagen-based extracellular matrix to evaluate invasive growth. All matrix preparation steps were performed on ice. For each well of a 48-well plate, 200 µL collagen solution consisting of MEM supplemented with 0.02 M NaOH and 2.2 mg/mL bovine collagen was added and pre-incubated at 37 °C for 2 min to initiate matrix formation. Spheroids were subsequently placed centrally onto the semi-polymerised matrix. Following complete polymerisation for 2 h at 37 °C, 200 µL cDMEM (DMEM, 10% heat-inactivated FBS, 10 µg mL^−1^ gentamicin) was added to each well. For lactate-gated gelation, cDMEM contained 3 wt% AlgTA, 1 U mL^−1^ LOx and 10 U mL^−1^ HRP. Control wells received cDMEM only. Invasive outgrowth was monitored by brightfield microscopy using a Leica DM IL LED Fluo microscope equipped with a 5× objective.

### Imaging and quantification of invasive area (patient-derived glioblastoma)

Quantitative image analysis was conducted in CellProfiler using a standardised workflow for all samples. Images were subjected to grayscale conversion, illumination correction and intensity inversion prior to object identification. Spheroid cores and invading cells were identified using the IdentifyPrimaryObjects module with Otsu-based thresholding following logarithmic intensity transformation. The total occupied area of spheroid and invasive regions was quantified while excluding background signals. Accuracy was verified by overlaying detected object outlines onto the original images, and all measurements were normalised to the corresponding spheroid area at day 0.

### Statistics and reproducibility

Data are presented as mean ± s.d. unless stated otherwise. Statistical analyses were performed in GraphPad Prism 10.2.2 or Microsoft Excel. Comparisons between two groups were performed using unpaired two-tailed Welch’s *t*-tests unless stated otherwise. Exact *P*-values are reported where practical; *P* < 0.05 was considered statistically significant. Sample sizes, replicate definitions and numbers of independent experiments are stated in the corresponding figure legends and the “Methods” section. For cell-based assays, biological replicates refer to independent wells, spheroids, gels or independently repeated experiments as indicated; technical replicates refer to repeated measurements within the same biological sample. No statistical method was used to predetermine sample size. No data were excluded unless exclusion criteria are explicitly stated. Experiments were not randomised unless stated otherwise. Image analysis of patient-derived glioblastoma invasion was performed using a standardised CellProfiler pipeline applied identically to all images. Proteome-array data were treated as relative, semi-quantitative measurements and interpreted together with orthogonal viability, morphology and material-characterisation readouts.

### Ethics statement

Institutional Review Board Statement: The study was conducted in accordance with the Declaration of Helsinki and approved by the Ethics Committees of the Medical Association of Rhineland Palatinate, Germany (Nr. 2024-17932-andere Forschung erstvotierend).

Informed Consent Statement: Informed consent was obtained according to the general consent agreement of Universitätsmedizin Mainz from all subjects involved in the study.

### Reporting summary

Further information on research design is available in the Nature Portfolio Reporting Summary linked to this article.

## Supplementary information

Supplementary Information

Reporting Summary

Transparent Peer Review file

## Source data

Source Data

## Data Availability

The data generated in this study have been deposited in the open-access repository, Zenodo, under the accession code https://doi.org/10.5281/zenodo.22081282. Source data are provided with this paper.

## Code availability

No custom code central to the conclusions of this study was generated. Image analysis was performed using ImageJ and CellProfiler workflows as described in the “Methods” section.

## References

[1] Lukashev, M. E. & Werb, Z. ECM signalling: orchestrating cell behaviour and misbehaviour. *Trends Cell Biol.* **8**, 437–441 (1998). 10.1016/s0962-8924(98)01362-2 9854310

[2] Qazi, T. H. et al. Programming hydrogels to probe spatiotemporal cell biology. *Cell Stem Cell* **29**, 678–691 (2022). 10.1016/j.stem.2022.03.013 PMC9081204 35413278

[3] Decuzzi, P. & Cook, A. B. Harnessing endogenous stimuli for responsive materials in theranostics. *ACS Nano* **15**, 2068–2098 (2021). 10.1021/acsnano.0c09115 PMC7905878 33555171

[4] Zylstra, A. & Heinemann, M. Metabolic dynamics during the cell cycle. *Curr. Opin. Syst. Biol.* **30**, 100415 (2022).

[5] Chen, J. et al. Lactate and lactylation in cancer. *Signal Transduct. Target. Ther.* **10**, 38 (2025). 10.1038/s41392-024-02082-x PMC11814237 39934144

[6] Fusi, G. et al. Autonomous soft robots empowered by chemical reaction networks. *Adv. Mater.* **35**, 2209870 (2023). 10.1002/adma.202209870 36420882

[7] Chin, S. M. et al. Covalent-supramolecular hybrid polymers as muscle-inspired anisotropic actuators. *Nat. Commun.* **9**, 2395 (2018). 10.1038/s41467-018-04800-w PMC6008453 29921928

[8] Lu, Y. et al. Visible light-responsive hydrogels for cellular dynamics and spatiotemporal viscoelastic regulation. *Nat. Commun.* **16**, 1365 (2025). 10.1038/s41467-024-54880-0 PMC11794891 39904989

[9] Voukantsis, D., Kahn, K., Hadley, M., Wilson, R. & Buffa, F. M. Modeling genotypes in their microenvironment to predict single- and multi-cellular behavior. *Gigascience* **8**, giz010 (2019). 10.1093/gigascience/giz010 PMC6423375 30715320

[10] Gallaher, J. A., Brown, J. S. & Anderson, A. R. A. The impact of proliferation-migration tradeoffs on phenotypic evolution in cancer. *Sci. Rep.* **9**, 1–10 (2019). 10.1038/s41598-019-39636-x PMC6382810 30787363

[11] Leight, J. L., Drain, A. P. & Weaver, V. M. Extracellular matrix remodeling and stiffening modulate tumor phenotype and treatment response. *Annu. Rev. Cancer Biol.* **1**, 313–334 (2017).

[12] Noe, J. T. et al. Lactate supports a metabolic-epigenetic link in macrophage polarization. *Sci. Adv.* **7**, eabi8602 (2021). 10.1126/sciadv.abi8602 PMC8589316 34767443

[13] Schulz, A. et al. Tyramine-conjugated alginate hydrogels as a platform for bioactive scaffolds. *J. Biomed. Mater. Res. A* **107**, 114–121 (2019). 10.1002/jbm.a.36538 PMC6585978 30256518

[14] Young Kim, B. et al. Dual enzyme-triggered in situ crosslinkable gelatin hydrogels for artificial cellular microenvironments. *Macromol. Biosci.* **16**, 1570–1576 (2016). 10.1002/mabi.201600312 27558086

[15] Chagri, S. et al. Intracellular formation of synthetic peptide nanostructures causes mitochondrial disruption and cell death in tumor spheroids. *Adv. Sci.* **12**, 2412606 (2025). 10.1002/advs.202412606 PMC12224938 40420395

[16] Anand, P. P. & Vardhanan, Y. S. Computational modelling of wet adhesive mussel foot proteins (Bivalvia): insights into the evolutionary convolution in diverse perspectives. *Sci. Rep.* **10**, 1–24 (2020). 10.1038/s41598-020-59169-y PMC7018726 32054875

[17] Wang, Y. et al. A phenol-amine superglue inspired by insect sclerotization process. *Adv. Mater.* **32**, 2002118 (2020). 10.1002/adma.202002118 32844497

[18] Jeong, H., Lee, J., Kim, S., Moon, H. & Hong, S. Site-specific fabrication of a melanin-like pigment through spatially confined progressive assembly on an initiator-loaded template. *Nat. Commun.* **14**, 1–11 (2023). 10.1038/s41467-023-38622-2 PMC10257687 37301846

[19] Golunova, A. et al. Direct and indirect biomimetic peptide modification of alginate: efficiency, side reactions, and cell response. *Int. J. Mol. Sci.* **22**, 5731 (2021). 10.3390/ijms22115731 PMC8198284 34072085

[20] Chen, X. et al. A carbodiimide-fueled reaction cycle that forms transient 5(4H)-oxazolones. *J. Am. Chem. Soc.* **145**, 6880–6887 (2023). 10.1021/jacs.3c00273 PMC10064336 36931284

[21] Booth, A. J. et al. Acellular normal and fibrotic human lung matrices as a culture system for in vitro investigation. *Am. J. Respir. Crit. Care Med.* **186**, 866–876 (2012). 10.1164/rccm.201204-0754OC PMC3530219 22936357

[22] Caliari, S. R. & Burdick, J. A. A practical guide to hydrogels for cell culture. *Nat. Methods* **13**, 405–414 (2016). 10.1038/nmeth.3839 PMC5800304 27123816

[23] Polio, S. R. et al. Cross-platform mechanical characterization of lung tissue. *PLoS ONE* **13**, e0204765 (2018). 10.1371/journal.pone.0204765 PMC6192579 30332434

[24] Papadakis, C. M., Košovan, P., Richtering, W. & Wöll, D. Polymers in focus: fluorescence correlation spectroscopy. *Colloid Polym. Sci.* **292**, 2399–2411 (2014).

[25] Wöll, D. Fluorescence correlation spectroscopy in polymer science. *RSC Adv* **4**, 2447–2465 (2013).

[26] Koynov, K. & Butt, H. J. Fluorescence correlation spectroscopy in colloid and interface science. *Curr. Opin. Colloid Interface Sci.* **17**, 377–387 (2012).

[27] Lehmann, S., Seiffert, S. & Richtering, W. Spatially resolved tracer diffusion in complex responsive hydrogels. *J. Am. Chem. Soc.* **134**, 15963–15969 (2012). 10.1021/ja306808j 22934572

[28] de la Cruz-López, K. G., Castro-Muñoz, L. J., Reyes-Hernández, D. O., García-Carrancá, A. & Manzo-Merino, J. Lactate in the regulation of tumor microenvironment and therapeutic approaches. *Front. Oncol.* **9**, 1143 (2019). 10.3389/fonc.2019.01143 PMC6839026 31737570

[29] Wu, H., Ying, M. & Hu, X. Lactic acidosis switches cancer cells from aerobic glycolysis back to dominant oxidative phosphorylation. *Oncotarget* **7**, 40621 (2016). 10.18632/oncotarget.9746 PMC5130031 27259254

[30] Gross, A. J. & Sizer, I. W. The oxidation of tyramine, tyrosine, and related compounds by peroxidase. *J. Biol. Chem.* **234**, 1611–1614 (1959). 13654426

[31] Gaikwad, R., Thangaraj, P. R. & Sen, A. K. Direct and rapid measurement of hydrogen peroxide in human blood using a microfluidic device. *Sci. Rep.* **11**, 2960 (2021). 10.1038/s41598-021-82623-4 PMC7858642 33536535

[32] Vilema-Enríquez, G., Arroyo, A., Grijalva, M., Amador-Zafra, R. I. & Camacho, J. Molecular and cellular effects of hydrogen peroxide on human lung cancer cells: potential therapeutic implications. *Oxid. Med. Cell. Longev.* **2016**, 1908164 (2016). 10.1155/2016/1908164 PMC4916325 27375834

[33] Chiou, S. Y., Lee, Y. S., Jeng, M. J., Tsao, P. C. & Soong, W. J. Moderate hypothermia attenuates oxidative stress injuries in alveolar epithelial A549 cells. *Exp. Lung Res.* **39**, 217–228 (2013). 10.3109/01902148.2013.792881 23647088

[34] Chu, Z. et al. Recent advances on modulation of H2O2 in tumor microenvironment for enhanced cancer therapeutic efficacy. *Coord. Chem. Rev.* **481**, 215049 (2023).

[35] Cai, G. et al. IGFBP1 sustains cell survival during spatially-confined migration and promotes tumor metastasis. *Adv. Sci.* **10**, 2206540 (2023). 10.1002/advs.202206540 PMC10375137 37296072

[36] Oh, S. H. et al. Antimetastatic activity of insulin-like growth factor binding protein-3 in lung cancer is mediated by insulin-like growth factor–independent urokinase-type plasminogen activator inhibition. *Mol. Cancer Ther.* **5**, 2685–2695 (2006). 10.1158/1535-7163.MCT-06-0142 17121915

[37] Rao, J. S. et al. Inhibition of invasion, angiogenesis, tumor growth, and metastasis by adenovirus-mediated transfer of antisense uPAR and MMP-9 in non–small cell lung cancer cells. *Mol. Cancer Ther.* **4**, 1399–1408 (2005). 10.1158/1535-7163.MCT-05-0082 PMC1343495 16170032

[38] Shenberger, J. S., Zhang, L., Powell, R. J. & Barchowsky, A. Hyperoxia enhances VEGF release from A549 cells via post-transcriptional processes. *Free Radic. Biol. Med.* **43**, 844–852 (2007). 10.1016/j.freeradbiomed.2007.05.033 PMC1959513 17664148

[39] Kubala, M. H. & DeClerck, Y. A. The plasminogen activator inhibitor-1 paradox in cancer: a mechanistic understanding. *Cancer Metastasis Rev.* **38**, 483 (2019). 10.1007/s10555-019-09806-4 PMC7001780 31734763

[40] Cui, N., Hu, M. & Khalil, R. A. Biochemical and biological attributes of matrix metalloproteinases. *Prog. Mol. Biol. Transl. Sci.* **147**, 1 (2017). 10.1016/bs.pmbts.2017.02.005 PMC5430303 28413025

[41] Cheng, Z., Ma, R., Tan, W. & Zhang, L. MiR-152 suppresses the proliferation and invasion of NSCLC cells by inhibiting FGF2. *Exp. Mol. Med.* **46**, e112 (2014). 10.1038/emm.2014.51 PMC4150934 25190353

[42] Caswell, P. T. & Zech, T. Actin-based cell protrusion in a 3D matrix. *Trends Cell Biol* **28**, 823–834 (2018). 10.1016/j.tcb.2018.06.003 PMC6158345 29970282

[43] Jiang, Y. et al. Targeting extracellular matrix stiffness and mechanotransducers to improve cancer therapy. *J. Hematol. Oncol.* **15**, 34 (2022). 10.1186/s13045-022-01252-0 PMC8943941 35331296

[44] Najafi, M., Farhood, B. & Mortezaee, K. Extracellular matrix (ECM) stiffness and degradation as cancer drivers. *J. Cell. Biochem.* **120**, 2782–2790 (2019). 10.1002/jcb.27681 30321449

[45] Sasaki, S., Du, Z., Takahashi, R., Sun, Q., Sera, T. & Kudo, S. The effect of stiff substrates on the collective migration of A549 cells. *J. Biomech. Sci. Eng.* **18**, 23–00298 (2023).

[46] Serganova, I. et al. Metabolic imaging: a link between lactate dehydrogenase A, lactate and tumor phenotype. *Clin. Cancer Res.* **17**, 6250 (2011). 10.1158/1078-0432.CCR-11-0397 PMC4217119 21844011

[47] Rigler, R. & Elson, E. S. *Fluorescence Correlation Spectroscopy* Vol. 65 (Springer, Berlin, 2001).

[48] Stejskal, E. O. & Tanner, J. E. Spin diffusion measurements: spin echoes in the presence of a time-dependent field gradient. *J. Chem. Phys.* **42**, 288–292 (1965).

[49] Jerschow, A. & Müller, N. 3D diffusion-ordered TOCSY for slowly diffusing molecules. *J. Magn. Reson. A* **123**, 222–225 (1996).

[50] Jerschow, A. & Müller, N. Suppression of convection artifacts in stimulated-echo diffusion experiments. double-stimulated-echo experiments. *J. Magn. Reson.* **125**, 372–375 (1997).

[51] Wiener, S. E. et al. Generation of heterotypic primary human spheroids from glioblastoma resections and (pre-)clinical applications. *Cells* **15**, 1021 (2026). 10.3390/cells15111021 PMC13256787 42274613

