# Supplementary material for: Cell-derived lactate triggers local hydrogelation and phenotype remodelling
Source: Nat Commun. 2026 Sep 28;17:10279. doi: 10.1038/s41467-026-77702-x (PMC13619628; doi:10.1038/s41467-026-77702-x)
Supplement: Supplementary file 1 [file 41467_2026_77702_MOESM1_ESM.pdf]

Supplementary Information

Cell-derived lactate triggers local hydrogelation and phenotype remodelling

Marius G. Braun[1], Tom C. Schneider[2,3,4], Kaloian Koynov[1], Sophie Gieß[5], Leon Driehaus-Ortiz[1], Lisa Förch[1], Maximilian Schuler[1,6], Manfred Wagner[1], Christopher V. Synatschke[1], Anne Régnier-Vigouroux[5], Laura De Laporte[2,3,4,6], David Y.W. Ng[1,*], Tanja Weil[1,6,*]

[1] Max Planck Institute for Polymer Research, 55128 Mainz, Germany
* Corresponding Author. Email: weil@mpip-mainz.mpg.de; david.ng@mpip-mainz.mpg.de

[2] DWI-Leibniz Institute for Interactive Materials, 52074 Aachen, Germany

[3] Institute of Technical and Macromolecular Chemistry (ITMC), Chair of Macromolecular Materials for Medicine, RWTH Aachen University, 52074 Aachen, Germany

[4] Institute of Applied Medical Engineering (AME), Department of Advanced Materials for Biomedicine (AMB), University Hospital RWTH Aachen, Center for Biohybrid Medical Systems (CMBS), 52074 Aachen, Germany

[5] Institute for Developmental Biology and Neurobiology, Johannes Gutenberg University, 55128 Mainz, Germany

[6] Max Planck School Matter to Life, 69120 Heidelberg, Germany

Table of Contents

Synthesis 3
Alginate functionalisation 3
Alginate characterisation 3
Supplementary Figures 6
Supplementary References 20

Synthesis

Alginate functionalisation

Alginate was functionalised with tyramine by carbodiimide-mediated condensation. Sodium alginate (Sigma-Aldrich, A1112; 1 eq.) was dissolved at 1 wt% in 100 mM 2-(N-morpholino)ethanesulfonic acid (MES) buffer (Carl Roth, 4256; pH 6.0) overnight. 1-Ethyl-3-(3-dimethylaminopropyl)carbodiimide (EDC, Carl Roth, 2156; 0.5 eq.) and N-hydroxysuccinimide (NHS, Carl Roth, 9670; 0.5 eq.) were added to the solution. Tyramine hydrochloride (Carl Roth, 5234; 0.33 eq.) was added after 20 min, and the reaction was stirred overnight at room temperature. The functionalised polymer was dialysed using a 3.5 kDa molecular-weight cut-off membrane against 100 mM NaCl solution (three exchanges) and Milli-Q water (six exchanges). The purified polymer was lyophilised, stored at −20 °C and characterised by ^{1}H NMR, ^{1}H DOSY NMR and SEC/GPC.

Alginate characterisation

The characterisation of sodium alginate and determination of the β-D-mannuronate (M) to α-L-guluronate (G) ratio were performed according to published protocols.[1] The M/G ratio was determined by ^{1}H NMR spectroscopy at 90 °C using Supplementary Equation 1 and was used to calculate the degree of substitution (DS).

$$\frac{M}{G} = \frac{I_B + I_C - I_A}{I_A} = \frac{4.67 + 1,00 - 1,82}{1.82} = 1.84 \qquad (1)$$

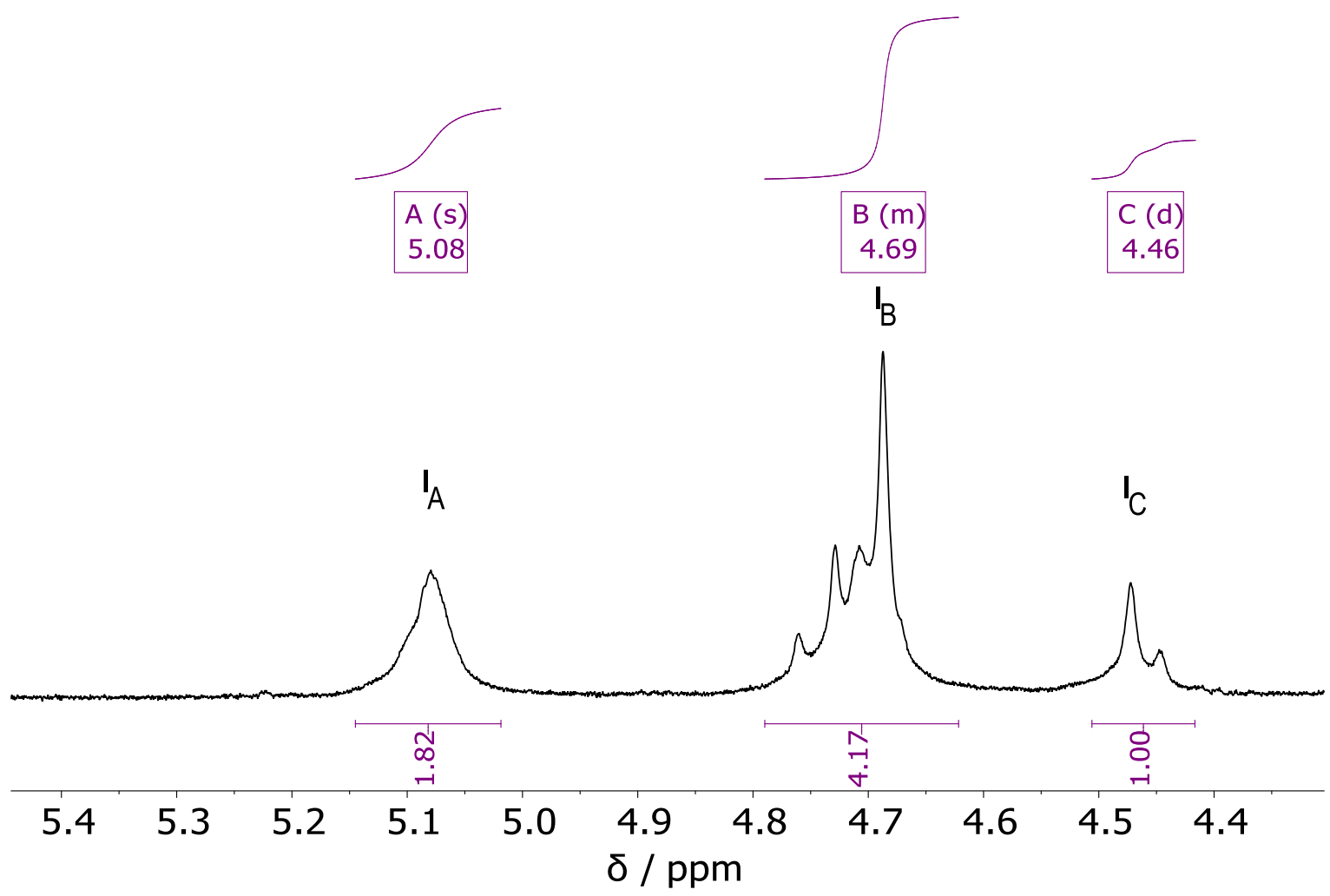

Supplementary Fig. 1 | ^{1}H NMR spectrum of sodium alginate in the spectral region from 4.3 to 5.4 ppm (700 MHz, D_2O, 363 K).

The linear copolymer alginic acid is composed of homopolymeric blocks of (1→4)-linked β-D-mannuronate (M) and α-L-guluronate (G) residues, which are covalently linked together in different sequences or blocks.[2] The monomers can appear in homopolymeric blocks of

consecutive G-residues (G-blocks), consecutive M-residues (M-blocks), or alternating M and G-residues (MG-blocks). This composition influences the polymer properties, and the M/G ratio is required to determine the degree of substitution (DS) by ^{1}H NMR peak integration.[1]

N_{arom} corresponds to the number of protons related to I_{arom}. Since the G to M ratio is 1.84, the combined intensity for all alginate units (G+M) can be expressed as 2.84 times the integral of the G unit (I_G) resulting in the functionalization degree DS presented in Supplementary Equation 2.

$$D_f = \frac{\frac{I_{arom}}{N_{arom}}}{\left(1+\frac{M}{G}\right) x I_G} = \frac{\frac{I_{arom}}{N_{arom}}}{(1+1.84) x I_G} \quad (2)$$

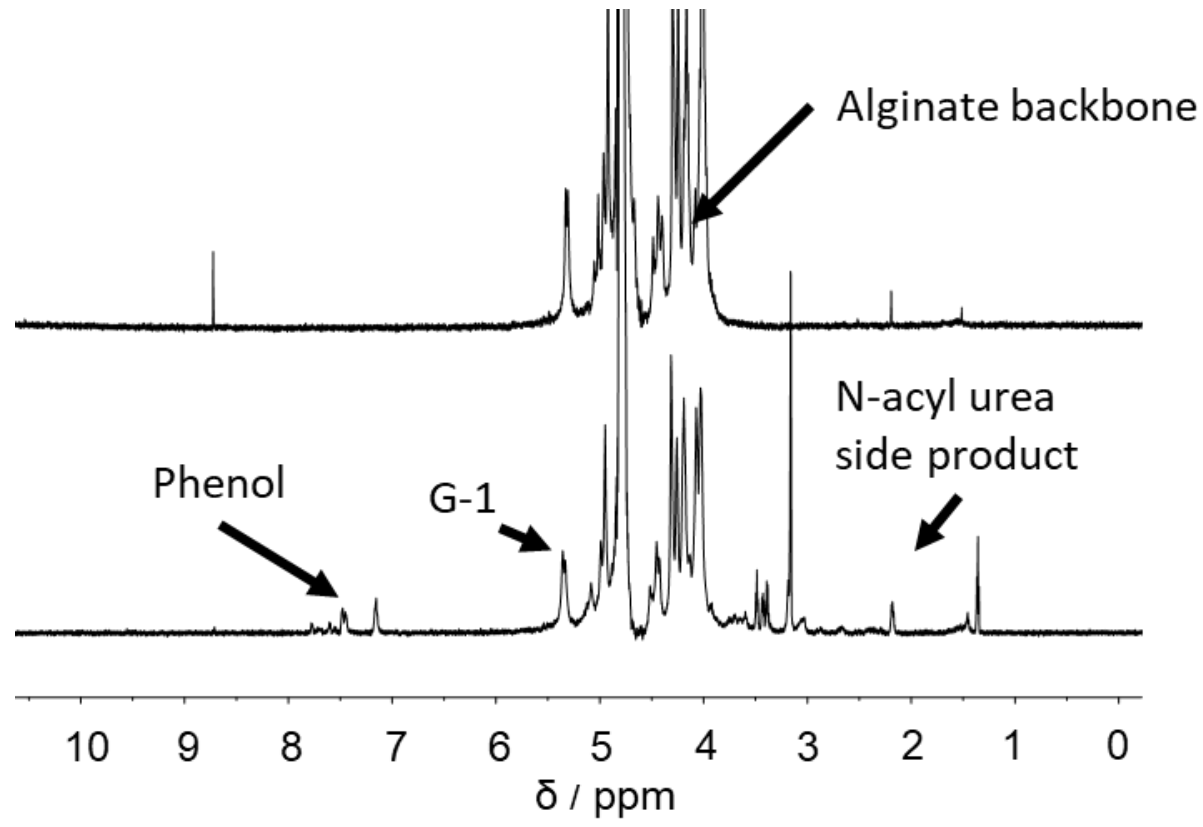

Supplementary Fig. 2 | ^{1}H NMR spectra of AlgTA and sodium alginate (0–10 ppm, 700 MHz, D_2O, 323 K).

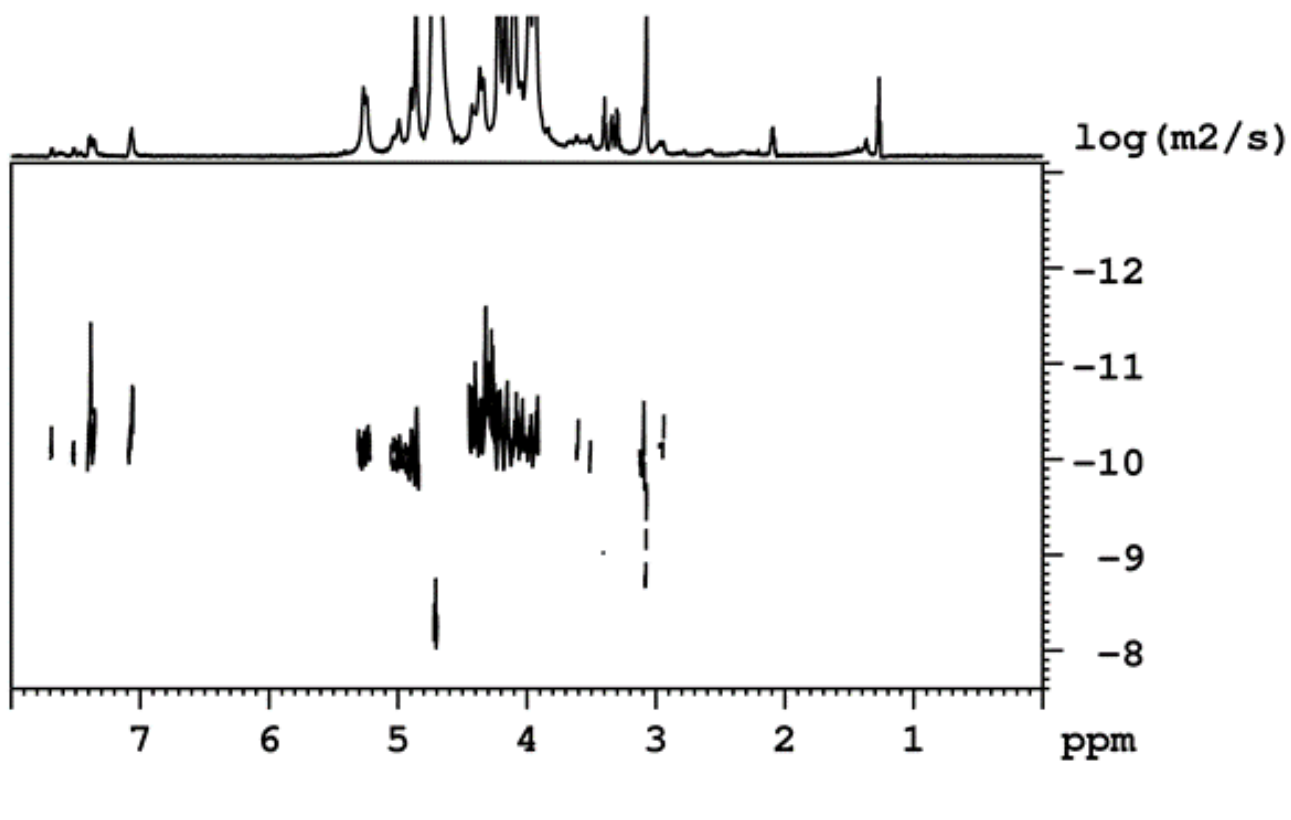

Supplementary Fig. 3 | ^{1}H DOSY NMR spectrum of AlgTA (0-8 ppm., 700 MHz, D_2O, 323 K).

To probe potential N-acylurea side product formation, a model reaction was carried out using sodium acetate as a small-molecule mimic of sodium alginate. Upon addition of EDC, the carboxylic acid is converted to an O-acylisourea intermediate. In the absence of a primary

amine, the intermediate can rearrange to an N-acylurea side product. N-acylurea formation can also occur to a minor extent in the presence of primary amines through competing side reactions of the activated intermediate. Reference spectra from the model system confirmed side-product formation, consistent with the minor resonances observed in AlgTA (Supplementary Fig. 4). ESI mass spectrometry (Supplementary Fig. 5) further supported formation of the N-acylurea rearrangement products R1 and R2 and the urea by-product **B**.

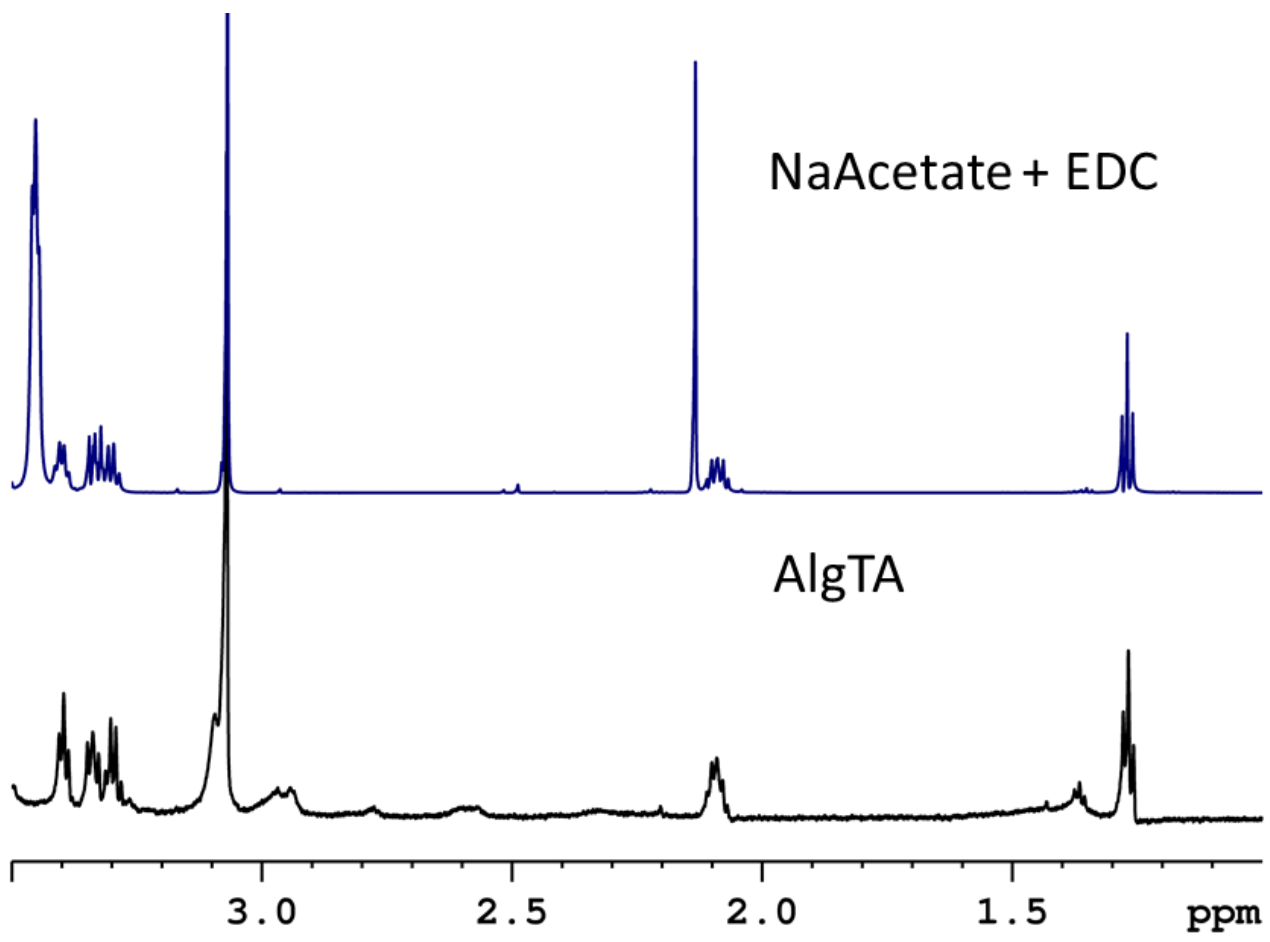

Supplementary Fig. 4 | ^{1}H NMR spectrum of the model coupling reaction using sodium acetate and EDC, compared to the purified AlgTA polymer (0-10 ppm, 700 MHz, D_2O, 323K).

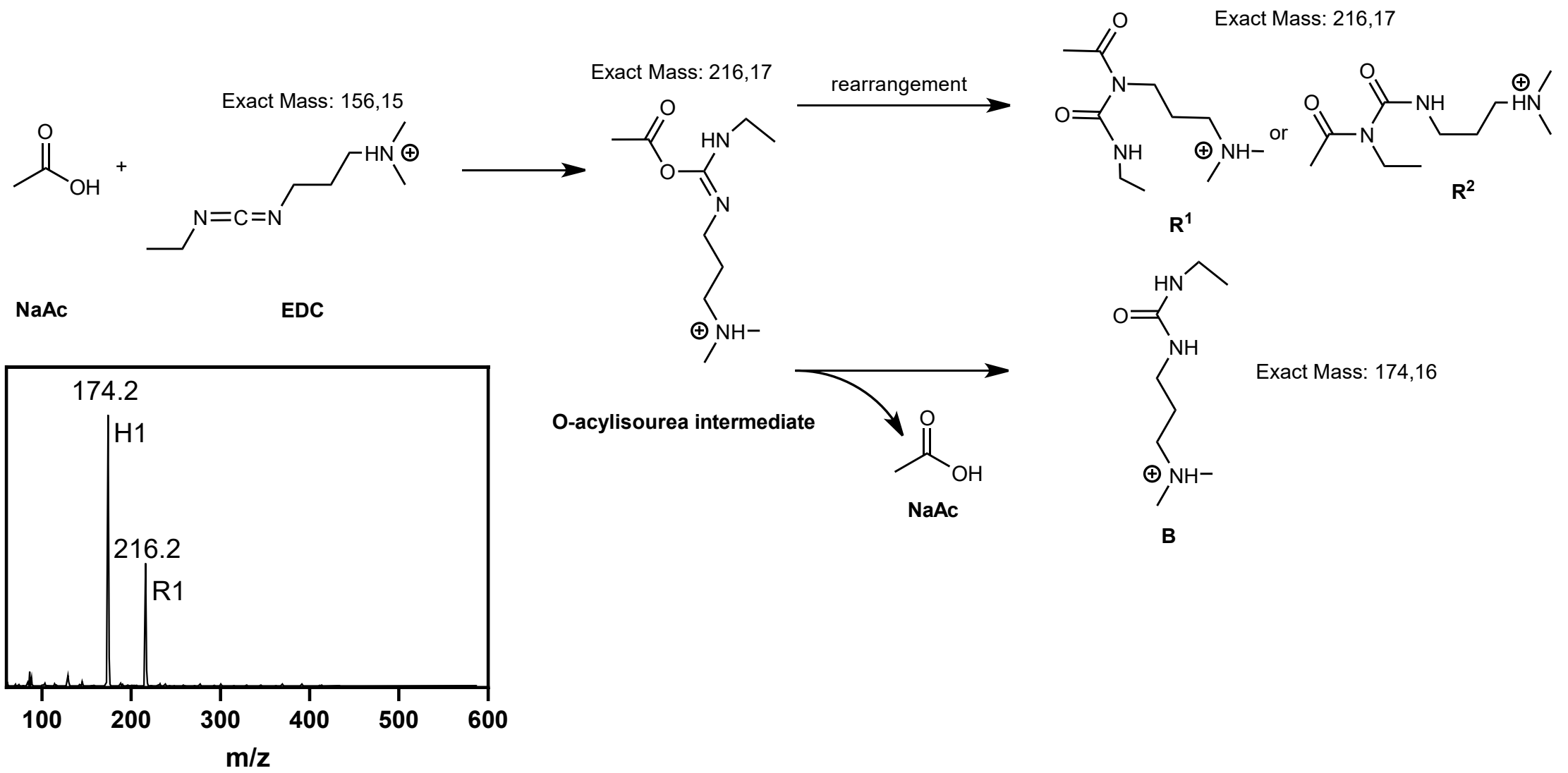

Supplementary Fig. 5 | Positive-mode ESI mass spectrum of the alginate-coupling model reaction using sodium acetate and EDC in MES buffer, recorded after 1 day. Signal **B** (m/z = 174.2) is assigned to the urea by-product, and signals **R**^{1} and **R**^{2} (m/z = 216.2) are assigned to N-acylurea rearrangement products of the O-acylisourea intermediate.

Supplementary Figures

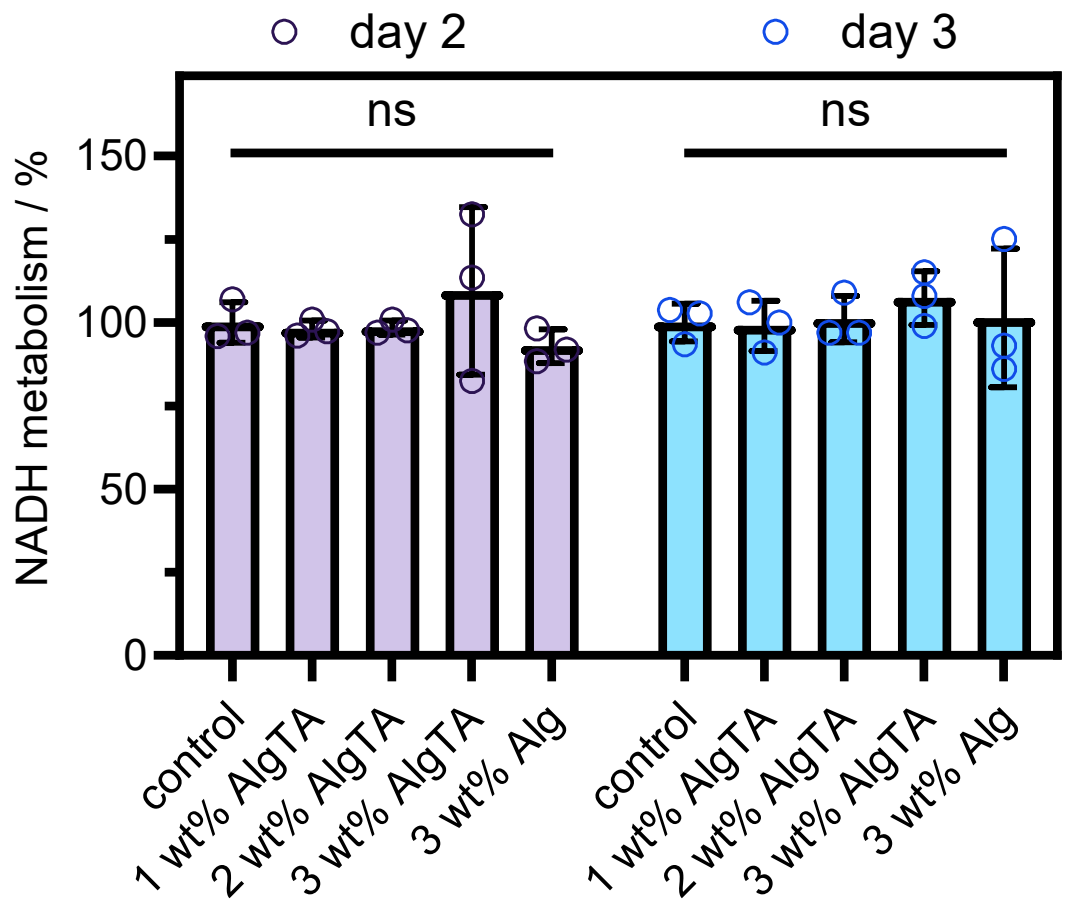

Supplementary Fig. 6 | Metabolic activity of A549 cells in AlgTA solutions. Resazurin-based NADH metabolic activity assay of spheroids cultured in AlgTA solutions (1, 2, and 3 wt%) or dialysed sodium alginate (3 wt%). Metabolic activity was normalised to control spheroids cultured in FBS supplemented medium. Data represent mean ± s.d. from triplicates. No significant reduction in NADH metabolic activity was detected under the tested material-exposure conditions (one-way ANOVA, $P > 0.05$; $n = 3$ spheroids per condition).

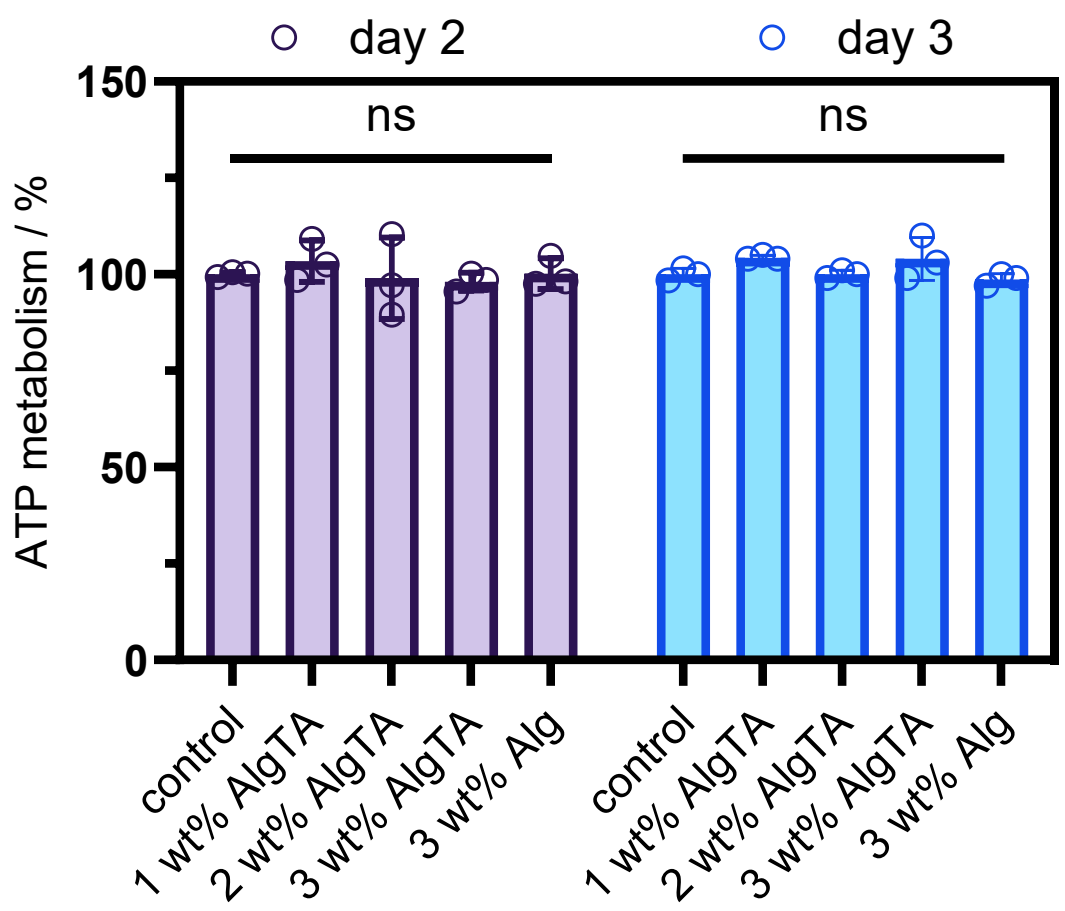

Supplementary Fig. 7 | Metabolic activity of A549 cells in AlgTA solutions. CellTiter Glo-based ATP metabolic activity assay of spheroids cultured in AlgTA solutions (1, 2, and 3 wt%) or dialysed sodium alginate (3 wt%). Metabolic activity was normalized to control spheroids cultured in FBS supplemented medium. Data represent mean ± s.d. from triplicates. No significant reduction in ATP signal was detected under the tested material-exposure conditions (one-way ANOVA, $P > 0.05$; $n = 3$ spheroids per condition).

Supplementary Table 1 | Apparent molar-mass distribution of alginate and AlgTA determined by aqueous SEC/GPC with RI detection (eluent: 0.07 M Na_2HPO_4; pullulan standards; conventional calibration; n = 4).

	Alginate	AlgTA
M_n / kDa	43.1 ± 1.1	40.6 ± 1.3
M_w / kDa	257.7 ± 7.6	155.3 ± 2.8
M_p / kDa	68.8 ± 2.9	60.0 ± 0.9
$Đ$	5.98 ± 0.16	3.83 ± 0.08

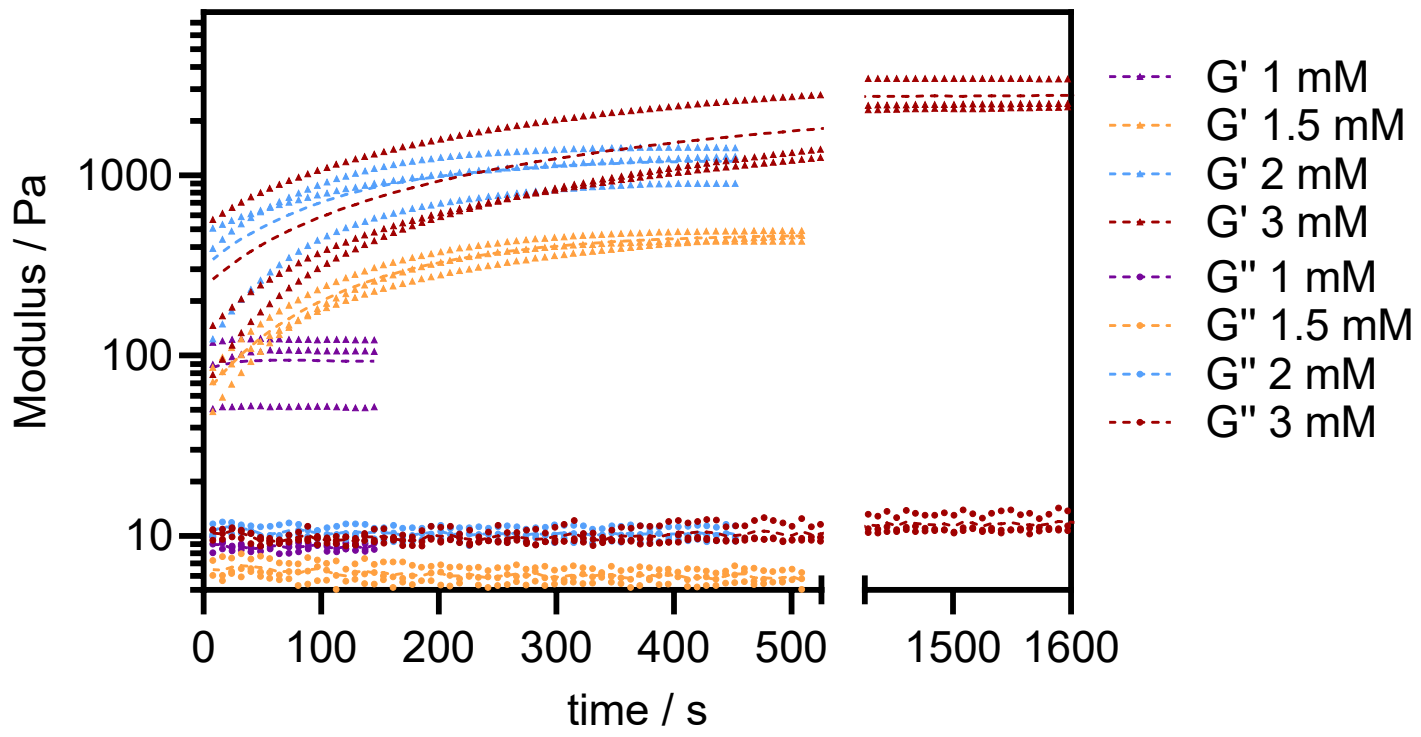

Supplementary Fig. 8 | Peroxide-triggered gelation of AlgTA in culture medium. Oscillatory time-sweep rheology of 3 wt% AlgTA in culture medium, 10 U mL^{-1} HRP and 1, 1.5, 2, or 3 mM H_2O_2. Connecting lines act as visual aids; points show individual replicates; data are mean with individual values shown (n = 3 independent gel formulations).

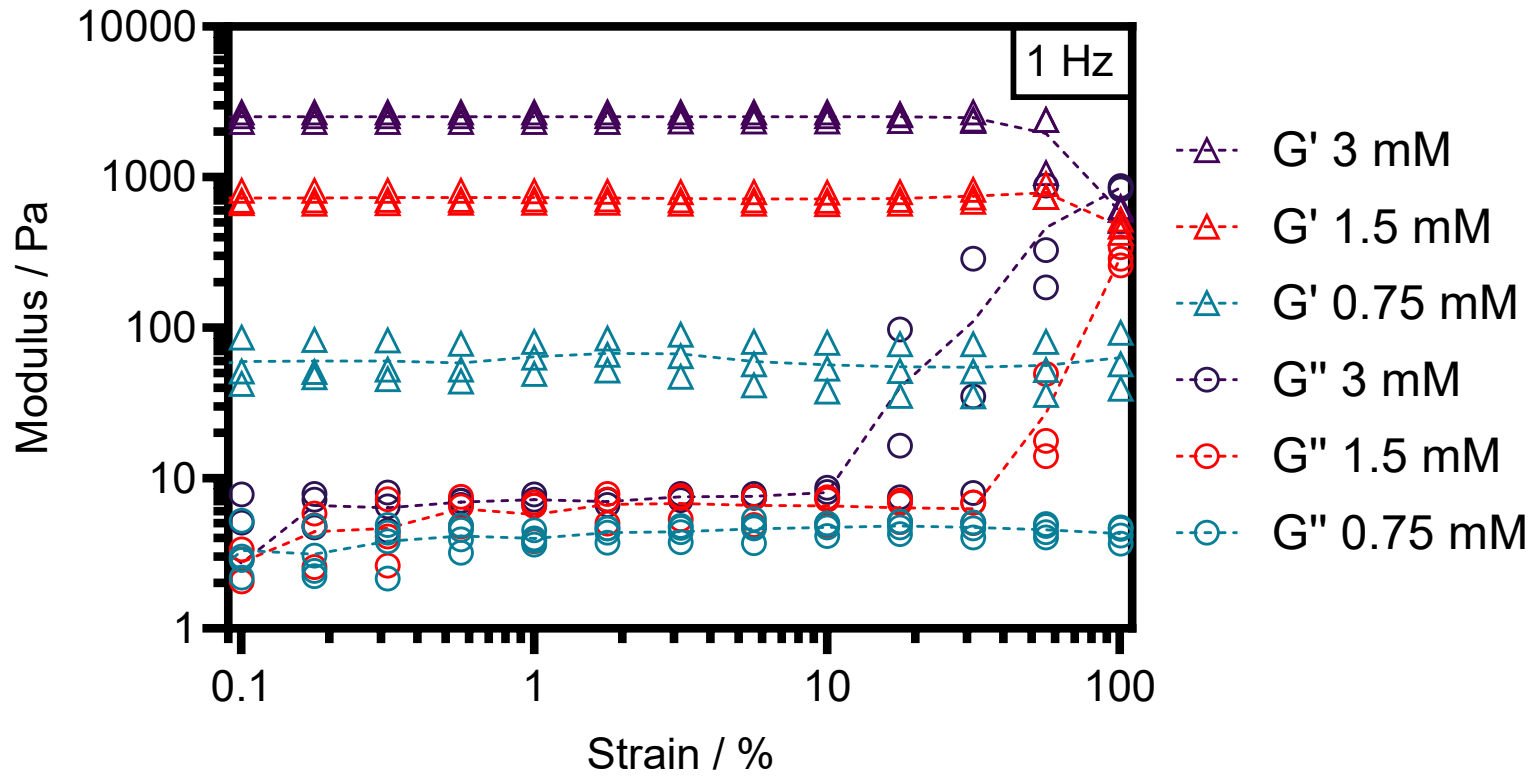

Supplementary Fig. 9 | Amplitude-sweep rheology of AlgTA in culture medium. Formulations: 3 wt% AlgTA, 10 U mL^{-1} HRP, 0.75, 1.5, or 3 mM H_2O_2. Connecting lines act as visual aids; points show individual replicates; data are mean with individual values shown (n = 3 independent gel formulations).

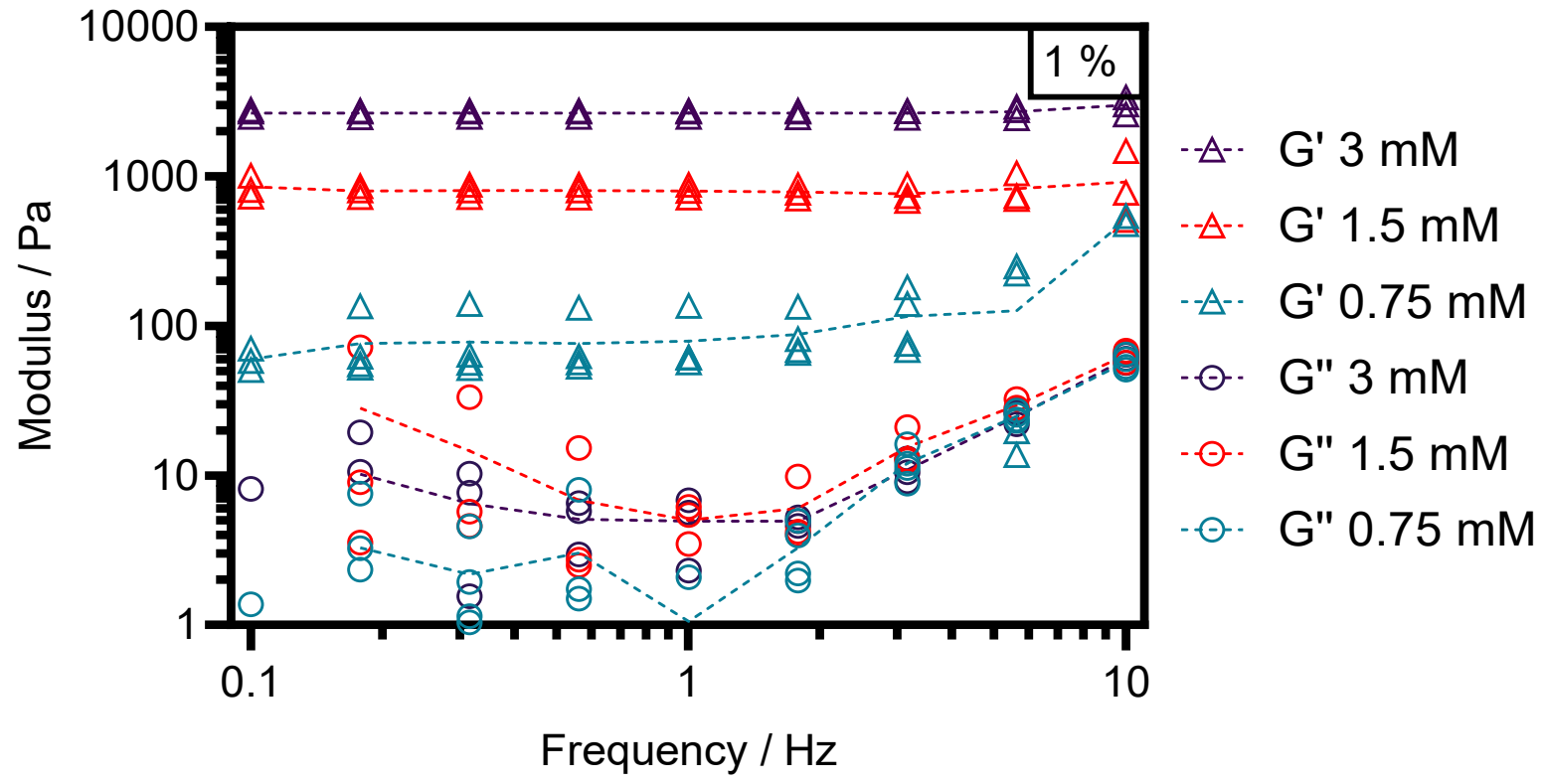

Supplementary Fig. 10 | Frequency-sweep rheology of AlgTA in culture medium. Formulations: 3 wt% AlgTA, 10 U mL^{-1} HRP, 0.75, 1.5, or 3 mM H_2O_2. Connecting lines act as visual aids; points show individual replicates; data are mean with individual values shown (*n* = 3 independent gel formulations).

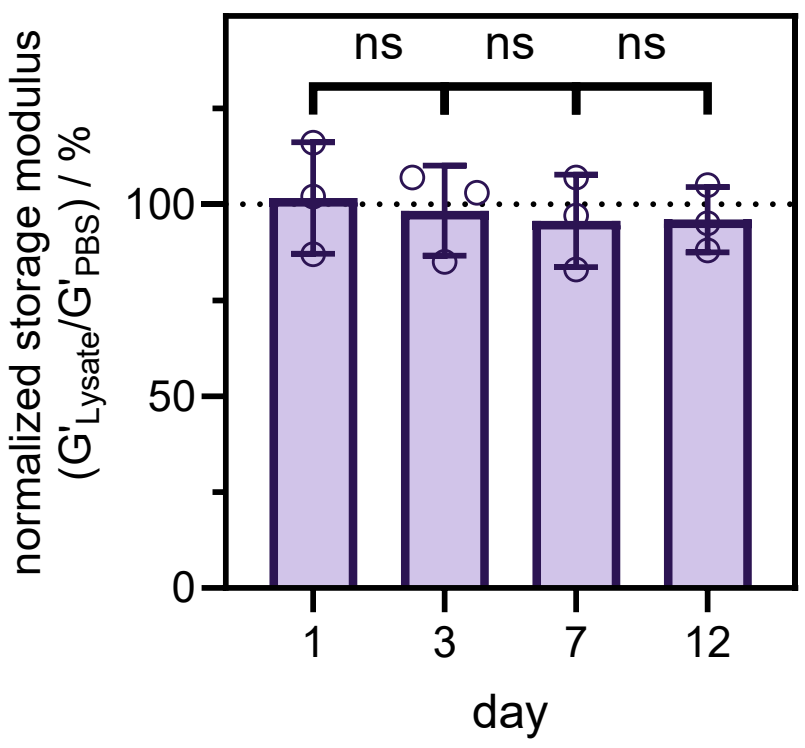

Supplementary Fig. 11 | Stability of hydrogels in the presence of cell lysate. Hydrogels were incubated with A549 cell lysate or buffer control at 37 °C for 1, 3, 7 and 12 days. Gel integrity was quantified by oscillatory rheology and normalised to the corresponding PBS buffer control. Data are mean ± s.d. (*n* = 3 independent gel formulations); statistics: unpaired two-tailed Welch's t-test. Shown P values: day 3, $P = 0.771$; day 7, $P = 0.807$; day 12, $P = 0.964$. All comparisons were not significant (ns, $P > 0.05$).

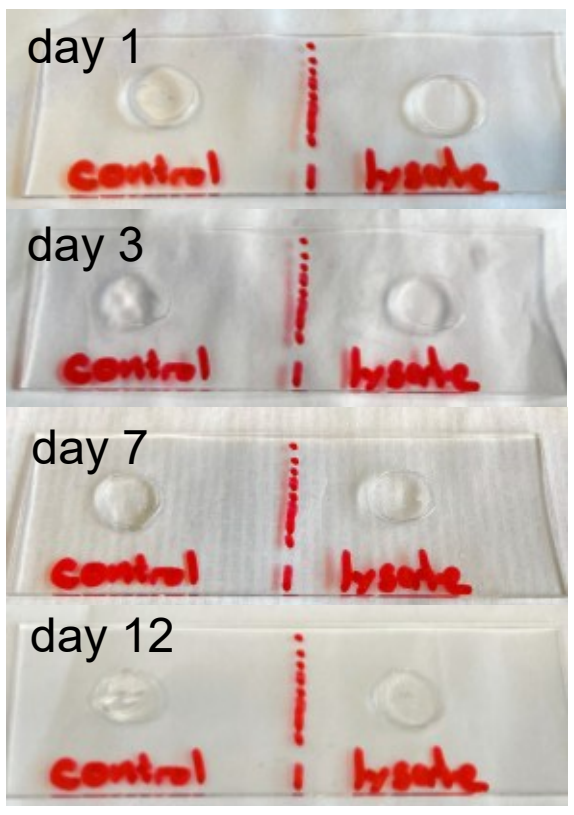

Supplementary Fig. 12 | Macroscopic stability of hydrogels in the presence of cell lysate. Hydrogels were incubated with A549 cell lysate or a buffer control at 37°C. Gels were removed from the incubation chambers and placed on glass slides for imaging after 1, 3, 7, and 12 days of incubation.

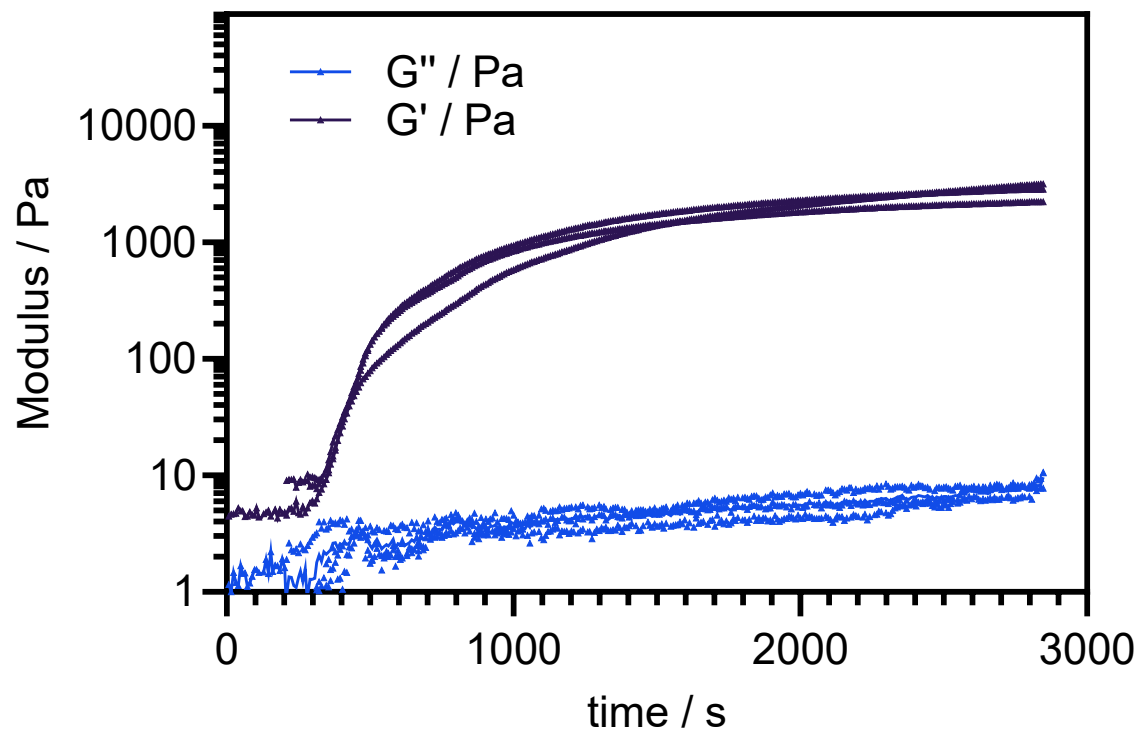

Supplementary Fig. 13 | Lactate-triggered gelation of AlgTA. Oscillatory time-sweep rheology of 3 wt% AlgTA in PBS after addition of 10 mM sodium lactate to activate the lactate oxidase–horseradish peroxidase cascade. Formulation: 3 wt% AlgTA, 10 U mL⁻¹ HRP and 1 U mL⁻¹ LOx. Measurements were performed at 1 Hz and 1% strain. Points show individual replicates; data are mean ± s.d. (*n* = 3 independent gel formulations).

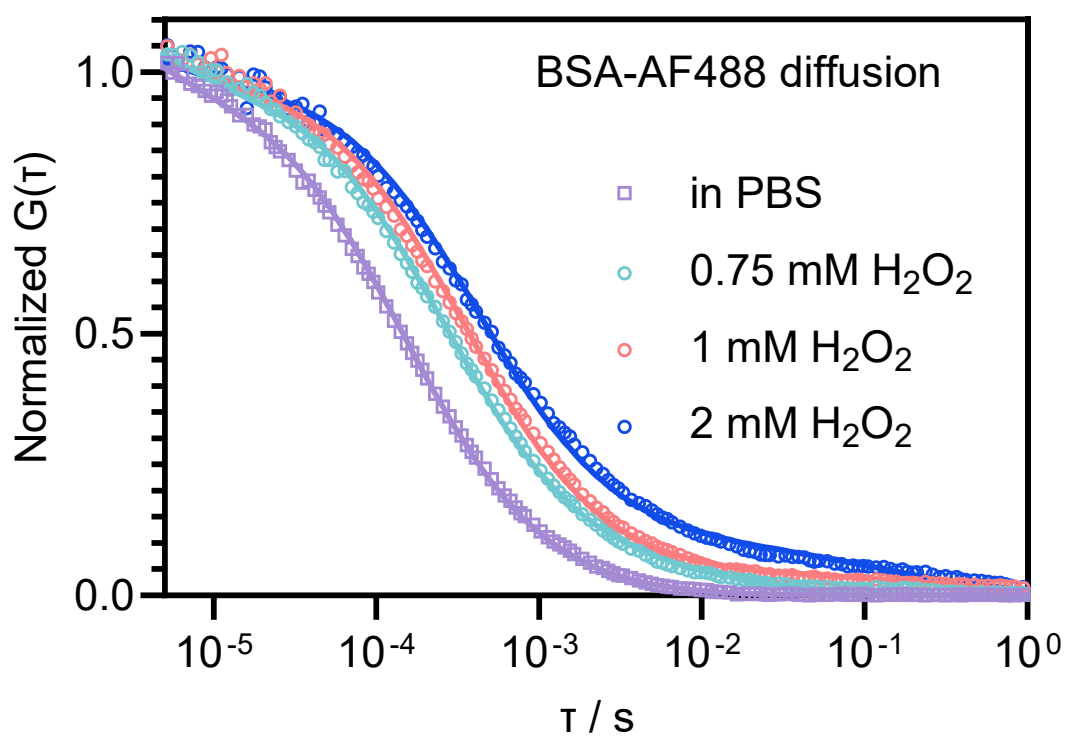

Supplementary Fig. 14 | Representative normalised experimental FCS autocorrelation curves (symbols) and the corresponding fits with equation 1 (lines), for Alexa488-BSA in PBS and AlgTA gels formed with 0.75, 1 or 2 mM H_2O_2. Fits were performed using the FCS model described in Methods.

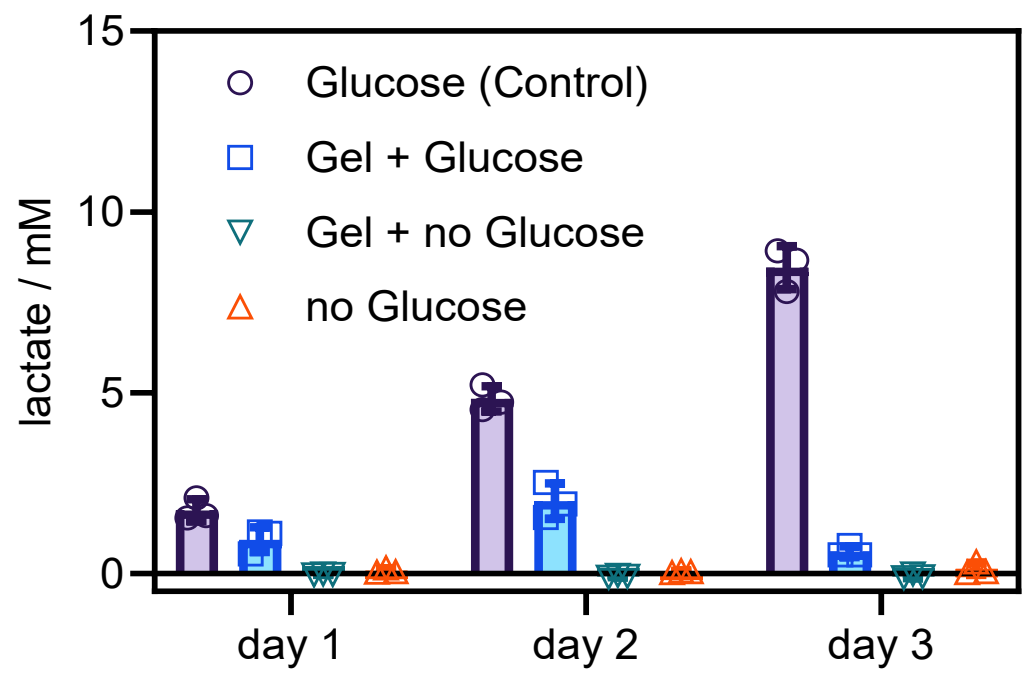

Supplementary Fig. 15 | Lactate concentrations for various conditions at 1, 2 and 3 days in the transwell assay setup. Data are mean ± s.d. (n = 3 samples per condition).

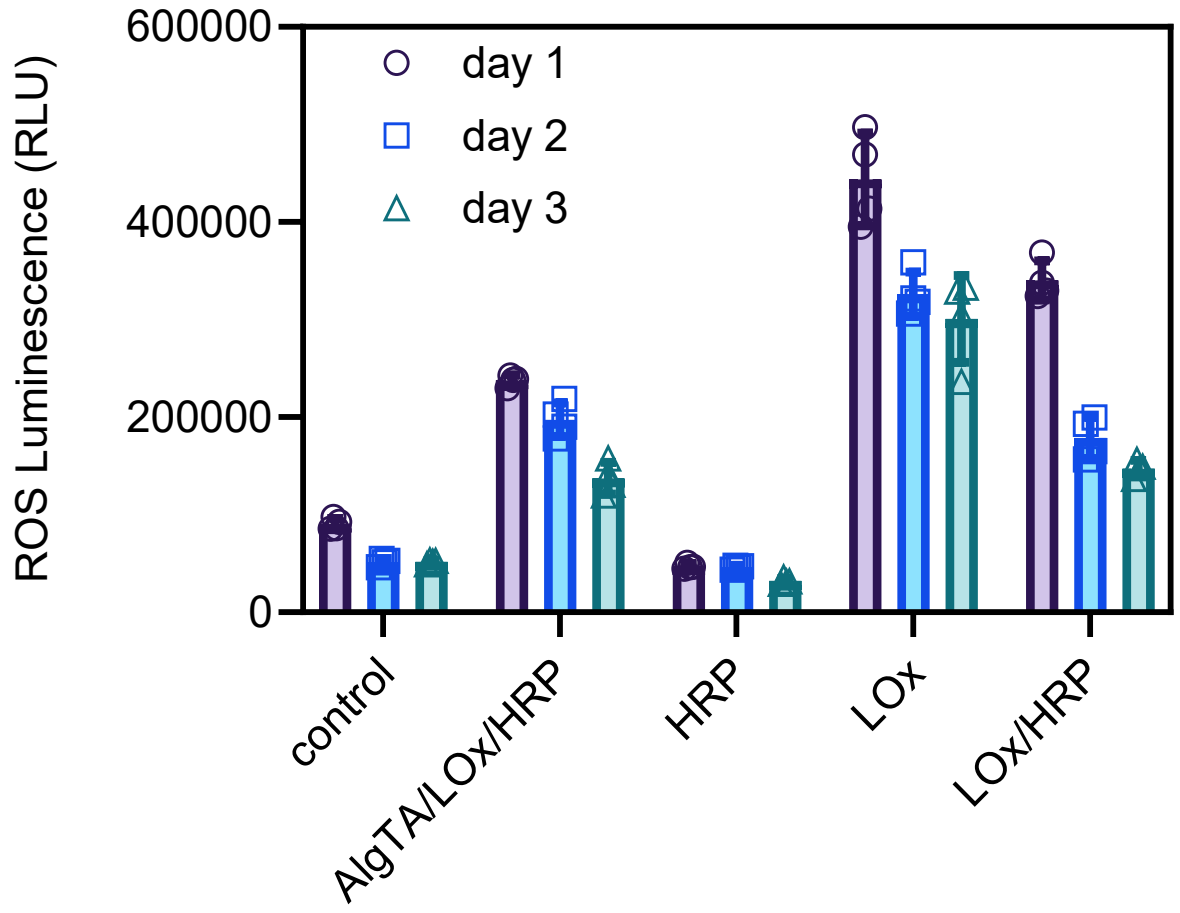

Supplementary Fig. 16 | ROS Luminescence intensity for various conditions at 1, 2 and 3 days in the transwell assay setup. Data are mean ± s.d. (n = 4 samples per condition).

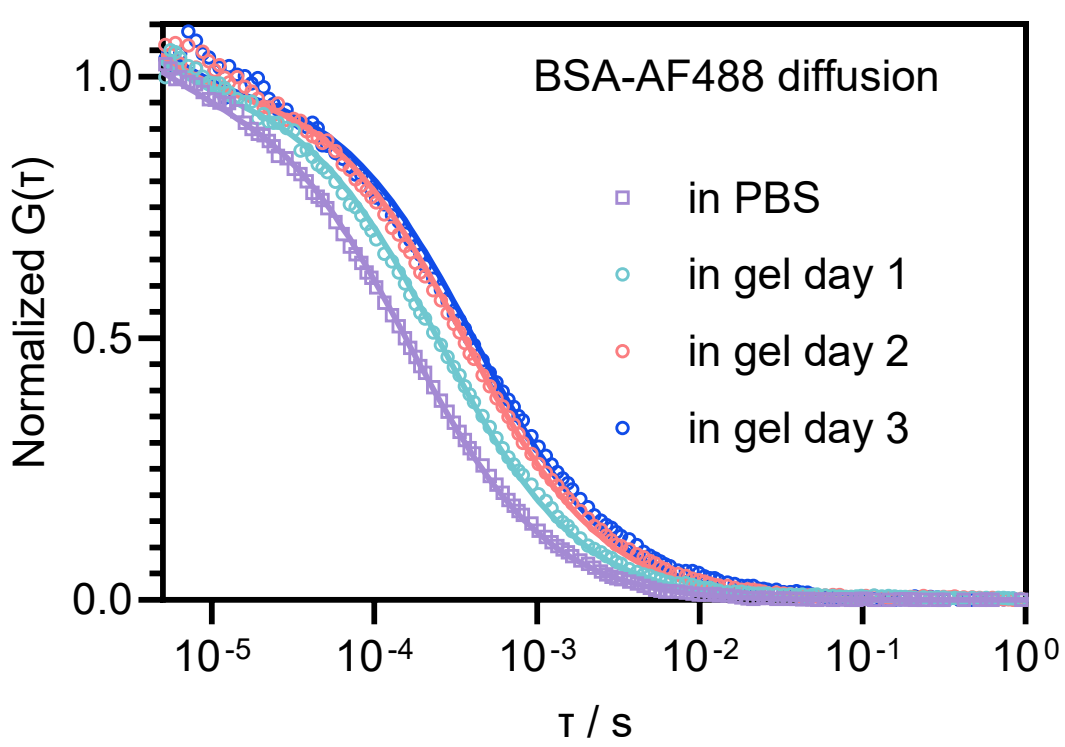

Supplementary Fig. 17 | Representative normalised experimental FCS autocorrelation curves (symbols) and the corresponding fits with equation 1 (lines), for Alexa488-BSA in PBS and AlgTA gels after 1, 2 and 3 days of cell induced gelation.

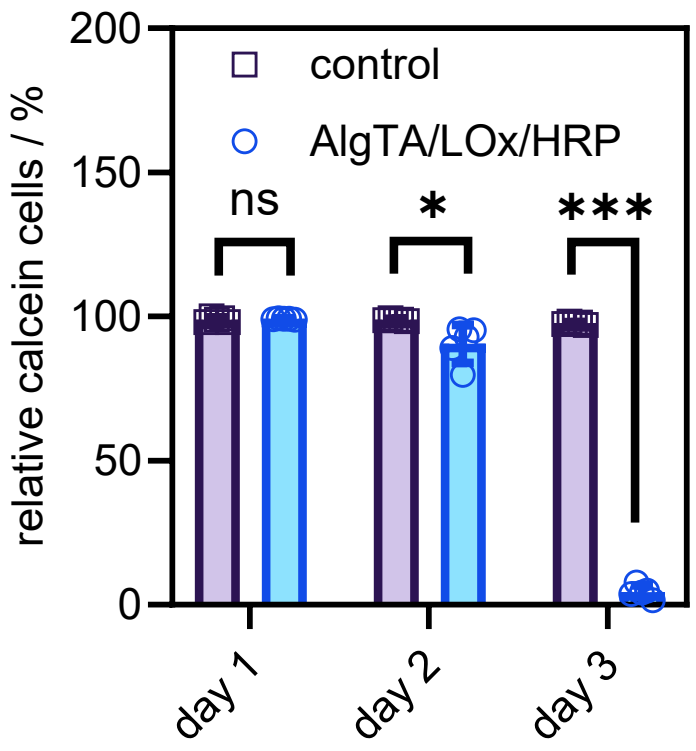

Supplementary Fig. 18 | Relative fraction of calcein positive A549 cells during transwell lactate-gated gelation. Data were quantified from five images per condition and day, with >1500 cells analysed per condition and day. Shown P values: day 1, P = 0.433; day 2, P = 0.021; day 3, P < 0.001. Data are mean ± s.d.; Statistics: unpaired two-tailed Welch's t-test; P < 0.05 (*), P < 0.01 (**), P < 0.001 (***), P > 0.05 (ns, not significant).

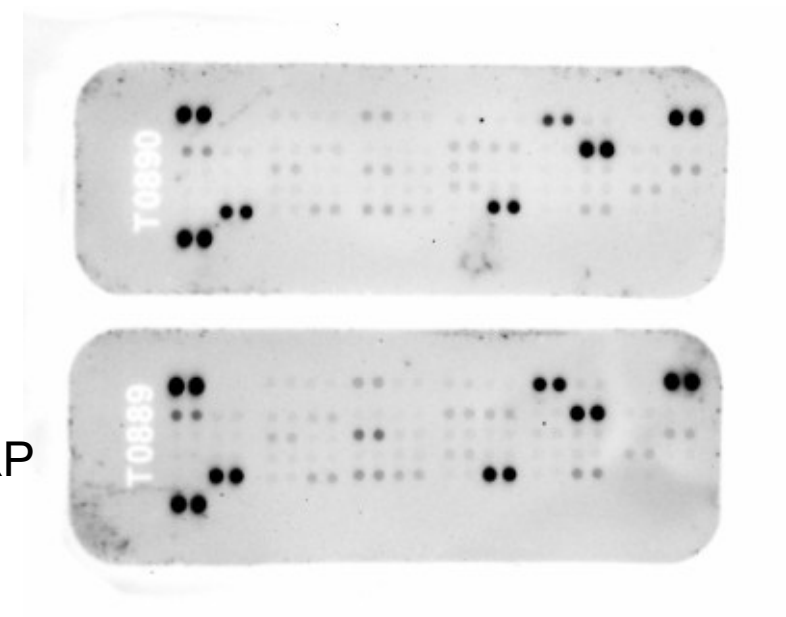

Supplementary Fig. 19 | Proteome Profiler membrane of 2D A549 cell experiments after 2 days of incubation.

	1	2	3	4	5	6	7	8	9	10	11	12	13	14	15	16	17	18	19	20	21	22	23	24
A																								
B																								
C																								
D																								
E																								

Supplementary Fig. 20 | Human Angiogenesis Proteome Profiler Array Coordinates. Corresponding target and control spots are listed in Supplementary Table 2.

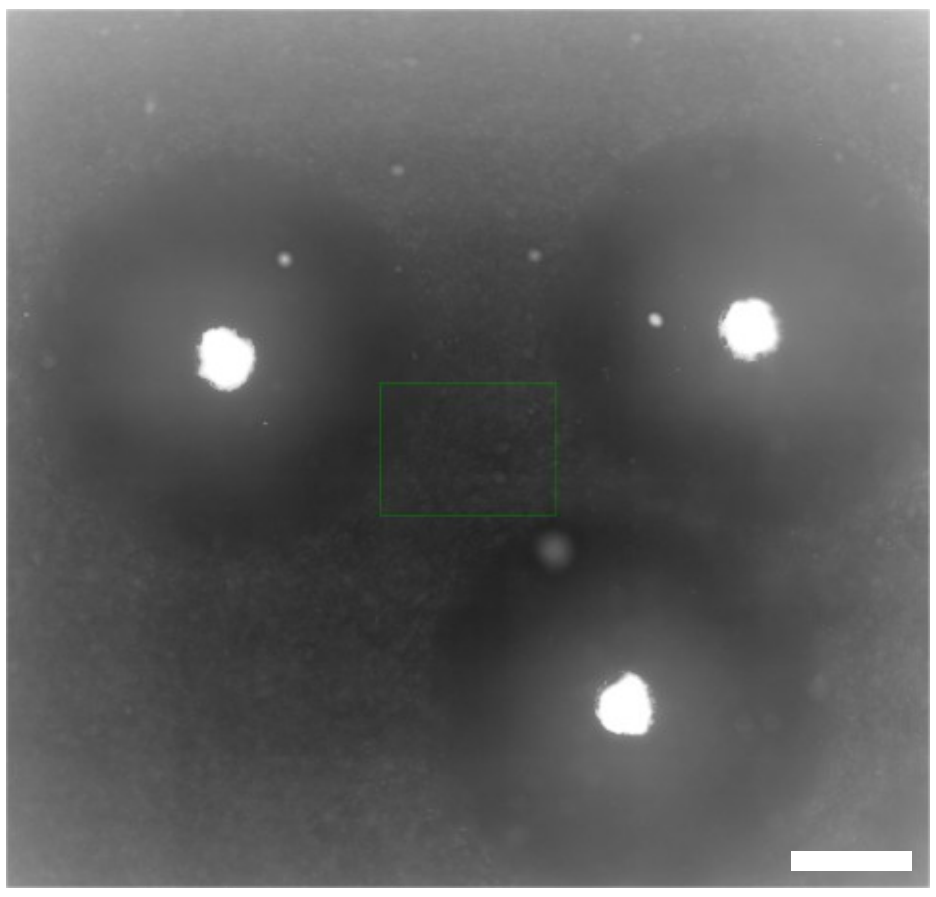

Supplementary Fig. 21 | A549 spheroids encapsulated by AlgTA/LOx/HRP after 2 days of incubation. Scale bar, 1000 µm.

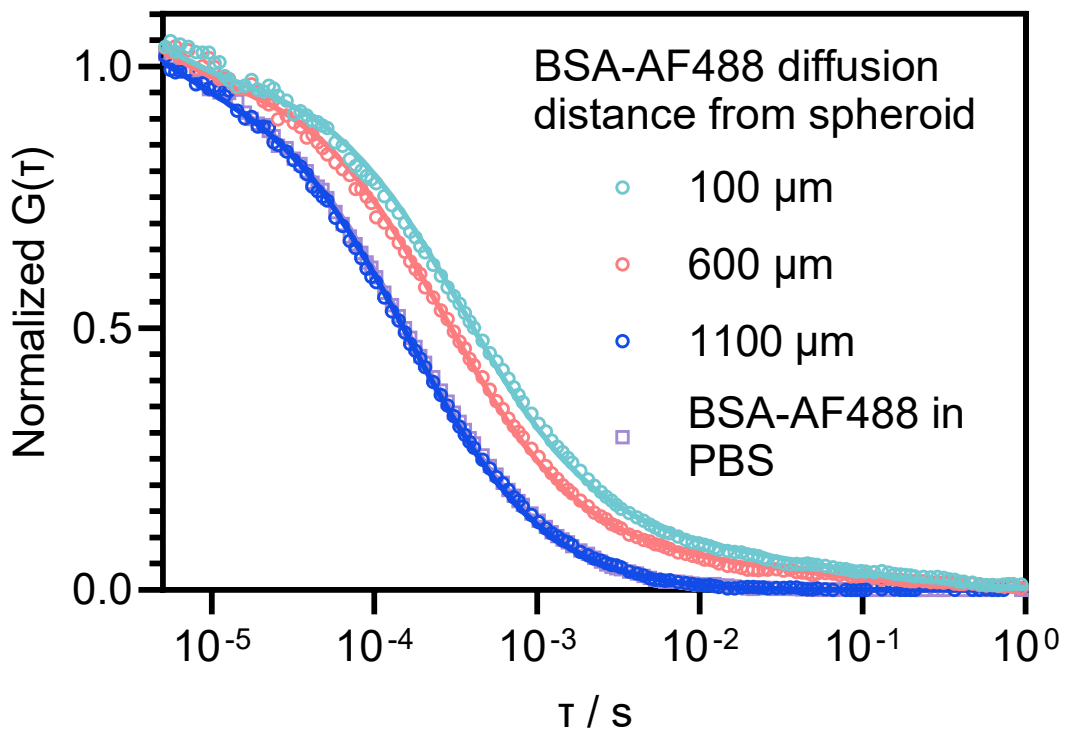

Supplementary Fig. 22 | Representative normalised experimental FCS autocorrelation curves (symbols) and the corresponding fits with equation 1 (lines), for Alexa488-BSA in PBS and spheroid induced AlgTA gels measured at 100, 600, and 1100 µm from the spheroid surface. Fits were performed using the FCS model described in Methods.

Control

AlgTA/LOx/HRP

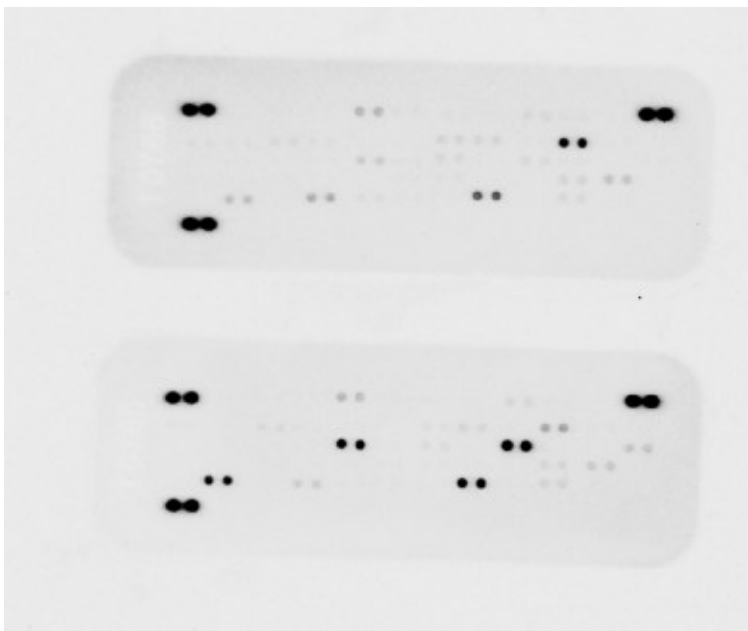

Supplementary Fig. 23 | Proteome Profiler membrane of 3D A549 spheroid experiments after 2 days of incubation.

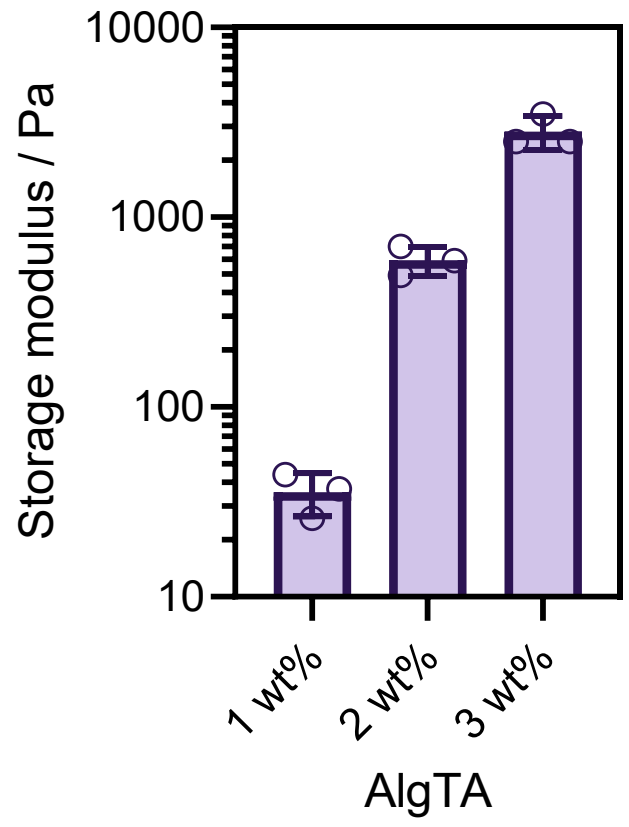

Supplementary Fig. 24 | Storage moduli of AlgTA hydrogels prepared with varied polymer concentrations. Storage modulus G′ of AlgTA hydrogels prepared with 1, 2 or 3 wt% AlgTA in culture medium. Data are mean ± s.d. (n = 3 independent gel formulations).

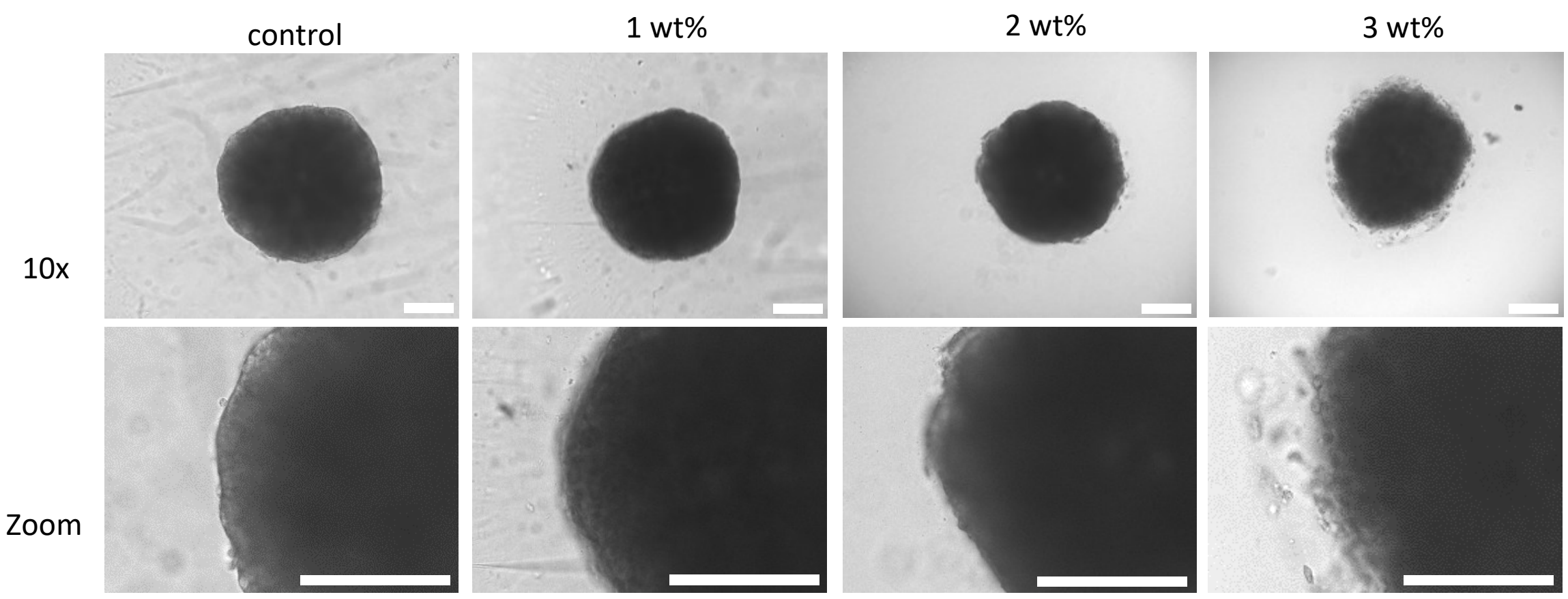

Supplementary Fig. 25 | Stiffness-dependent cell spreading of A549 spheroids. Spheroid morphology as a function of polymer concentration and matrix stiffness (1 wt%, 35 ± 9 Pa; 2 wt%, 600 ± 100 Pa; 3 wt%, 2.8 ± 0.6 kPa, Supplementary Fig. 23). Representative images of spheroids (n = 5 spheroids each) after 2 days show prominent cell protrusions in the stiffest 3 wt% matrix, whereas protrusions are absent in hydrogels with concentrations of 2 wt% or lower. Scale bar, 200 µm.

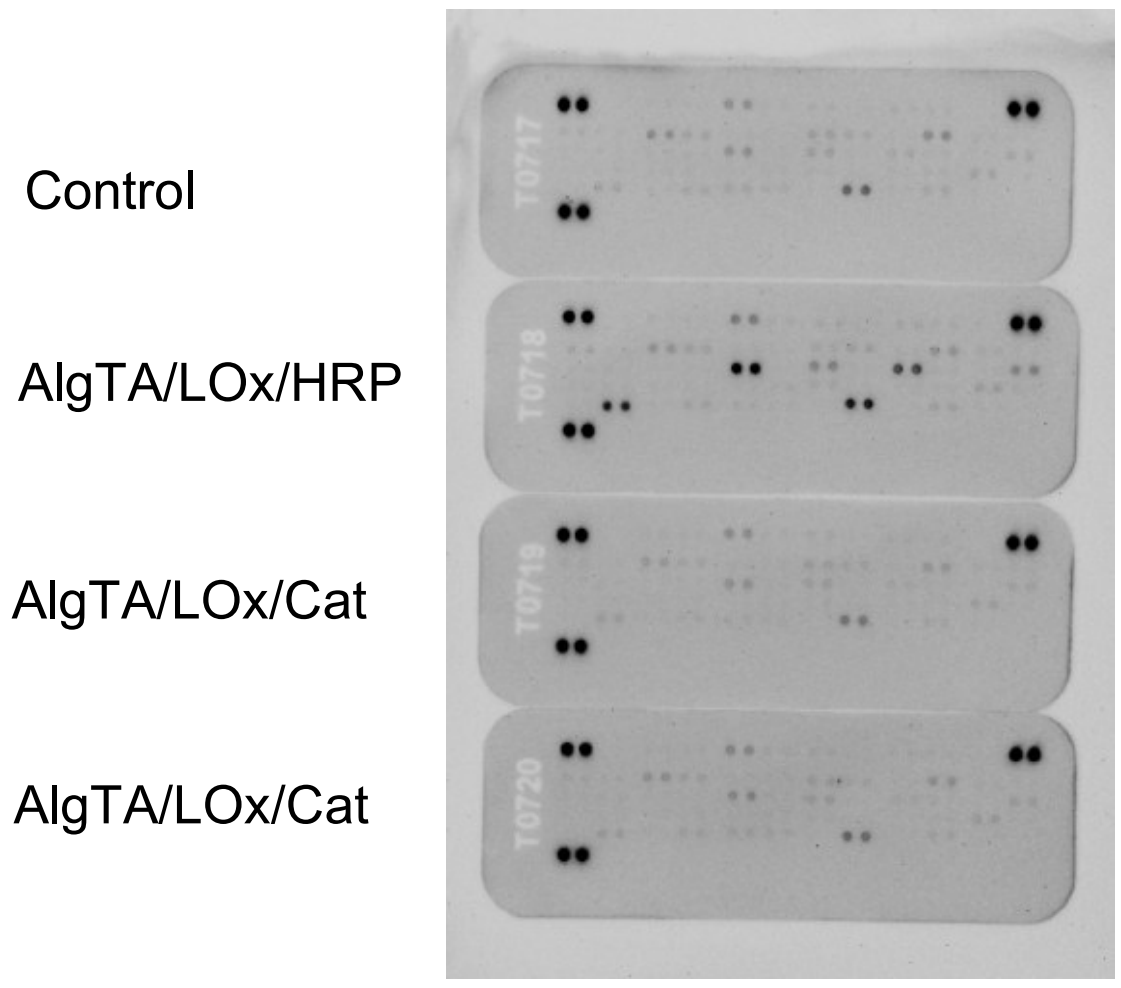

Supplementary Fig. 26 | Proteome Profiler membrane of 3D A549 spheroid experiments after 2 days of incubation with HRP replaced by catalase (Cat).

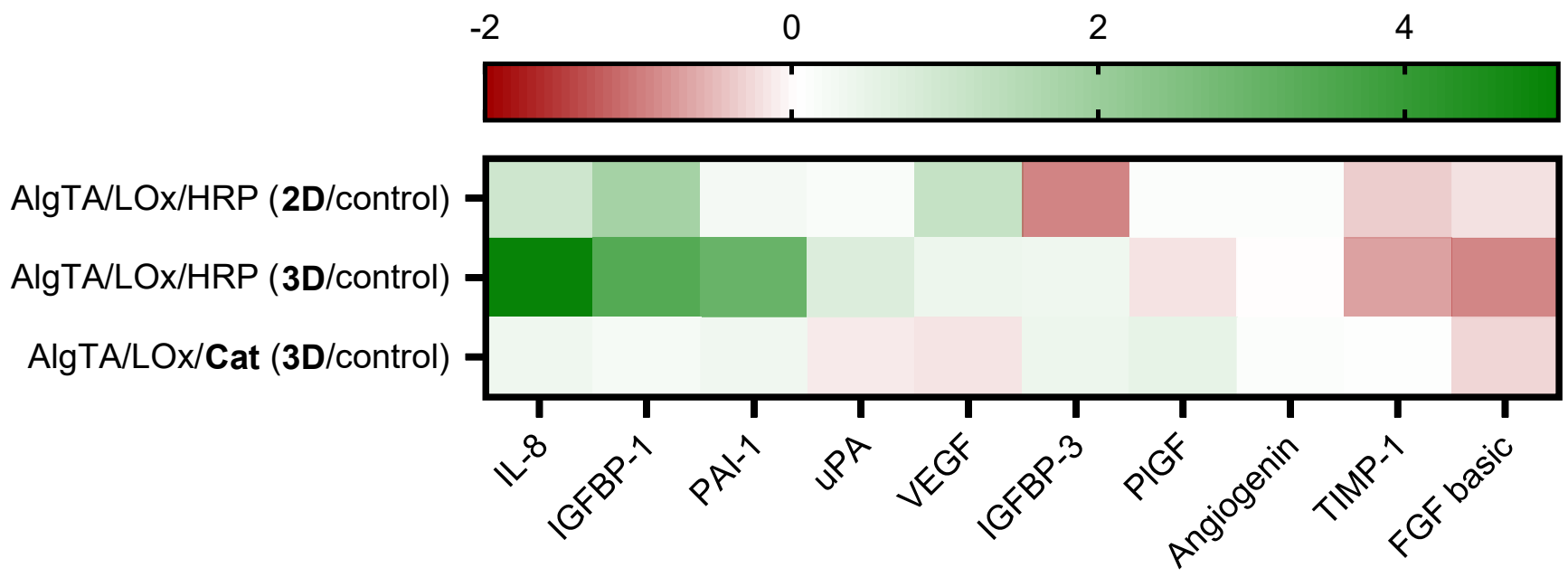

Supplementary Fig. 27 | Relative proteome profiling of selected tumour-associated proteins at day 2 versus matched controls. Replicates: AlgTA/LOx/HRP in 2D and AlgTA/LOx/HRP in 3D, n = 3 biological replicates with two on-membrane replicates; AlgTA/LOx/Cat in 3D, n = 2 biological replicates with two on-membrane replicates. Shades of red indicate decreased relative protein abundance, and shades of green indicate increased relative protein abundance. Data are presented as mean relative abundance.

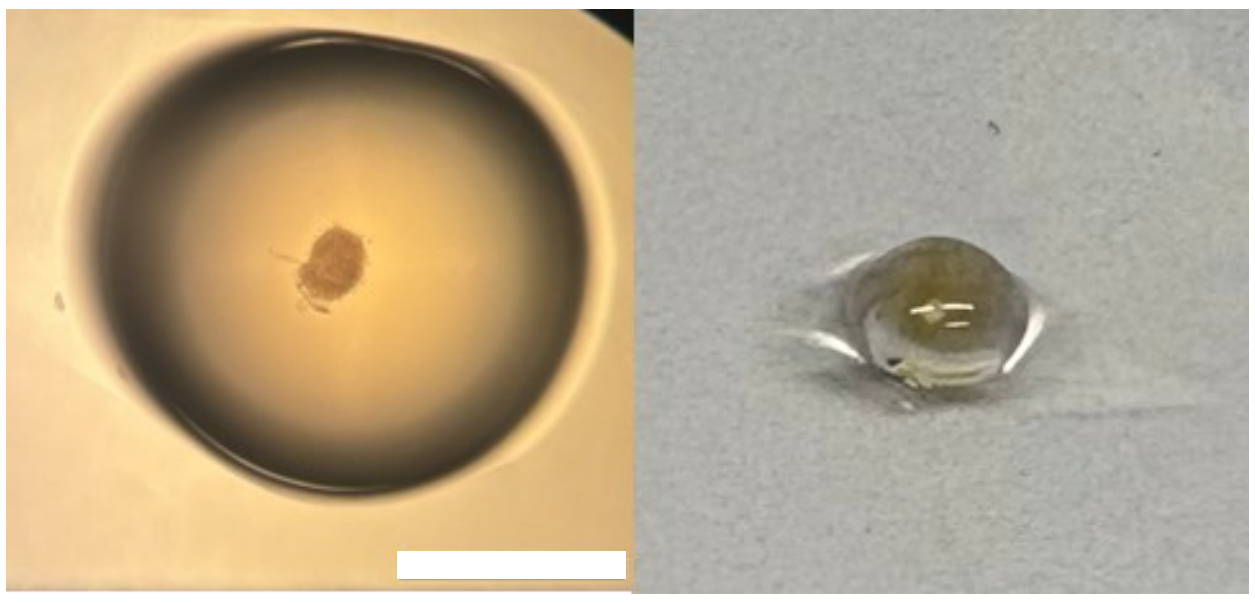

Supplementary Fig. 28 | AlgTA/LOx/HRP encapsulated 4T1 spheroids after 2 days of incubation. Scale bar, 2000 µm.

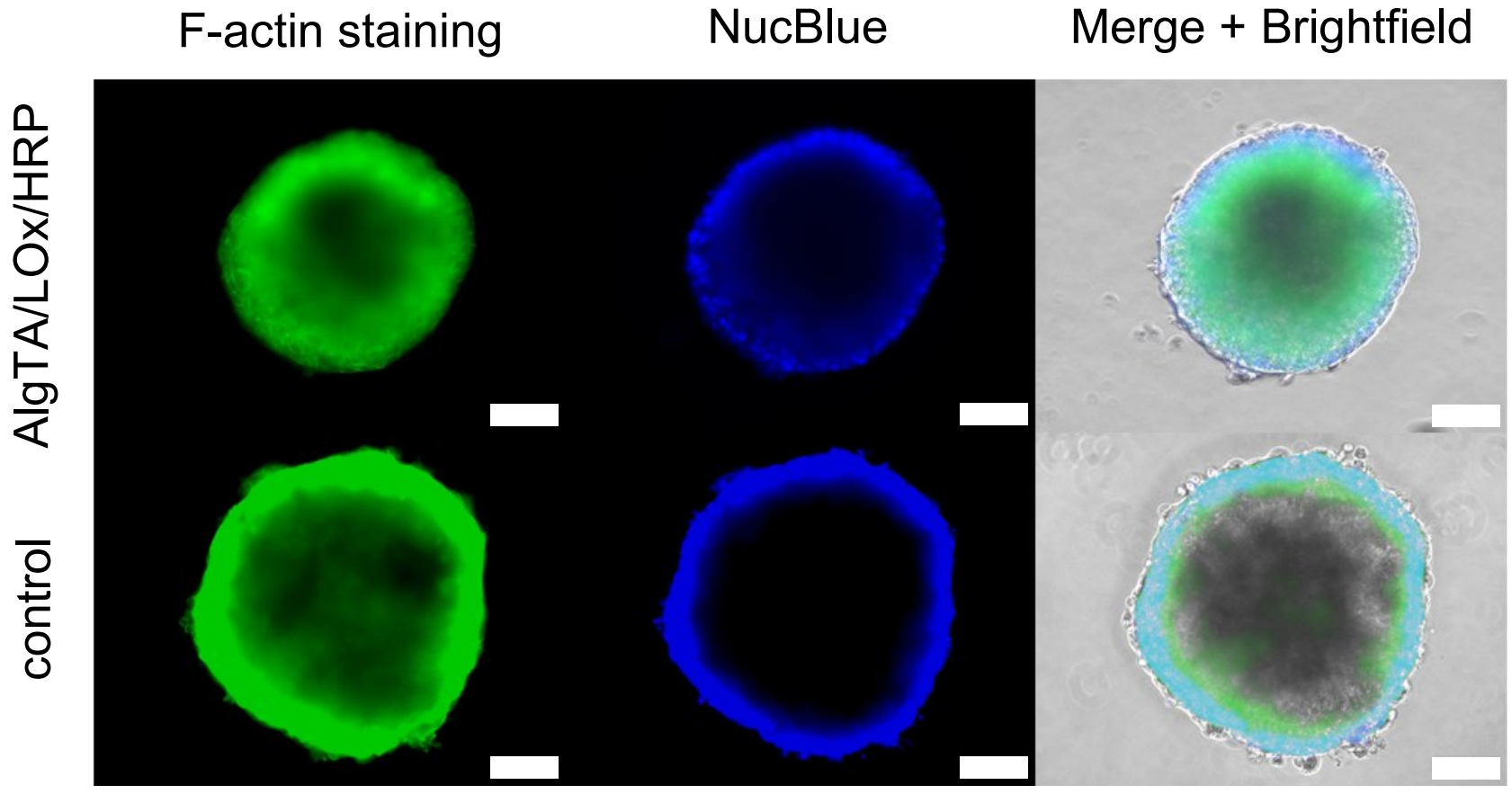

Supplementary Fig. 29 | Fluorescence micrographs of 4T1 spheroids with and without AlgTA/LOx/HRP after 2 days of incubation. Representative fluorescence micrographs from two experiments. Stains: Alexa Fluor 488 phalloidin (F-actin, green), NucBlue (nuclei, blue). Scale bars, 100 µm.

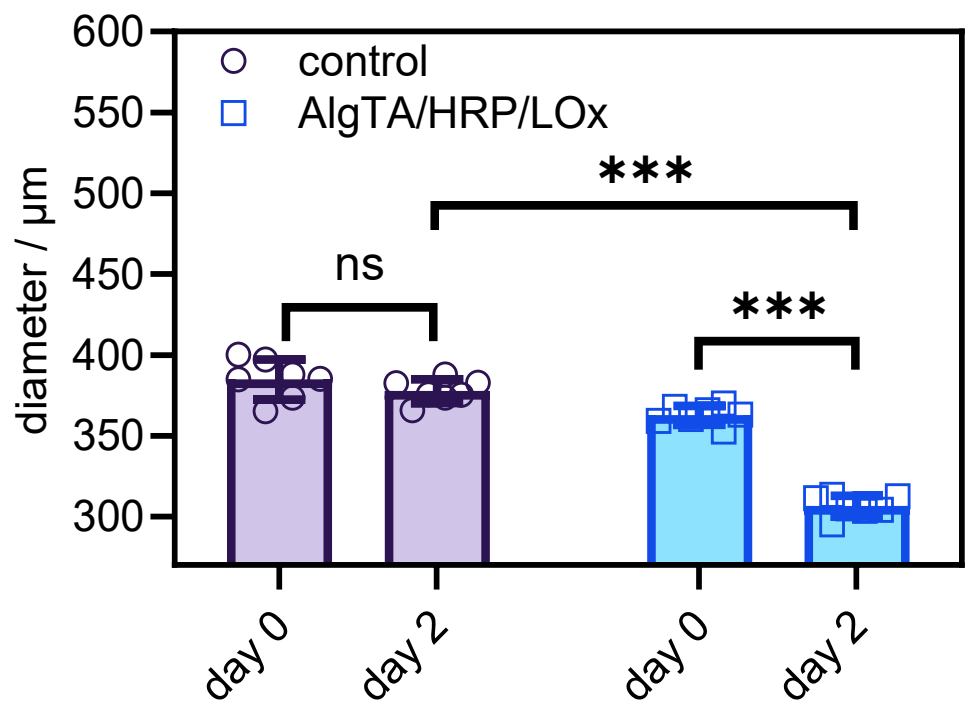

Supplementary Fig. 30 | Diameter of 4T1 spheroids with and without AlgTA/LOx/HRP after 2 days; $n = 7$ spheroids per condition. Data are mean ± s.d.; statistics: unpaired two-tailed Welch's t-test. Formulations: control, regular medium without AlgTA/LOx/HRP; AlgTA/LOx/HRP, 3 wt% AlgTA, 1 U mL^{-1} LOx and 10 U mL^{-1} HRP. Shown P values: AlgTA/LOx/HRP day 0 versus day 2, $P < 0.001$; control day 0 versus day 2, $P = 0.086$; AlgTA/LOx/HRP day 2 versus control day 0, $P < 0.001$. Statistics: unpaired two-tailed Welch's t-test; $P < 0.05$ (*), $P < 0.01$ (**), $P < 0.001$ (***), $P > 0.05$ (ns, not significant).

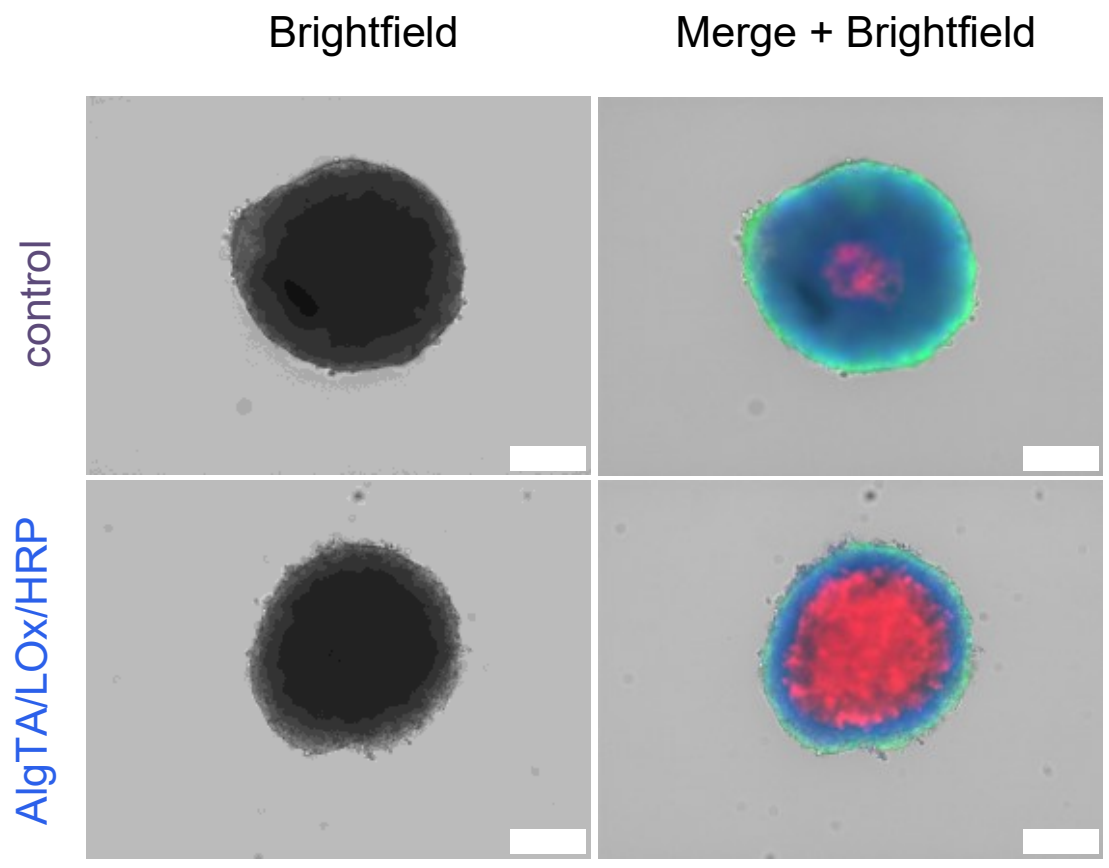

Supplementary Fig. 31 | Representative live/dead staining micrographs of lactate-gated AlgTA-encapsulated A549 spheroids after 2 days of incubation (*n* = 3 independent experiments). Formulations: control, regular medium without AlgTA/LOx/HRP; AlgTA/LOx/HRP, 3 wt% AlgTA, 1 U mL^{-1} LOx and 10 U mL^{-1} HRP. Stains: Calcein AM (live, green), NucBlue (nuclei, blue) and propidium iodide (dead, red). Scale bars, 200 μm.

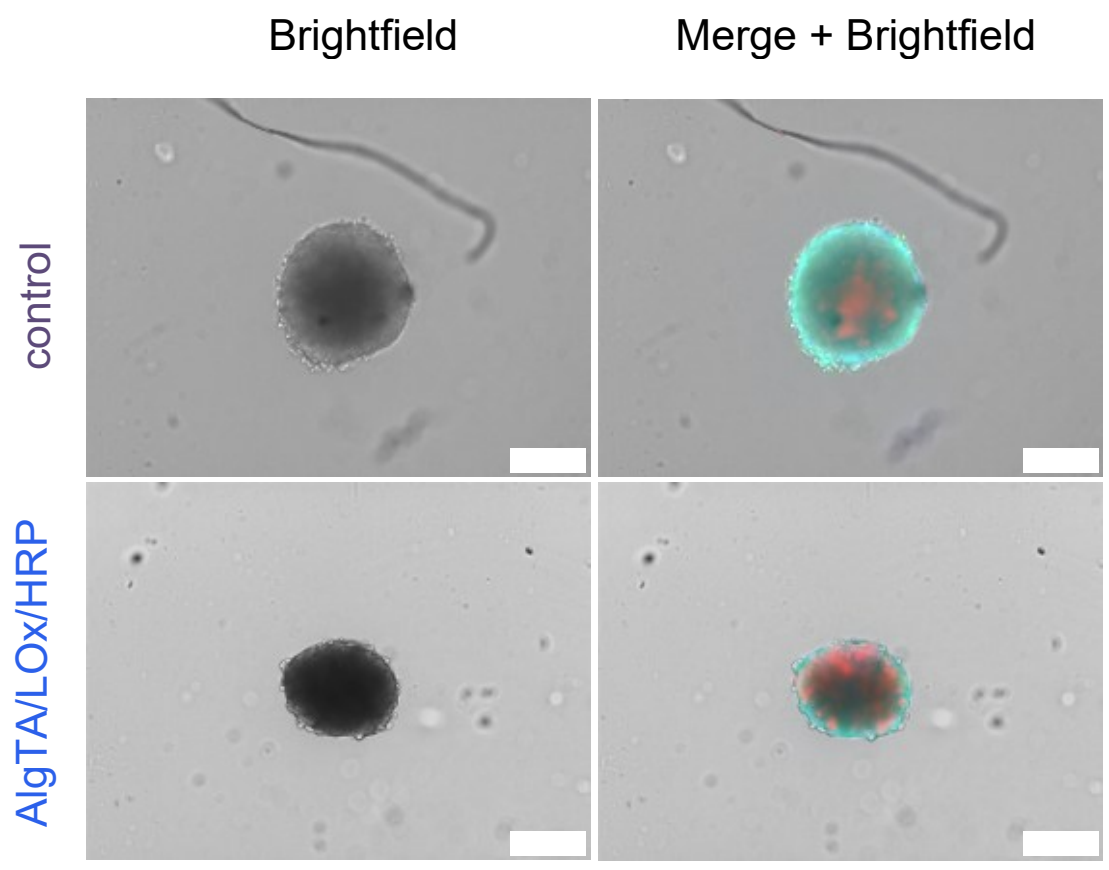

Supplementary Fig. 32 | Representative live/dead staining micrographs of lactate-gated AlgTA-encapsulated 4T1 spheroids after 2 days of incubation (*n* = 3 independent experiments). Formulations: control, regular medium without AlgTA/LOx/HRP; AlgTA/LOx/HRP, 3 wt% AlgTA, 1 U mL^{-1} LOx and 10 U mL^{-1} HRP. Stains: Calcein AM (live, green), NucBlue (nuclei, blue) and propidium iodide (dead, red). Scale bars, 200 μm.

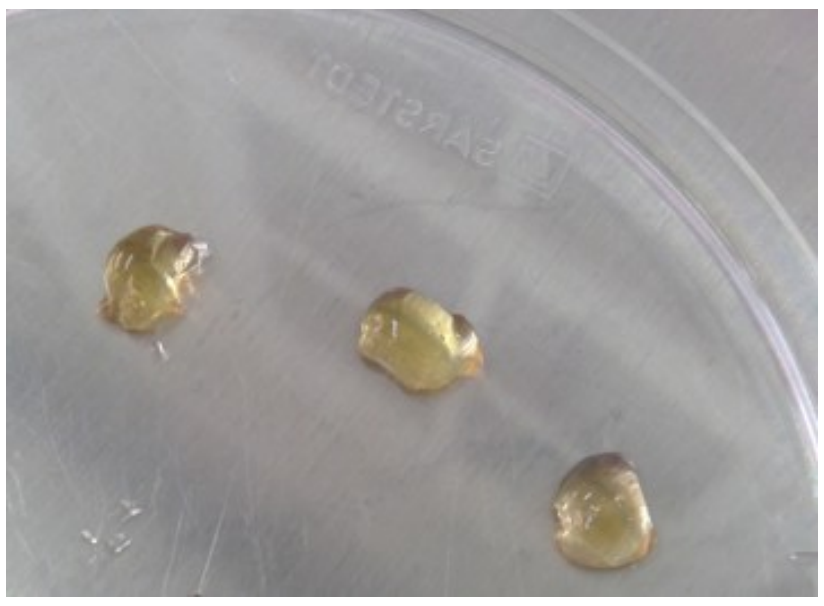

Supplementary Fig. 33 | Photograph of AlgTA/LOx/HRP-encapsulated patient-derived glioblastoma spheroids after 7 days of incubation. Formulation: AlgTA/LOx/HRP, 3 wt% AlgTA, 1 U mL^{-1} LOx and 10 U mL^{-1} HRP.

Supplementary Table 2 | Human Angiogenesis Proteome Profiler Array: target and control spots and their corresponding coordinates.

Coordinate	Target/Control	Gene ID	Alternative Nomenclature
A1, A2	Reference Spots	N/A	
A5, A6	Activin A	3624	
A7, A8	ADAMTS-1	9510	
A9, A10	Angiogenin	283	ANG
A11, A12	Angiopoietin-1	284	Ang-1
A13, A14	Angiopoietin-2	285	Ang-2
A15, A16	Angiostatin/Plasminogen	5340	
A17, A18	Amphiregulin	374	AR
A19, A20	Artemin	9048	
A23, A24	Reference Spots	N/A	
B1, B2	Coagulation Factor III	2152	TF
B3, B4	CXCL16	58191	
B5, B6	DPPIV	1803	CD26
B7, B8	EGF	1950	
B9, B10	EG-VEGF	84432	PK1
B11, B12	Endoglin	2022	CD105
B13, B14	Endostatin/Collagen XVIII	80781	
B15, B16	Endothelin-1	1906	ET-1
B17, B18	FGF acidic	2246	FGF-1
B19, B20	FGF basic	2247	FGF2
B21, B22	FGF-4	2249	
B23, B24	FGF-7	2252	KGF
C1, C2	GDNF	2668	
C3, C4	GM-CSF	1437	
C5, C6	HB-EGF	1839	
C7, C8	HGF	3082	
C9, C10	IGFBP-1	3484	
C11, C12	IGFBP-2	3485	
C13, C14	IGFBP-3	3486	
C15, C16	IL-1β	3553	IL-1F2
C17, C18	IL-8	3576	CXCL8
C19, C20	LAP (TGF-β1)	7040	
C21, C22	Leptin	3952	
C23, C24	MCP-1	6347	CCL2
D1, D2	MIP-1α	6348	CCL3
D3, D4	MMP-8	4317	
D5, D6	MMP-9	4318	
D7, D8	NRG1-β1	3084	HRG1-β1
D9, D10	Pentraxin 3 (PTX3)	5806	TSG-14
D11, D12	PD-ECGF	1890	
D13, D14	PDGF-AA	5154	
D15, D16	PDGF-AB/PDGF-BB	5155	
D17, D18	Persephin	5623	
D19, D20	Platelet Factor 4 (PF4)	5196	CXCL4
D21, D22	PlGF	5228	
D23, D24	Prolactin	5617	
E1, E2	Serpin B5	5268	Maspin

Coordinate	Target/Control	Gene ID	Alternative Nomenclature
E3, E4	Serpin E1	5054	PAI-1
E5, E6	Serpin F1	5176	PEDF
E7, E8	TIMP-1	7076	
E9, E10	TIMP-4	7079	
E11, E12	Thrombospondin-1	7057	TSP-1
E13, E14	Thrombospondin-2	7058	TSP-2
E15, E16	uPA	5328	
E17, E18	Vasohibin	22846	
E19, E20	VEGF	7422	
E21, E22	VEGF-C	7424	
F1, F2	Reference Spots	N/A	
F23, F24	Negative Control	N/A	Control (-)

Supplementary References

1. Jensen, H. M., Larsen, F. H. & Engelsen, S. B. Characterization of alginates by nuclear magnetic resonance (NMR) and vibrational spectroscopy (IR, NIR, Raman) in combination with chemometrics. *Methods in Molecular Biology* **1308**, 347–363 (2015).
2. Hecht, H. & Srebnik, S. Structural Characterization of Sodium Alginate and Calcium Alginate. *Biomacromolecules* **17**, 2160–2167 (2016).
