# Supplementary material for: Cell-derived lactate triggers local hydrogelation and phenotype remodelling
Source: Nat Commun. 2026 Sep 28;17:10279. doi: 10.1038/s41467-026-77702-x (PMC13619628; doi:10.1038/s41467-026-77702-x)
Supplement: Supplementary file 2 [file 41467_2026_77702_MOESM2_ESM.pdf]

nature portfolio

Corresponding author(s): Tanja Weil, David Y. W. Ng

Last updated by author(s): Aug 28, 2026

Reporting Summary

Nature Portfolio wishes to improve the reproducibility of the work that we publish. This form provides structure for consistency and transparency in reporting. For further information on Nature Portfolio policies, see our Editorial Policies and the Editorial Policy Checklist.

Statistics

For all statistical analyses, confirm that the following items are present in the figure legend, table legend, main text, or Methods section.

n/a	Confirmed	
☐	☒	The exact sample size (*n*) for each experimental group/condition, given as a discrete number and unit of measurement
☐	☒	A statement on whether measurements were taken from distinct samples or whether the same sample was measured repeatedly
☐	☒	The statistical test(s) used AND whether they are one- or two-sided<br>*Only common tests should be described solely by name; describe more complex techniques in the Methods section.*
☒	☐	A description of all covariates tested
☒	☐	A description of any assumptions or corrections, such as tests of normality and adjustment for multiple comparisons
☐	☒	A full description of the statistical parameters including central tendency (e.g. means) or other basic estimates (e.g. regression coefficient) AND variation (e.g. standard deviation) or associated estimates of uncertainty (e.g. confidence intervals)
☐	☒	For null hypothesis testing, the test statistic (e.g. *F*, *t*, *r*) with confidence intervals, effect sizes, degrees of freedom and *P* value noted<br>*Give P values as exact values whenever suitable.*
☒	☐	For Bayesian analysis, information on the choice of priors and Markov chain Monte Carlo settings
☒	☐	For hierarchical and complex designs, identification of the appropriate level for tests and full reporting of outcomes
☒	☐	Estimates of effect sizes (e.g. Cohen's *d*, Pearson's *r*), indicating how they were calculated

Our web collection on statistics for biologists contains articles on many of the points above.

Software and code

Policy information about availability of computer code

Data collection

Rheology
DHR3 rheometer (TA Instruments)

NMR
700 MHz Bruker AVANCE NEO
GPC
1260 Infinity (Polymer Standard Service, Mainz)
UV/Vis; Fluorescence
SPARK 20M microplate reader (Tecan Group Ltd).
FCS
LSM 880 (Carl Zeiss)

Nanoindentation
Pavone Nanoindenter DataViewer (Optics11),

ESI
Advion CMS

Microscopy
Keyence BZ-X 800
Leica DM IL LED Fluo microscope

Proteome Profiler
ChemiDoc Imaging System (BioRad)

Data analysis	Rheology<br>TRIOS software 4.0.2.30774<br><br>NMR<br>MestReNova 15.0.1/Bruker Topspin 3.6.1<br><br>GPC<br>WinGPC UniChrom V8.40<br><br>UV/Vis; Fluorescence (Tecan)<br>Sparkcontrol V.2.3<br><br>FCS<br>ConfoCor3 3.0<br><br>Nanoindentation<br>DataViewer (Optics11), V 2.5.7)<br><br>ESI<br>Advion Data Express V. 4.0.13.8<br><br>Microscopy<br>BZ-X800 Viewer V. 1.3.0.1<br><br>Proteome Profiler<br>Image Lab Touch Software V. 3.0.1.14<br><br>Image processing<br>Image J 1.54f

For manuscripts utilizing custom algorithms or software that are central to the research but not yet described in published literature, software must be made available to editors and reviewers. We strongly encourage code deposition in a community repository (e.g. GitHub). See the Nature Portfolio guidelines for submitting code & software for further information.

Data

Policy information about availability of data

All manuscripts must include a data availability statement. This statement should provide the following information, where applicable:

- Accession codes, unique identifiers, or web links for publicly available datasets
- A description of any restrictions on data availability
- For clinical datasets or third party data, please ensure that the statement adheres to our policy

The data generated in this study have been deposited on the open repository Zenodo under accession code DOI: 10.5281/zenodo.22081282

Research involving human participants, their data, or biological material

Policy information about studies with human participants or human data. See also policy information about sex, gender (identity/presentation), and sexual orientation and race, ethnicity and racism.

Reporting on sex and gender	Not applicable
Reporting on race, ethnicity, or other socially relevant groupings	Not applicable
Population characteristics	Not applicable
Recruitment	Not applicable
Ethics oversight	See attached approvals

Note that full information on the approval of the study protocol must also be provided in the manuscript.

Field-specific reporting

Please select the one below that is the best fit for your research. If you are not sure, read the appropriate sections before making your selection.

☒ Life sciences ☐ Behavioural & social sciences ☐ Ecological, evolutionary & environmental sciences

For a reference copy of the document with all sections, see nature.com/documents/nr-reporting-summary-flat.pdf

Life sciences study design

All studies must disclose on these points even when the disclosure is negative.

Sample size	No statistical methods were used to determine sample sizes; samples sizes were determined by allowable error size, accuracy and needed for statistical analysis.
Data exclusions	Exclusions are performed when cells within the well are not confluent enough to begin the experiment.
Replication	All attempts at replication of the data are successful.
Randomization	Experiments were not randomised unless stated otherwise.
Blinding	Blinding is not possible in this study because the macroscopically visible characteristics of the samples mean investigators know the treatment simply by looking at them.

Reporting for specific materials, systems and methods

We require information from authors about some types of materials, experimental systems and methods used in many studies. Here, indicate whether each material, system or method listed is relevant to your study. If you are not sure if a list item applies to your research, read the appropriate section before selecting a response.

Materials & experimental systems

n/a	Involved in the study	
☐	☒	Antibodies
☐	☒	Eukaryotic cell lines
☒	☐	Palaeontology and archaeology
☒	☐	Animals and other organisms
☒	☐	Clinical data
☒	☐	Dual use research of concern
☒	☐	Plants

Methods

n/a	Involved in the study	
☒	☐	ChIP-seq
☒	☐	Flow cytometry
☒	☐	MRI-based neuroimaging

Antibodies

Antibodies used	R&D Systems Proteome Profiler [ARY009]: pre-spotted capture membranes and biotinylated detection antibody cocktail.
Validation	All antibodies in this array are validated for specificity and multiplex compatibility by the manufacturer (R&D Systems)

Eukaryotic cell lines

Policy information about cell lines and Sex and Gender in Research

Cell line source(s)	A549 (DSMZ; ACC 107; 4T1 (ATCC; CRL-2539), Patient-derived glioblastoma cells
Authentication	STR analysis according to the global standard ANSI/ATCC ASN-0002.1-2021 (2021) resulted in an authentic STR profile of the reference STR database.<br>Passages used for experiments are between 3 to 15.<br><br>Patient-derived glioblastoma cells: Brain tumor specimens were collected during surgical removal of tumor tissue from patients diagnosed with a high-grade brain tumor. Only specimens with pathological confirmation of glioblastoma diagnosis were included in the study.
Mycoplasma contamination	A549&4T1: Certified free of Mycoplasma contamination when bought. No further tests for Mycoplasma or 4T1. A549 Mycoplasma test performed after purchase.
Commonly misidentified lines (See ICLAC register)	Cell line used in the study are not within the commonly misidentified lines.

Plants

Seed stocks	Not applicable
Novel plant genotypes	Not applicable
Authentication	Not applicable
