# Supplementary material for: Cell-derived lactate triggers local hydrogelation and phenotype remodelling
Source: Nat Commun. 2026 Sep 28;17:10279. doi: 10.1038/s41467-026-77702-x (PMC13619628; doi:10.1038/s41467-026-77702-x)
Supplement: Supplementary file 3 [file 41467_2026_77702_MOESM3_ESM.pdf]

nature portfolio

Peer Review File

Cell-derived lactate triggers local hydrogelation and phenotype remodelling

Corresponding Author: Professor Tanja Weil

This file contains all reviewer reports in order by version, followed by all author rebuttals in order by version.

Version 0:

Reviewer comments:

Reviewer #1

(Remarks to the Author)
The manuscript “Lactate-instructed Hydrogelation Enables Cell-Driven Matrix and Phenotype Reconfiguration” by M. G. Braun et al. describes an alginate-based system that forms a stiff chemical network upon the release of lactate from tumor cells. This network hinders the migration of the tumor cells out of a tumor spheroid.

Among other techniques, fluorescence correlation spectroscopy (FCS) was used to characterize the local stiffness of the networks, also as a function of the distance from the spheroid. At this, the diffusion time of a fluorescence-labeled protein (bovine serum albumin) through the detection volume of fcs (~ 1 µm3) was measured and set in relation to the one in the neat buffer. A strong gradient was found in a layer extending 1000 µm away from the tumor spheroid surface. The FCS measurements were carried out in a sound way, the protocols are clearly described, and the interpretation is careful and meaningful; hence, the results are significant.

Since I am not an expert in the topic of the study, I refrain from commenting the significance of the approach itself and the results from the other methods used. Just two minor things: (i) The authors should check whether all abbreviations used are spelled out. (ii) In some cases, the number of significant digits given seems too high, e.g. on p. 7: “E = 2,949 ± 1,287 Pa” should read (2.9 +/- 1.3) x 10^3 Pa.

Reviewer #2

(Remarks to the Author)
I co-reviewed this manuscript with one of the reviewers who provided the listed reports. This is part of the Nature Communications initiative to facilitate training in peer review and to provide appropriate recognition for Early Career Researchers who co-review manuscripts.

Reviewer #3

(Remarks to the Author)
This manuscript presents a conceptually innovative study that directly links cellular metabolism to covalent material assembly. The authors exploit lactate secreted by cancer cells to drive an enzymatic oxidation cascade, in which lactate oxidase and horseradish peroxidase convert metabolic flux into hydrogen peroxide–mediated phenol coupling, thereby enabling spatially and temporally resolved hydrogel formation around tumor spheroids. This mechanism closely parallels oxidative crosslinking processes observed during extracellular matrix remodeling and stiffening in cancer, where redox-active enzymes and metabolite gradients shape the local mechanical microenvironment. The points below are intended to refine interpretation and clarity.

1. ^{1}H NMR spectroscopy reveals minor N-acylurea by-products resulting from EDC-mediated activation. Given the use of AlgTA in direct cell culture, it would be important to clarify whether these residual species were assessed for potential cytotoxic effects. Additionally, could the authors comment on whether further purification was explored to reduce these by-products?

2. The SEC/GPC data show a decrease in apparent molar mass following tyramine functionalization. Given that covalent modification at low degrees of substitution would not be expected to reduce molecular weight, how should this observation be interpreted? Please provide the molar mass of each specie and the associated errors of each measurement. How much replicates were ferformed¿

3. Given that resazurin primarily reports cellular redox metabolism and may decrease under adaptive metabolic rewiring without overt loss of viability, an orthogonal cytotoxicity or viability readout would help support the conclusion that the H_2O_2 levels generated are non-cytotoxic.

4. Given that a large fraction of cells within encapsulated spheroids become non-viable, it would be helpful for the authors to clarify the functional objective of the material platform.

5. It is not entirely clear what physical environment constitutes the control condition for the spheroid experiments. Are control spheroids embedded in a non-crosslinked or mechanically inert matrix, or cultured without any surrounding gel?

6. Given that the control condition is based on cell extract, the manuscript would significantly benefit from a proper calibration of lactate concentration and its diffusion/perfusion into the medium. In its current form, the study lacks a calibration curve correlating gel polymerization with defined lactate concentrations. I strongly recommend including experiments in which known concentrations of lactate (instead of cell extract) are applied to the system, followed by quantitative assessment of the degree of gel polymerization. Establishing such a calibration curve would allow the authors to directly correlate the degree of crosslinking with lactate concentration. This would, in turn, enable a more quantitative estimation of lactate production by the cells and provide a more rigorous assessment of their metabolic activity. Without this calibration step, the interpretation of polymerization as a proxy for cellular metabolism remains largely qualitative.

7. How should the reported cell spreading from A549 spheroids be reconciled with the dense, covalently crosslinked nature of the AlgTA matrix, which would typically be expected to limit cell motility? A comparison with a softer or non-crosslinked hydrogel control would help contextualize this behaviour.

8. The proteomic data is only relative e should not be presented as quantitative, details about how data were processed and collected are missing, Some immunofluorescence analysis would illustrate better the matriz remodelling

9. The manuscript would benefit from clearer articulation of the intended applicability of the material system, particularly in light of the pronounced growth arrest and loss of viability observed upon spheroid encapsulation.

10, While the chemistry and conceptual framework are innovative, the intended therapeutic purpose of this development remains insufficiently articulated. The work appears novel; however, a clear and compelling application is not convincingly defined.
The authors are asked to explicitly clarify their primary objective: Is this system envisioned as a therapeutic intervention, a containment strategy, a drug delivery platform, or primarily as a proof-of-concept material responsive to disease-associated metabolites? At present, the translational direction and intended clinical use are ambiguous.
If positioned as a therapeutic alternative for cancer, the manuscript should clearly explain the clinical rationale behind this approach. Given that several well-established and increasingly precise therapeutic strategies already exist (e.g., targeted therapies, immunotherapies, antibody–drug conjugates, locoregional treatments), it is not evident what specific unmet need this material addresses or in what context it would provide a superior or complementary benefit.
Alternatively, if the authors envision a methodological or diagnostic application, this should also be explicitly justified. Notably, robust and widely validated methodologies already exist for monitoring lactate levels, tumour metabolism, viability, hypoxia, and metastatic potential (e.g., metabolic imaging, biosensors, molecular markers). Therefore, the added value of this system over current technologies needs to be clearly defined.
Overall, the manuscript would be significantly strengthened by a more precise statement of intent, a clearly delineated use-case scenario, and a critical discussion positioning this platform within the existing therapeutic and technological landscape.

Reviewer #4

(Remarks to the Author)
The study "Lactate-instructed Hydrogelation Enables Cell-Driven Matrix and Phenotype Reconfiguration" by Braun et al. presents a metabolism-responsive alginate-tyramine (AlgTA) hydrogel system in which enzymatic cascades (LOx/HRP) initiate covalent crosslinking in response to lactate flux. The study aims to link material stiffening to cellular phenotype reconfiguration via a "redox landscape," providing a sophisticated approach to cell-instructed biomaterials.

In the PDF files, I provide details and specific annotations, with side comments, to support the criticism points below.

Major Revisions (as for scientific description and accuracy)

1. Figure 2 Labeling Mismatch
There is a significant discrepancy between the labeling of Figure 2 subplots and the figure caption. Subplots (b), (c), and (d) in the image do not correspond to the descriptions in the text (NMR is b, GPC is c, Schematic is d).

2. Sample volume to Rheological Geometry Discrepancy

It is reported a 35 µL sample volume, for an 8 mm parallel-plate geometry, with a 0.5–0.6 mm gap. Geometrically, this volume exceeds the theoretical capacity of the gap (~25–30 µL). Please clarify the method (e.g., if the samples were trimmed, overfilled meniscus, etc.).

3. LVE Region Definition
The text states the Linear Viscoelastic (LVE) region was "identified at" 1% strain. As the LVE is a range, not a discrete point, the authors should instead define the range (Fig. S7) and state that 1% was selected as a strain amplitude within that range.

4. Kinetics of Gelation (Fig. S10)
Is the data here once-through, or does it stand for n=3? In fact, the authors should recognize that they start already with a gel. Following an internal metastable transformation (induction period) of approximately 5–10 minutes, the material develops a significant secondary structure (increase in the storage modulus). This kinetic profile deserves a brief discussion (and possibly reference to prior work on physical vs. covalent gels) to clarify the observed change in the network's nature.

5. Structural Gap and Ambiguous Notation (SI)
Supplementary Fig. 1 is never cited in the main text and should be linked to the relevant section. Furthermore, the integrals used for DS and G/M calculations (IA, IB, IC) are labeled and depicted inappropriately.

6. Concluding discussion on Redox Landscape (Lines 376-378)
While the conceptual link between "metabolic flux" and "phenotype reconfiguration" is a strong point of the paper, the current narrative relies on shifting between different terms (signaling, landscape, flux) without a concrete bridge. A brief, focused discussion of the relationship between the rate of material gelation and specific biological signaling events should be provided.

Minor Revisions

I. Citation issue
While Ref 13 is mentioned in the discussion, it should be cited specifically in the description of the AlgTA (i.e., the established protocol of Schulz et al.).

II. Data Presentation in SI
The rheological plots in the Supporting Information are currently too small, and the y-axes (almost two orders of magnitude increase in G' axis to accommodate legends).

III. Time evolution of storage modulus (Fig. S9)
The 12-day stability study is presented as a single bar chart. Given the duration of the experiment, a time-course plot would be more appropriate to show the kinetics of the G'/G0' ratio, e.g., modulus vs. 2-day (or 3-day) plot.

IV. Terminology Suggestion
The authors describe the system as "fully synthetic." Given the use of sodium alginate (a natural polysaccharide) and enzymes (HRP/LOx), "bio-hybrid" or "bio-derived" might be more accurate.

Version 1:

Reviewer comments:

Reviewer #1

(Remarks to the Author)
My comments have been taken into account. Hence, from my point of view, the manuscript can be published.

Reviewer #2

(Remarks to the Author)
I co-reviewed this manuscript with one of the reviewers who provided the listed reports. This is part of the Nature Communications initiative to facilitate training in peer review and to provide appropriate recognition for Early Career Researchers who co-review manuscripts.

Reviewer #3

(Remarks to the Author)
The manuscript was profoundly modified and all my concerns and suggestions were observed. Now the manuscript show a correct hypothesis sustained by a bigger data colection. I recomend this paper.

Reviewer #4

(Remarks to the Author)
In their revision, which currently has the working title "Cell-Derived Lactate Triggers Local Hydrogelation and Phenotype

Remodeling," the authors have thoroughly addressed all previous comments.

Their responses to specific points are well-reasoned. I believe that both the manuscript and the Supplementary File have been significantly strengthened by improvements and additional material (e.g. Fig. 5), and that they are now suitable for publication.
I have no further comments or suggestions.

Open Access This Peer Review File is licensed under a Creative Commons Attribution 4.0 International License, which permits use, sharing, adaptation, distribution and reproduction in any medium or format, as long as you give appropriate credit to the original author(s) and the source, provide a link to the Creative Commons license, and indicate if changes were made.
In cases where reviewers are anonymous, credit should be given to 'Anonymous Referee' and the source.
The images or other third party material in this Peer Review File are included in the article's Creative Commons license, unless indicated otherwise in a credit line to the material. If material is not included in the article's Creative Commons license and your intended use is not permitted by statutory regulation or exceeds the permitted use, you will need to obtain permission directly from the copyright holder.
To view a copy of this license, visit https://creativecommons.org/licenses/by/4.0/

MAX PLANCK INSTITUTE
FOR POLYMER RESEARCH

Point-by-Point Response to Reviewers

We thank the reviewers for their constructive evaluation of our manuscript and for their helpful suggestions. In response, we have substantially revised the manuscript and expanded the Supporting Information. The revised version clarifies the functional objective of the material platform, adds additional calibration and control experiments, and refines the interpretation of the biological data. Major changes include a lactate calibration curve for the FCS readout, orthogonal Calcein-AM/propidium iodide viability analysis, stiffness-dependent spheroid controls, expanded SEC/GPC reporting, revised redox-mechanistic interpretation, and additional patient-derived glioblastoma spheroid experiments to support the translational relevance of the containment concept. We also revised the manuscript language throughout to avoid overstatement and to clearly distinguish early cytocompatible gelation from prolonged exposure- and confinement-associated metabolic suppression.

Reviewer #1:

The manuscript "Lactate-instructed Hydrogelation Enables Cell-Driven Matrix and Phenotype Reconfiguration" by M. G. Braun et al. describes an alginate-based system that forms a stiff chemical network upon the release of lactate from tumor cells. This network hinders the migration of the tumor cells out of a tumor spheroid.

Among other techniques, fluorescence correlation spectroscopy (FCS) was used to characterize the local stiffness of the networks, also as a function of the distance from the spheroid. At this, the diffusion time of a fluorescence-labeled protein (bovine serum albumin) through the detection volume of fcs (~ 1 µm3) was measured and set in relation to the one in the neat buffer. A strong gradient was found in a layer extending 1000 µm away from the tumor spheroid surface. The FCS measurements were carried out in a sound way, the protocols are clearly described, and the interpretation is careful and meaningful; hence, the results are significant.

Since I am not an expert in the topic of the study, I refrain from commenting the significance of the approach itself and the results from the other methods used. Just two minor things: (i) The authors should check whether all abbreviations used are spelled out. (ii) In some cases, the number of significant digits given seems too high, e.g. on p. 7: "E = 2,949 ± 1,287 Pa" should read (2.9 +/- 1.3) x 10^3 Pa.

We thank the reviewer for their careful reading and positive assessment of our FCS methodology. Following their suggestions, we have addressed both minor points to improve the clarity and consistency of the manuscript.

(i) We have performed a comprehensive review of the manuscript to ensure that abbreviations are defined at first mention in the main text and figure legends. This includes, among others, extracellular matrix (ECM), lactate oxidase (LOx), horseradish peroxidase (HRP), 1-ethyl-3-(3-dimethylaminopropyl)carbodiimide/N-hydroxysuccinimide (EDC/NHS), size-exclusion chromatography/gel permeation chromatography (SEC/GPC), fluorescence correlation spectroscopy (FCS), interleukin-8 (IL-8), plasminogen activator inhibitor-1 (PAI-1), tissue

inhibitor of metalloproteinases-1 (TIMP-1), and basic fibroblast growth factor (bFGF). These definitions are included in the revised manuscript on pp. 3–5, p. 8 and p. 12.

(ii) We agree with the reviewer's suggestion. We have revised the reporting of numerical values throughout the manuscript to reflect an appropriate number of significant digits relative to the experimental uncertainty. Specifically, the value previously reported as 2,949 ± 1,287 Pa has been rounded to 2.9 ± 1.3 kPa in the revised manuscript on p. 9, in the section "Remote lactate-driven gelation produces mechanically maturing hydrogels." Analogous changes were made throughout the manuscript and figure legends where excessive significant digits were used.

Reviewer #2

I co-reviewed this manuscript with one of the reviewers who provided the listed reports. This is part of the Nature Communications initiative to facilitate training in peer review and to provide appropriate recognition for Early Career Researchers who co-review manuscripts.

We thank the co-reviewer for their contribution to the evaluation of our manuscript.

Reviewer #3

This manuscript presents a conceptually innovative study that directly links cellular metabolism to covalent material assembly. The authors exploit lactate secreted by cancer cells to drive an enzymatic oxidation cascade, in which lactate oxidase and horseradish peroxidase convert metabolic flux into hydrogen peroxide–mediated phenol coupling, thereby enabling spatially and temporally resolved hydrogel formation around tumor spheroids. This mechanism closely parallels oxidative crosslinking processes observed during extracellular matrix remodeling and stiffening in cancer, where redox-active enzymes and metabolite gradients shape the local mechanical microenvironment. The points below are intended to refine interpretation and clarity.

1. ^{1}H NMR spectroscopy reveals minor N-acylurea by-products resulting from EDC-mediated activation. Given the use of AlgTA in direct cell culture, it would be important to clarify whether these residual species were assessed for potential cytotoxic effects. Additionally, could the authors comment on whether further purification was explored to reduce these by-products?

<u>Reply</u>

We appreciate the reviewer's suggestion regarding residual N-acylurea species and further purification. We explored additional purification strategies to reduce these by-products. However, repeated precipitation led to substantial material loss, with approximately 60% yield after three precipitation cycles. Furthermore, additional dialysis with four further water exchanges did not measurably reduce the N-acylurea-associated signals in the ^{1}H NMR spectrum. The minor resonances at 3.40–3.30, 2.10 and 1.27 ppm, which we assign to EDC-derived N-acylurea side-products, therefore appear to be associated with the polymer fraction rather than freely diffusible low-molecular-weight impurities. This additional purification experiment is shown for review purposes in **Fig. R1**.

To address the possible biological relevance of these residual species, we assessed the purified AlgTA material used in the cell experiments, including the residual N-acylurea signals, using two metabolic viability readouts in spheroid culture. Resazurin-based NADH activity and CellTiter-Glo® 3D ATP measurements showed no significant reduction in metabolic activity for cells exposed to AlgTA solutions at 1, 2 or 3 wt% compared with medium controls under the tested material-exposure conditions. These data are now included in the Supporting Information as **Supplementary Figs. 6 and 7** on **p. S5**.

We also revised the main manuscript to state this point more carefully. The revised main text now notes that the purified AlgTA material, including residual N-acylurea-associated signals, retained HRP-mediated crosslinkability and showed no detectable reduction in cell viability under the tested material-exposure conditions. This change is included in the revised manuscript on **p. 5**. Importantly, we no longer describe the complete AlgTA/LOx/HRP system as broadly non-cytotoxic, because prolonged exposure during lactate-driven peroxide generation leads to metabolic suppression and loss of viable cell fraction. This revised interpretation is now included in the Results on **p. 9** and in the Discussion on **p. 18**.

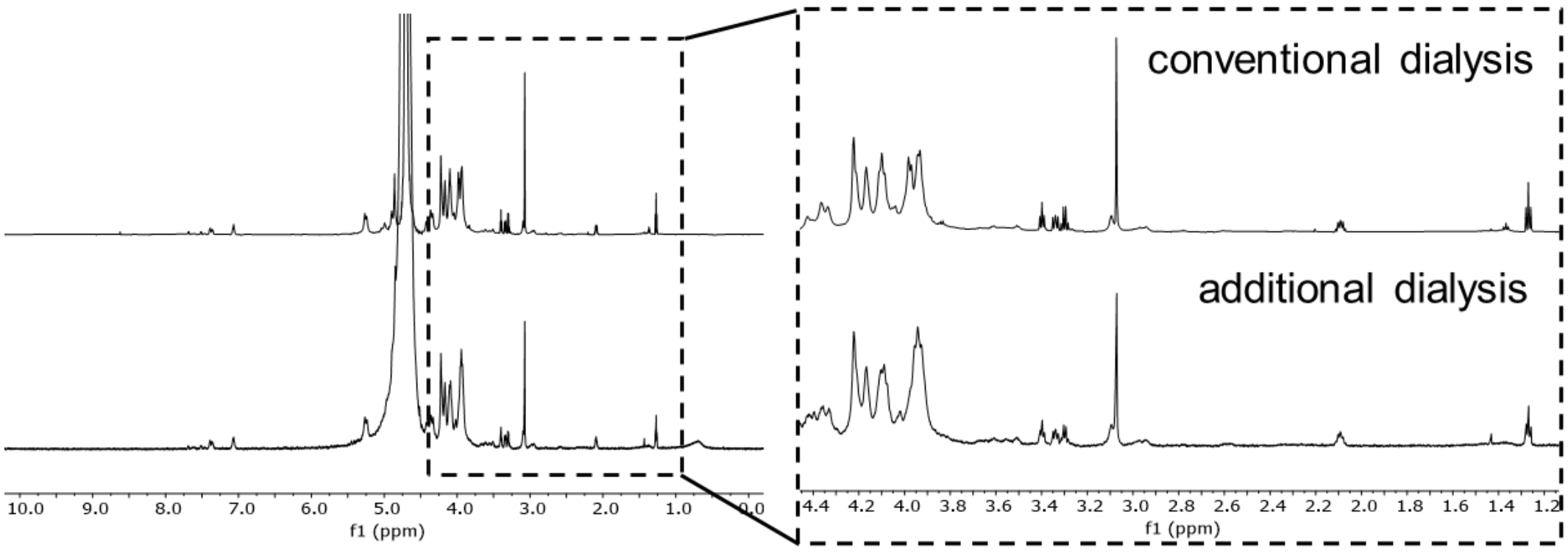

Fig. R1 | Additional purification of AlgTA. ^{1}H NMR spectra (700 MHz, D_2O, 323 K) of AlgTA after standard dialysis and after additional dialysis with four further water exchanges. Full spectra from 0–10 ppm and magnified regions from 1.2–4.4 ppm are shown. Peaks at 3.40–3.30, 2.10 and 1.27 ppm are assigned to EDC-derived N-acylurea side-products. Additional dialysis did not measurably reduce the intensity of these signals.

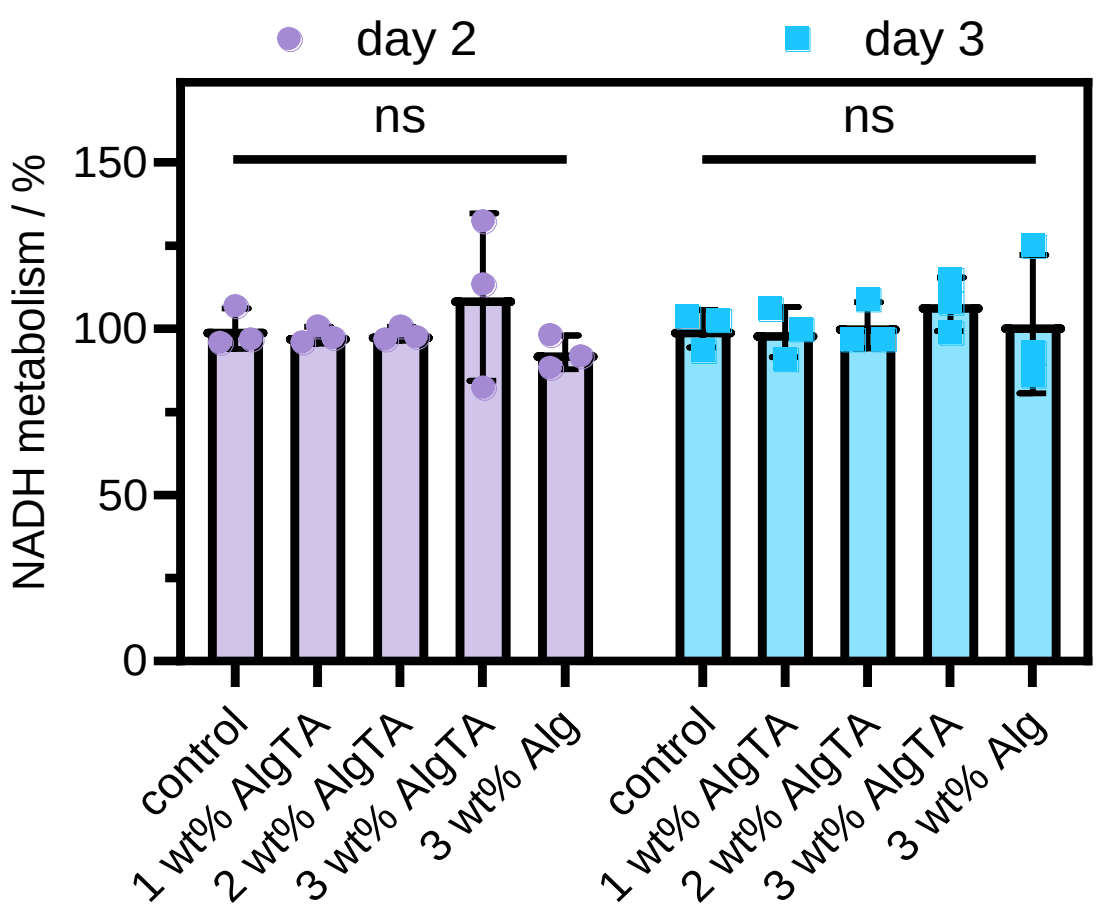

Supplementary Fig. 1 | Metabolic activity of A549 cells in AlgTA solutions. Resazurin-based NADH metabolic activity assay of spheroids cultured in AlgTA solutions (1, 2, and 3 wt%) or dialysed sodium alginate (3 wt%). Metabolic activity was normalised to control spheroids cultured in FBS supplemented medium. Data represent mean ± s.d. from triplicates. No significant reduction in NADH metabolic activity was detected under the tested material-exposure conditions (one-way ANOVA, $P > 0.05$; n = 3 spheroids per condition).

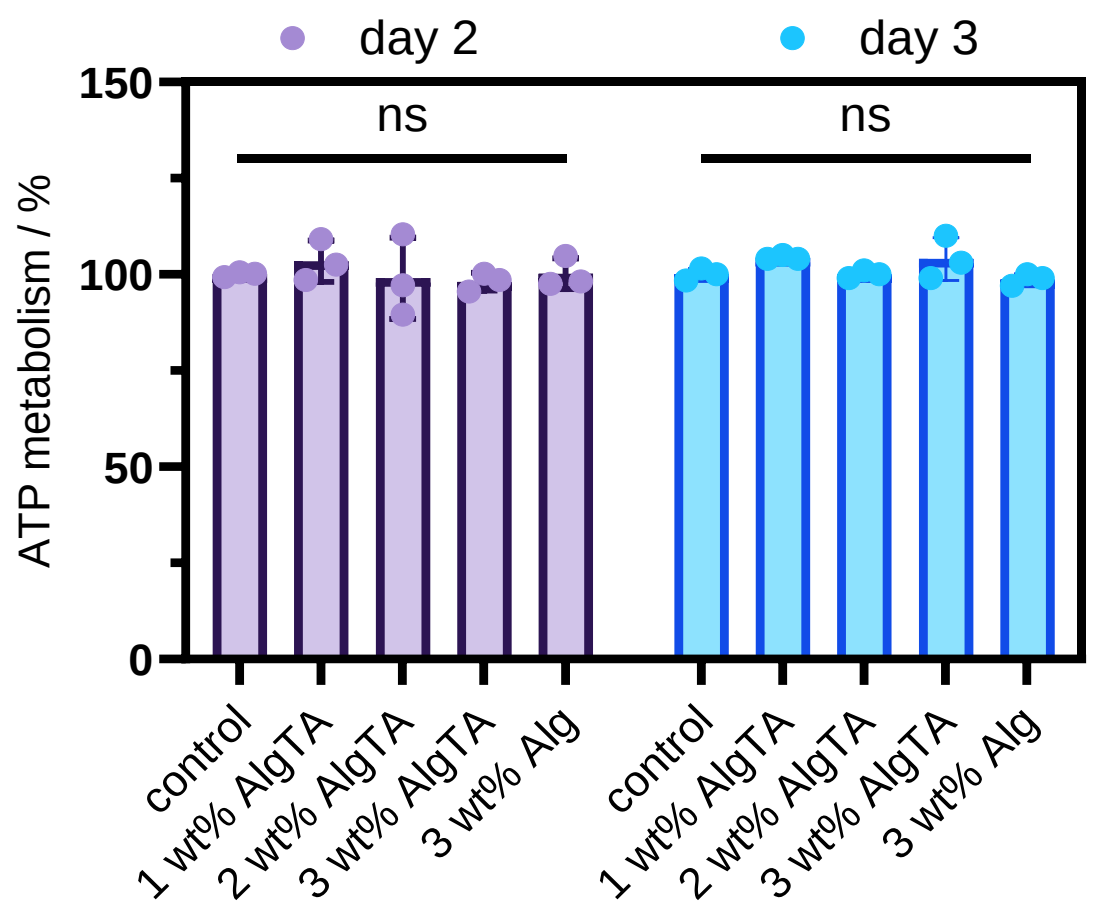

Supplementary Fig. 2 | Metabolic activity of A549 cells in AlgTA solutions. CellTiter Glo-based ATP metabolic activity assay of spheroids cultured in AlgTA solutions (1, 2, and 3 wt%) or dialysed sodium alginate (3 wt%). Metabolic activity was normalized to control spheroids cultured in FBS supplemented medium. Data represent mean ± s.d. from triplicates. No significant reduction in ATP signal was detected under the tested material-exposure conditions (one-way ANOVA, $P > 0.05$; $n = 3$ spheroids per condition).

2. The SEC/GPC data show a decrease in apparent molar mass following tyramine functionalization. Given that covalent modification at low degrees of substitution would not be expected to reduce molecular weight, how should this observation be interpreted? Please provide the molar mass of each specie and the associated errors of each measurement. How much replicates were performed?

<u>Reply</u>

We thank the reviewer for highlighting the apparent molar-mass shift observed by SEC/GPC. We agree that covalent tyramine functionalisation at the low degree of substitution used here is not expected to reduce the actual molecular weight of the polymer backbone. We therefore revised the manuscript to describe the SEC/GPC values explicitly as apparent molar masses obtained by conventional calibration, rather than absolute molar masses. The updated molar-mass data, including peak molar mass (M_p), weight-average molar mass (M_w), number-average molar mass (M_n), dispersity ($Đ$) and associated standard deviations from four measurements, are now provided in **Supplementary Table 1** on **p. S6**. The corresponding SEC/GPC data are discussed in the main manuscript on **p. 5**, and the SEC/GPC traces are shown in **Fig. 2c** on **p. 6**. The M_p shifted slightly from 68.8 ± 2.9 kDa for alginate to 60.0 ± 0.9 kDa for AlgTA, while M_n changed from 43.1 ± 1.1 kDa to 40.6 ± 1.3 kDa. A stronger change was observed for M_w, from 257.7 ± 7.6 kDa to 155.3 ± 2.8 kDa, accompanied by a decrease in dispersity from 5.98 ± 0.16 to 3.83 ± 0.08. We interpret this primarily as a change in apparent hydrodynamic volume and aggregate contribution after purification and functionalisation, rather than as backbone degradation. Conventional aqueous SEC/GPC reports apparent molar mass relative to

calibration standards; therefore, changes in polymer conformation, hydrodynamic volume, aggregation state and interactions with the stationary phase can shift the elution profile without reflecting a true decrease in molecular mass. For this reason, we now refer to these values as apparent molar masses and avoid interpreting SEC/GPC as evidence for absolute molar-mass reduction. The **Fig. 2** caption was also revised to state that the small apparent molar-mass shift is consistent with low substitution and altered hydrodynamic volume after functionalisation.

Supplementary Table 1 | Apparent molar-mass distribution of alginate and AlgTA determined by aqueous SEC/GPC with RI detection (eluent: 0.07 M Na_2HPO_4; pullulan standards; conventional calibration; n = 4).

	Alginate	AlgTA
M_n / kDa	43.1 ± 1.1	40.6 ± 1.3
M_w / kDa	257.7 ± 7.6	155.3 ± 2.8
M_p / kDa	68.8 ± 2.9	60.0 ± 0.9
Đ	5.98 ± 0.16	3.83 ± 0.08

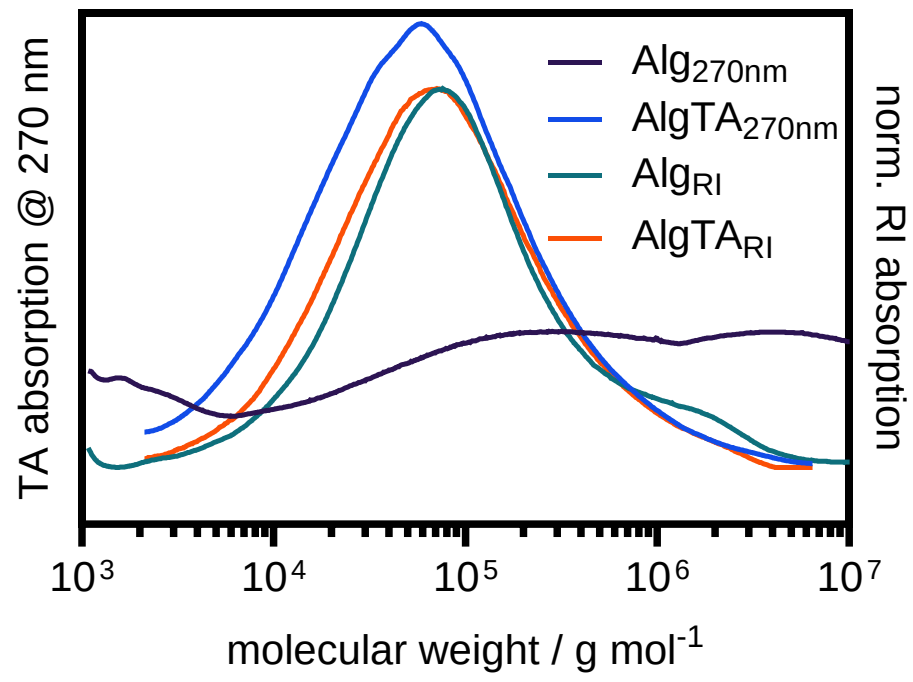

Fig. 2 | Synthesis and peroxide-triggered gelation of phenol-functionalised alginate (AlgTA).
(c) Aqueous SEC/GPC traces (RI and UV 270 nm) for alginate and AlgTA (eluent: 0.07 M Na_2HPO_4; pullulan standards; conventional calibration). Co-elution with the UV signal supports polymer-bound tyramine; the small apparent molar-mass shift is consistent with low substitution and altered hydrodynamic volume after functionalisation.

The molar mass of aqueous SEC/GPC traces (RI) for alginate (Alg) and AlgTA (**Fig. R2**; (eluent: 0.07 M Na_2HPO_4; pullulan standards; conventional calibration) in quadruplicates as well as for dextran (Dex) and DexTA (**Fig. R3**, **Table R1**, eluent: 0.1 M $NaNO_3$; dextran standards; conventional calibration) in duplicates are given below.

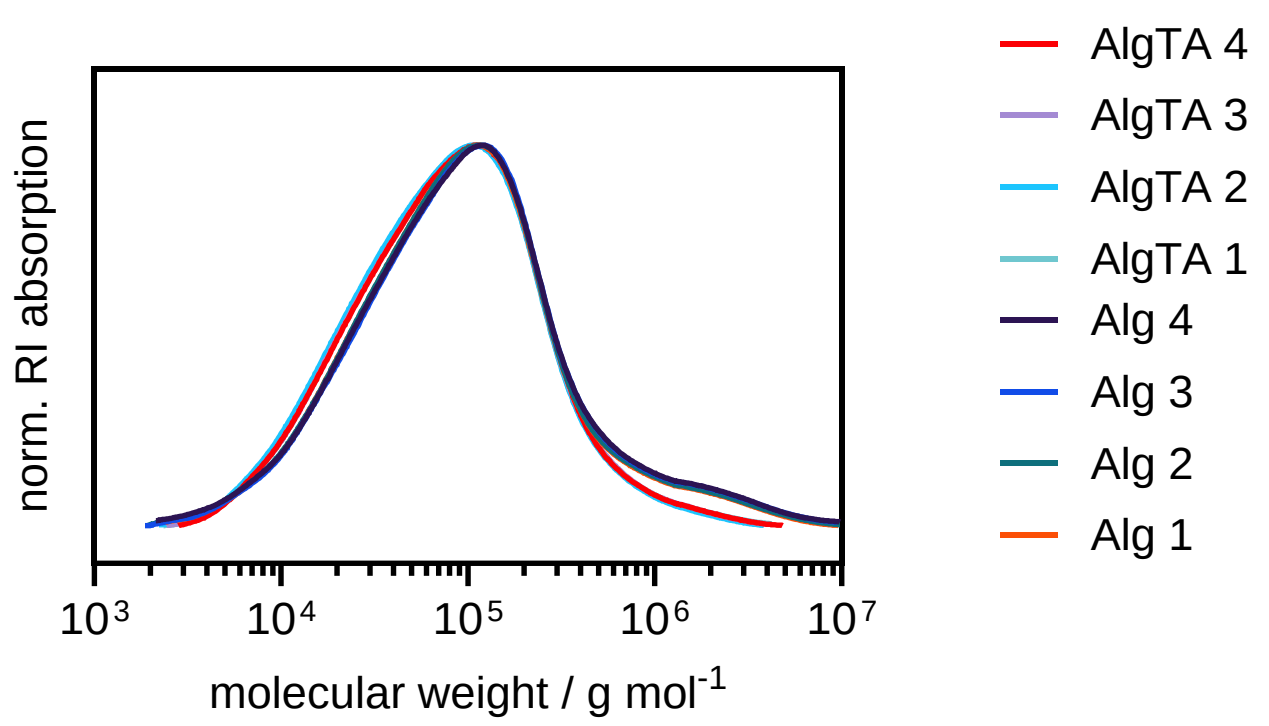

Fig. R2 Aqueous SEC/GPC traces (RI) for alginate (Alg) and AlgTA (eluent: 0.07 M Na_2HPO_4; pullulan standards; conventional calibration), n=4.

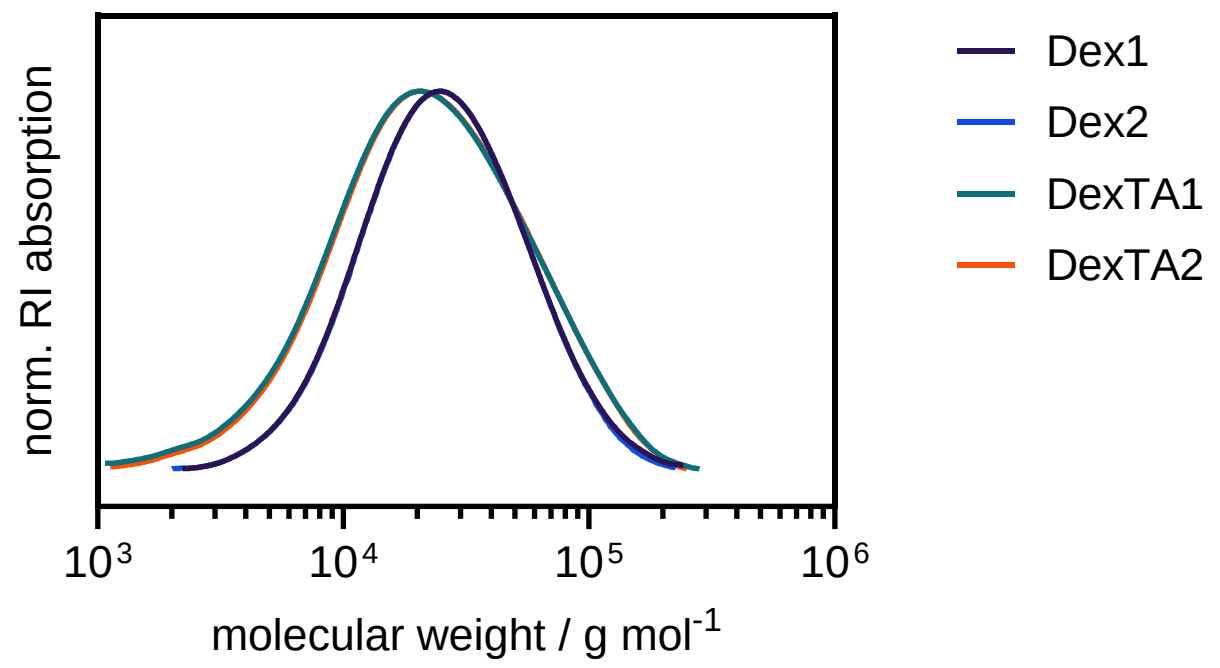

Fig. R3 | Aqueous SEC/GPC traces (RI) for dextran (Dex) and DexTA (eluent: 0.1 M $NaNO_3$; dextran standards; conventional calibration). n=2.

Table R1 | Molecular weight distribution of dextran before and after tyramine functionalization. Values represent the mean ± standard deviation of two replicates (n=2). The apparent molecular weight decreses after functionalization of the polymer (DexTA) due to the altered intramolecular forces.

	Dex	DexTA
M_n / kDa	19.0 ± 0.1	14.4 ± 0.3
M_w / kDa	33.8 ± 0.5	32.7 ± 0.2
M_p / kDa	22.8 ± 0.1	18.9 ± 0.2
Đ	1.78 ± 0.02	2.27 ± 0.03

3. Given that resazurin primarily reports cellular redox metabolism and may decrease under adaptive metabolic rewiring without overt loss of viability, an orthogonal cytotoxicity or viability readout would help support the conclusion that the H_2O_2 levels generated are non-cytotoxic.

Reply

We agree that an orthogonal method is required to distinguish altered redox metabolism from loss of viable cell fraction. Accordingly, we performed Calcein-AM/propidium iodide staining as a metabolism-independent viability readout and compared it with the resazurin assay. These data are now shown in **Supplementary Fig. 18** on **p. S10** and are discussed together with the resazurin data shown in **Fig. 3c** on **pp. 10–11** of the revised manuscript.
To capture the cumulative effect of the system on the full cell population, the medium was not exchanged during the experiment. Both readouts showed consistent time-dependent trends. On day 1, viable cell fraction remained high by Calcein/PI staining and resazurin activity was only moderately reduced. By day 2, both assays indicated reduced cellular activity/viable fraction, and by day 3, both assays showed pronounced loss of viability or metabolic activity. Specifically, Calcein/PI-positive cells changed from 100.3 ± 0.2% on day 1 to 91.5 ± 6.6% on day 2 and 4.5 ± 2.3% on day 3, while resazurin activity changed from 90.0 ± 13.6% on day 1 to 55.5 ± 19.9% on day 2 and 15.1 ± 4.8% on day 3.
We therefore revised the manuscript to avoid the broad conclusion that the H_2O_2-generating system is non-cytotoxic. Instead, the revised text now states that early gelation is compatible with cells, whereas prolonged exposure under closed-culture conditions leads to progressive metabolic suppression and loss of viable cell fraction. This revised interpretation is included in the Results on **p. 9**, in the figure legend of **Fig. 3** on **pp. 10–11**, and in the Discussion on **p. 18**.

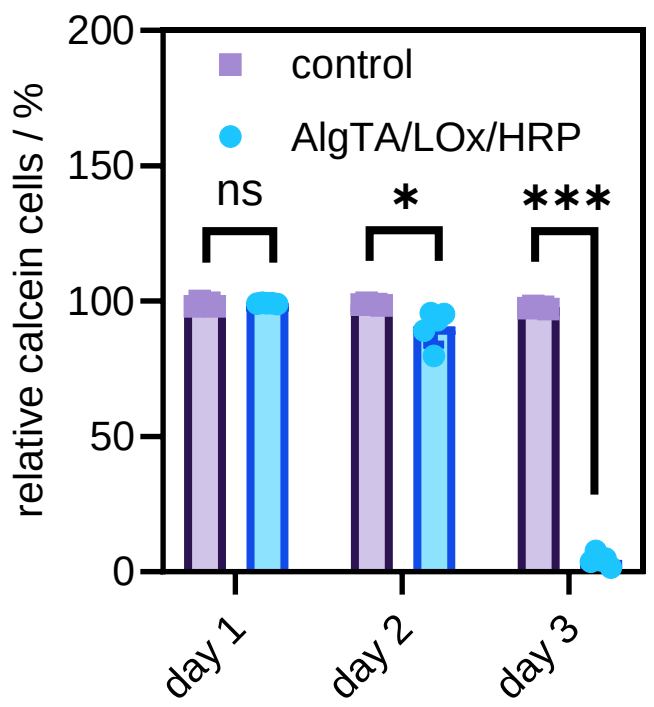

Supplementary Fig. 3 | Relative fraction of calcein positive A549 cells during transwell lactate-gated gelation. Data were quantified from five images per condition and day, with >1500 cells analysed per condition and day. Shown *P* values: day 1, *P* = 0.433; day 2, *P* = 0.021; day 3, *P* < 0.001. Data are mean ± s.d.; Statistics: unpaired two-tailed Welch's t-test; *P* < 0.05 (*), *P* < 0.01 (**), *P* < 0.001 (***), *P* > 0.05 (ns, not significant).

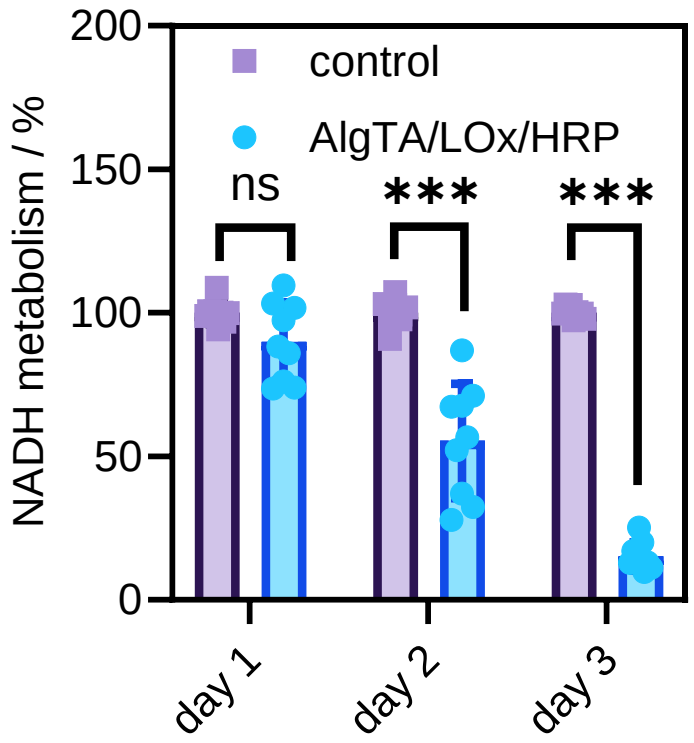

Fig. 3c | Endogenous lactate drives peroxide-mediated crosslinking and *in situ* gelation.
Unless stated otherwise, data are mean ± s.d. Statistics: unpaired two-tailed Welch's t-test; $P < 0.05$ (*), $P < 0.01$ (**), $P < 0.001$ (***), $P > 0.05$ (ns, not significant).
(c) Resazurin-based metabolic activity (n = 9 wells) from 3 independent experiments. Shown P values: day 1, P = 0.054, day 2, P < 0.001; day 3, P < 0.001.

4. Given that a large fraction of cells within encapsulated spheroids become non-viable, it would be helpful for the authors to clarify the functional objective of the material platform.

Reply

We thank the reviewer for raising this important point. The revised manuscript now clarifies that the system is not intended as a conventional tissue-engineering scaffold that supports long-term cell expansion. Instead, the functional objective is to create a metabolism-gated, bio-synthetic containment material in which highly glycolytic cell populations locally trigger covalent matrix formation and mechanical confinement.

In this context, growth arrest and reduced viability in dense spheroids are not interpreted as scaffold failure, but as part of the intended confinement response. The material translates local lactate flux into matrix formation, transport restriction and mechanical containment, thereby limiting spheroid expansion and invasion in vitro. This objective is now stated explicitly in the revised Results and Discussion. Spheroid expansion and metabolic activity are discussed in the section **"Pericellular gelation suppresses spheroid expansion and metabolic activity"** on **p. 14**, with the corresponding data shown in **Fig. 4g,h** on **pp. 13–14**. The broader containment objective is further developed in the integration paragraph on **p. 17** and in the Discussion on **pp. 18–19**.

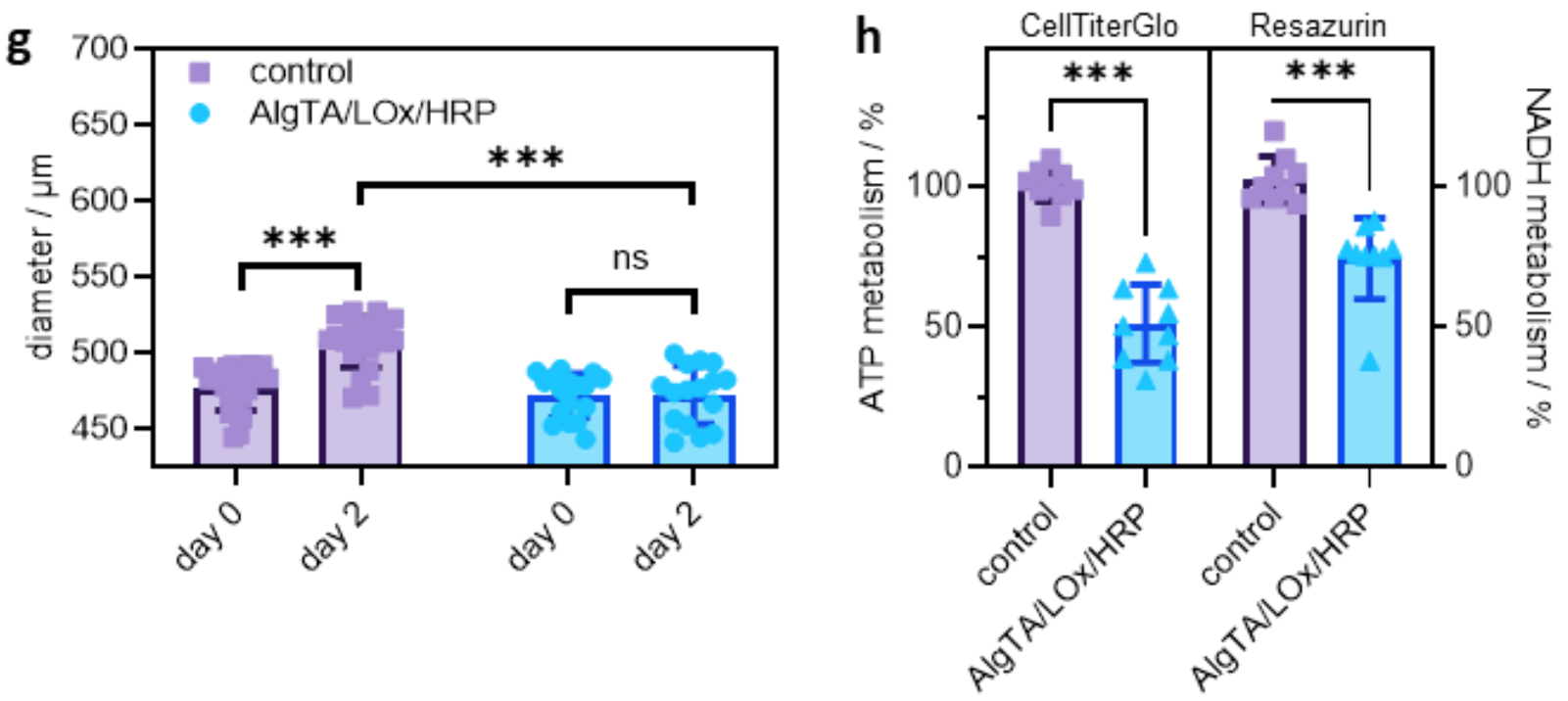

Fig. 4 | Lactate-gated AlgTA encapsulation of A549 spheroids redirects cell state.
Unless stated otherwise, data are mean ± s.d. (day 2). Statistics: unpaired two-tailed Welch's t-test; $P < 0.05$ (*), $P < 0.01$ (**), $P < 0.001$ (***), $P > 0.05$ (ns, not significant). Formulation (where applicable): 3 wt% AlgTA, 10 U mL^{-1} HRP, 1 U mL^{-1} LOx; control: DMEM supplemented with FBS.
(g) Spheroid diameter with and without AlgTA/LOx/HRP after 2 days; ≥ 16 spheroids per condition. Shown *P* values: AlgTA/LOx/HRP day 0 vs day 2, $P = 0.546$; control day 0 vs day 2, $P < 0.001$; AlgTA/LOx/HRP day 2 vs control day 0, $P < 0.001$.
(h) Metabolism under encapsulation: CellTiter-Glo® 3D (ATP, n = 9, $P < 0.001$, 3 independent experiments) and resazurin (NADH, n = 9, $P < 0.001$, 3 independent experiments) assays.

5. It is not entirely clear what physical environment constitutes the control condition for the spheroid experiments. Are control spheroids embedded in a non-crosslinked or mechanically inert matrix, or cultured without any surrounding gel?

__**Reply**__
We thank the reviewer for pointing out this ambiguity. To clarify the physical environment of the control condition, A549 and 4T1 control spheroids were cultured in standard growth medium without AlgTA, LOx, HRP or any surrounding gel/polymer matrix. This control therefore represents a mechanically unconstrained condition and allows comparison with the lactate-gated hydrogelation condition. We now state this explicitly in the Methods on **p. 26** and in the relevant figure legends.
__The revised Methods state:__
"Control spheroids were cultured in Dulbecco's Modified Eagle's Medium (DMEM, high glucose) supplemented with 10% dialysed FBS and 1% penicillin/streptomycin, without AlgTA, LOx, HRP or surrounding gel."
The figure legends were also harmonised. **Fig. 4** now states the A549 spheroid control condition on **p. 13**, and **Fig. 5** now states that controls were cultured in medium without AlgTA/LOx/HRP on **p. 17**. For the patient-derived glioblastoma invasion assay, the revised Methods specify that control wells received cDMEM only; this is included on **p. 30**. We also acknowledge in the revised interpretation that this control is not a matrix-matched inert

control. To address matrix stiffness more directly, we added additional spheroid experiments in softer AlgTA gels of 1 and 2 wt%, which show that protrusions occur only in the stiffer 3 wt% matrix. These data are now included as **Supplementary Fig. 23** on **p. S11** and **Supplementary Fig. 24** on **p. S12**, and are discussed in the main manuscript on **p. 12**.

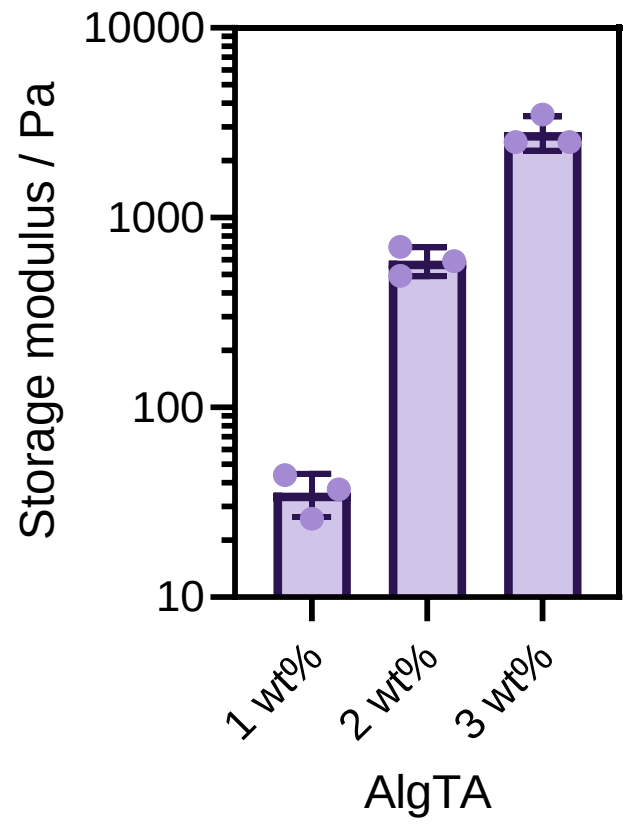

Supplementary Fig. 4 | Storage moduli of AlgTA hydrogels prepared with varied polymer concentrations. Storage modulus G′ of AlgTA hydrogels prepared with 1, 2 or 3 wt% AlgTA in culture medium. Data are mean ± s.d. (n = 3 independent gel formulations).

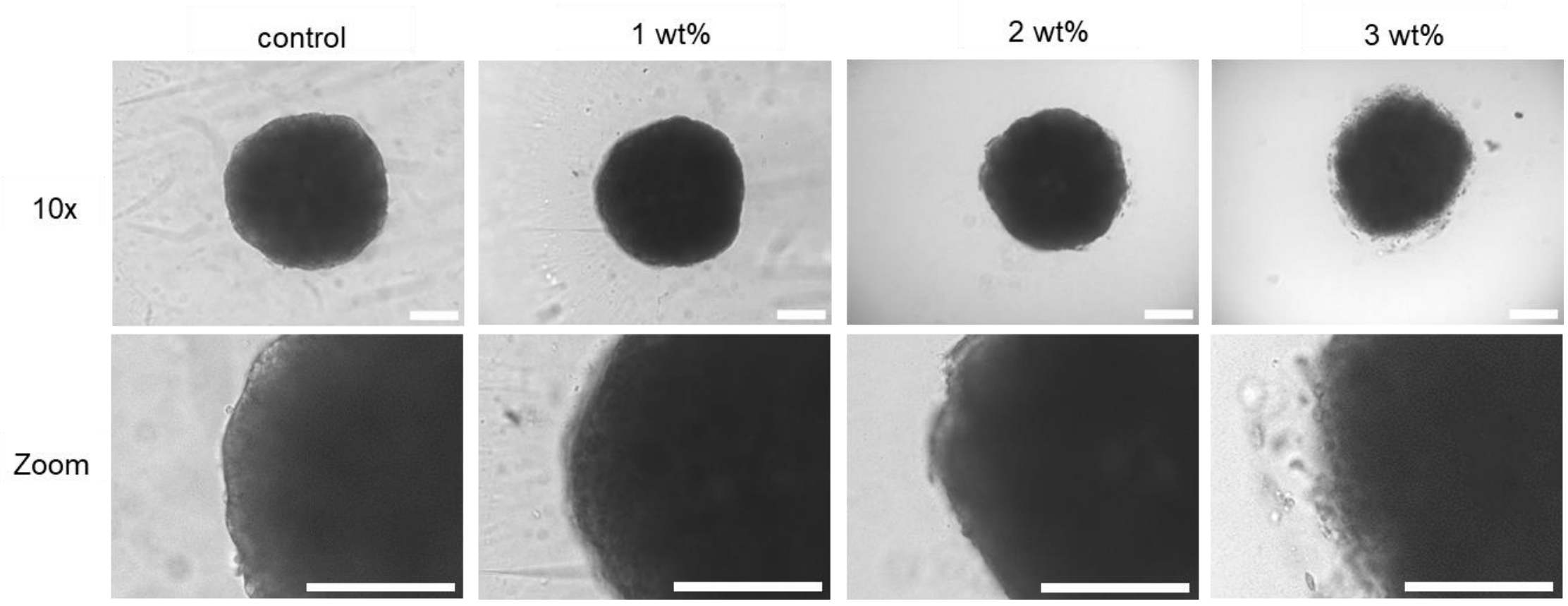

Supplementary Fig. 5 | Stiffness-dependent cell spreading of A549 spheroids. Spheroid morphology as a function of polymer concentration and matrix stiffness (1 wt%, 35 ± 9 Pa; 2 wt%, 600 ± 100 Pa; 3 wt%, 2.8 ± 0.6 kPa, Supplementary Fig. 23). Representative images of spheroids after 2 days show prominent cell protrusions in the stiffest 3 wt% matrix, whereas protrusions are absent in hydrogels with concentrations of 2 wt% or lower. Scale bar, 200 µm.

6. Given that the control condition is based on cell extract, the manuscript would significantly benefit from a proper calibration of lactate concentration and its diffusion/perfusion into the medium. In its current form, the study lacks a calibration curve correlating gel polymerization with defined lactate concentrations. I strongly recommend including experiments in which known concentrations of lactate (instead of cell extract) are applied to

the system, followed by quantitative assessment of the degree of gel polymerization. Establishing such a calibration curve would allow the authors to directly correlate the degree of crosslinking with lactate concentration. This would, in turn, enable a more quantitative estimation of lactate production by the cells and provide a more rigorous assessment of their metabolic activity. Without this calibration step, the interpretation of polymerization as a proxy for cellular metabolism remains largely qualitative.

Reply

We entirely agree that a calibration between defined lactate concentration and gelation is essential for a more quantitative interpretation of cell-driven material formation. To address this, we performed a calibration study using defined concentrations of exogenous sodium lactate under matched AlgTA/LOx/HRP conditions. We measured the resulting FCS diffusion-time ratio, τ_{gel}/τ_{buffer}, and established a calibration curve between known lactate concentration and local transport restriction. This calibration is now shown in **Fig. 4c** on **p. 13**, and the spatial spheroid-derived gelation map is shown in **Fig. 4d** on **pp. 13–14**.

Because the FCS readout reports the final network state after enzymatic lactate oxidation and phenol coupling, we now describe the calibrated quantity as an apparent lactate-equivalent concentration, $c_{lac,eq}$, rather than as a direct measurement of absolute lactate production or a time-integrated lactate concentration. This terminology avoids overinterpretation while still providing a calibrated link between lactate availability and local network formation.

Using this calibration, we estimated radial $c_{lac,eq}$ values in spheroid-derived gels. Near the spheroid surface, within 100–400 µm, $\tau_{gel}/\tau_{buffer} \approx 2.2 \pm 0.1$ corresponded to $c_{lac,eq} \geq 5$ mM. At 500 µm, τ_{gel}/τ_{buffer} decreased to 1.8 ± 0.1, corresponding to $c_{lac,eq} = 3.8 \pm 0.3$ mM. At 1000 µm, τ_{gel}/τ_{buffer} reached approximately 1.00 ± 0.01, corresponding to $c_{lac,eq} \leq 0.5$ mM and marking the outer boundary of detectable gelation.

The revised manuscript now uses the subheading **“FCS resolves radial network-density and lactate-equivalent gradients”** and reports $c_{lac,eq}$ throughout this section and in the **Fig. 4** legend. These changes are included in the revised manuscript on **pp. 11–14**. The corresponding calibration protocol is included in the Methods on **p. 23**.

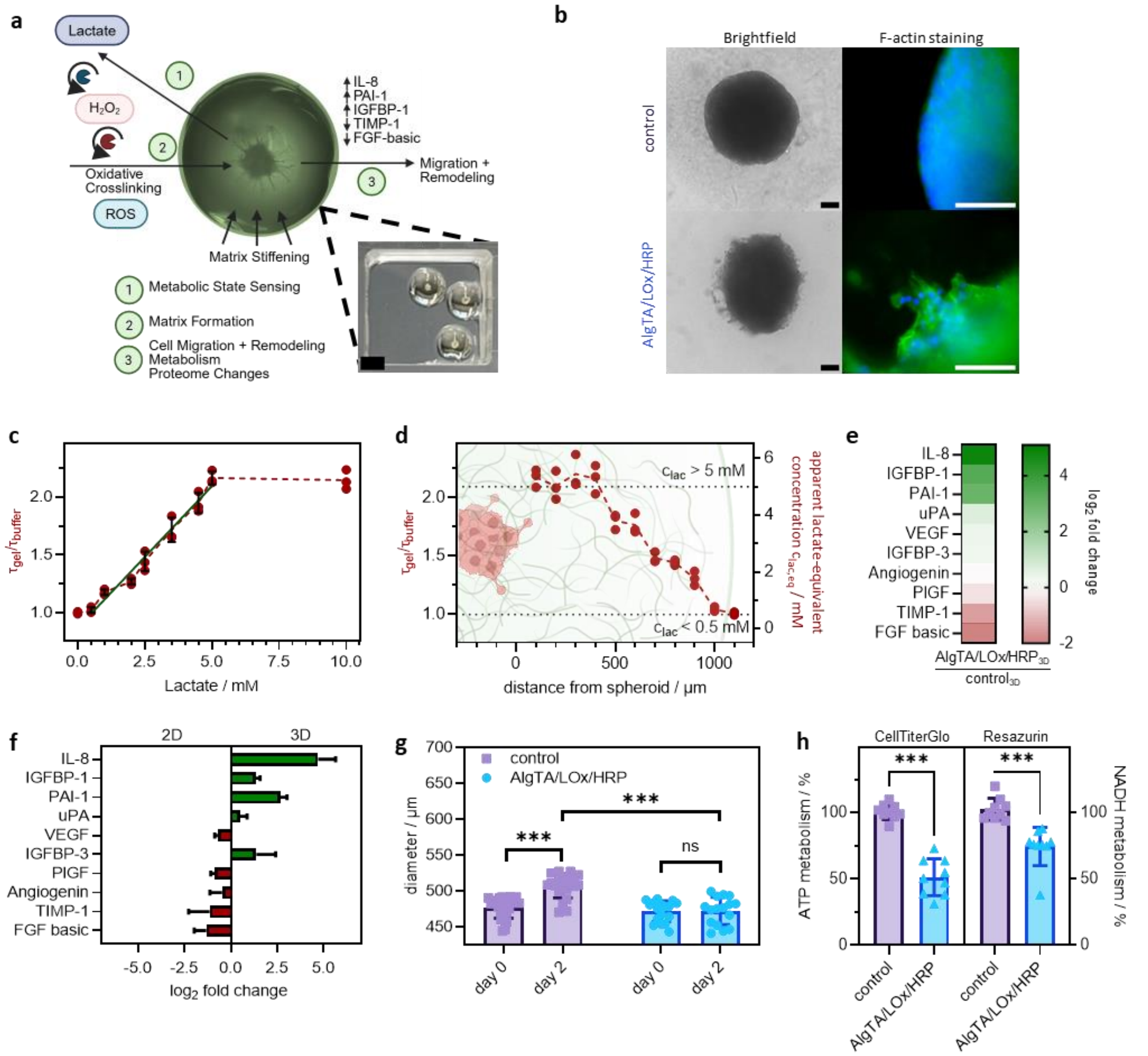

Fig. 4 | Lactate-gated AlgTA encapsulation of A549 spheroids redirects cell state.
Unless stated otherwise, data are mean ± s.d. (day 2). Statistics: unpaired two-tailed Welch's t-test; $P < 0.05$ (*), $P < 0.01$ (**), $P < 0.001$ (***), $P > 0.05$ (ns, not significant). Formulation (where applicable): 3 wt% AlgTA, 10 U mL^{-1} HRP, 1 U mL^{-1} LOx; control: DMEM supplemented with FBS.

(a) Communication cascade: spheroid-secreted lactate is converted by LOx/HRP to H_2O_2, driving phenol coupling and in situ AlgTA gelation around the spheroid (schematic) and photograph of encapsulated spheroids after 2 days. Scale bar, 2 mm.

(b) Fluorescence micrographs of A549 spheroids: F-actin (phalloidin, green) and nuclei (NucBlue, blue). Top: control (regular medium). Bottom: AlgTA/LOx/HRP, showing actin-rich protrusions into the gel. Scale bars, 100 µm.

(c) Calibration of hydrogel crosslinking with defined lactate concentrations. Correlation between the diffusion time ratio of Alexa488-BSA (τ_{gel}/τ_{buffer}) and known concentrations of lactate (0-10 mM sodium lactate). The calibration curve (0.5 – 5 mM lactate) follows the linear equation y = 0.245x + 0.869 with a correlation coefficient $R^2 = 0.9625$; n = 3 positions per gel.

(d) Spatial transport mapping by FCS: diffusion-time ratio (τ_{gel}/τ_{buffer}) and apparent lactate-equivalent concentration ($c_{lac,eq}$) *versus* distance from the spheroid surface, revealing a gradient in local crosslinking (Methods). Individual values were taken at three parallel offset distances and are shown with a line representing the mean.
(e) Proteomics (3D, day 2): relative abundance of selected tumour-associated proteins in AlgTA/LOx/HRP versus control (3 biological replicates, 12 spheroids per biological replicate, 2 on-membrane replicates). Shades of red indicate decreased, and shades of green indicate increased relative protein abundance.
(f) Proteomic profiling: comparison of selected protein responses in 2D transwell cultures and 3D spheroid encapsulation under AlgTA/LOx/HRP conditions. Filled bars indicate the condition showing the stronger relative response.
(g) Spheroid diameter with and without AlgTA/LOx/HRP after 2 days; ≥ 16 spheroids per condition. Shown P values: AlgTA/LOx/HRP day 0 vs day 2, $P = 0.546$; control day 0 vs day 2, $P < 0.001$; AlgTA/LOx/HRP day 2 vs control day 0, $P < 0.001$.
(h) Metabolism under encapsulation: CellTiter-Glo® 3D (ATP, n = 9, $P < 0.001$, 3 independent experiments) and resazurin (NADH, n = 9, $P < 0.001$, 3 independent experiments) assays.

7. How should the reported cell spreading from A549 spheroids be reconciled with the dense, covalently crosslinked nature of the AlgTA matrix, which would typically be expected to limit cell motility? A comparison with a softer or non-crosslinked hydrogel control would help contextualize this behaviour.

Reply

We thank the reviewer for this important question regarding the relationship between matrix stiffness and cell motility. To address this point, we compared A549 spheroid morphology in AlgTA hydrogels prepared with different polymer concentrations: 1, 2 and 3 wt%. These formulations produced increasing stiffnesses of 35 ± 9 Pa, 600 ± 100 Pa and 2.8 ± 0.6 kPa, respectively. The corresponding storage moduli are now shown in **Supplementary Fig. 23** on **p. S11**, and the stiffness-dependent spheroid morphology is shown in **Supplementary Fig. 24** on **p. S12**. We observed that actin-rich protrusions were prominent only in the stiffer 3 wt% AlgTA matrix. In contrast, the softer 1 and 2 wt% gels did not support comparable protrusive morphology, despite offering lower bulk mechanical resistance. These findings suggest that the protrusions are not simply a consequence of reduced physical resistance, but depend on a minimum matrix stiffness that enables traction generation and force-dependent morphogenesis. At the same time, encapsulated spheroids maintained a stable diameter, whereas control spheroids in regular medium expanded over the same period. Thus, the matrix can restrict bulk spheroid expansion while still permitting localised, stiffness-dependent protrusive activity at the spheroid–matrix interface.
We have revised the main manuscript to discuss this point in the section **"Local gelation redirects proteomic and morphological responses"** on **p. 12**. The revised text states:

"Notably, the protrusions were absent in softer gels (1 or 2 wt% AlgTA; 35 ± 9 or 600 ± 100 Pa), suggesting that a threshold of mechanical stiffness is required to trigger these morphogenetic changes."

8. The proteomic data is only relative e should not be presented as quantitative, details about how data were processed and collected are missing, Some immunofluorescence analysis would illustrate better the matriz remodelling

Reply

We thank the reviewer for this important clarification. The protein analysis was performed using the Proteome Profiler Human Angiogenesis Array Kit, which is an antibody-based chemiluminescence array. We agree that this method provides relative, semi-quantitative protein-abundance information rather than absolute quantitative proteomics. We have revised the manuscript, figure legends and Methods accordingly and now consistently refer to these data as "relative proteome profiling" or "relative protein abundance."

To improve transparency, we expanded the Methods section to describe sample preparation, replicate structure, array processing and quantification. The revised Methods now state that array data were interpreted as relative, semi-quantitative protein-abundance changes; that 2D cultures were analysed using two biological replicates with two on-membrane technical replicates; that 3D spheroids were analysed using three biological replicates, each consisting of 12 pooled spheroids, with two on-membrane technical replicates; and that the AlgTA/LOx/Cat control experiment was analysed using two biological replicates with two on-membrane technical replicates. The revised Methods also state that chemiluminescent signals were quantified by background-subtracted mean pixel density of duplicate spots and processed identically across membranes. These details are included in the Methods on **pp. 28–29**.

We also moderated the biological interpretation of the proteome-array data. Rather than presenting the array results as definitive proof of pathway activation, we interpret them together with orthogonal readouts, including phalloidin-based morphology, spheroid-size analysis, ATP/resazurin measurements and live/dead staining. The revised proteome data are shown in **Fig. 3d** on **pp. 10–11**, **Fig. 4e,f** on **pp. 13–14**, and **Supplementary Figs. 19, 22 and 26** on **pp. S10–S13**.

Regarding the reviewer's suggestion for immunofluorescence analysis, the revised manuscript includes phalloidin staining to visualise actin-rich protrusions and matrix-associated morphogenesis. These data are shown in **Fig. 4b** (see above) on **p. 13** and in **Supplementary Fig. 24** (see above) on **p. S12**. We did not add marker-specific immunofluorescence validation for individual proteome-array hits; instead, we now explicitly treat the proteome array as relative, semi-quantitative profiling and interpret it together with the orthogonal morphological and viability data.

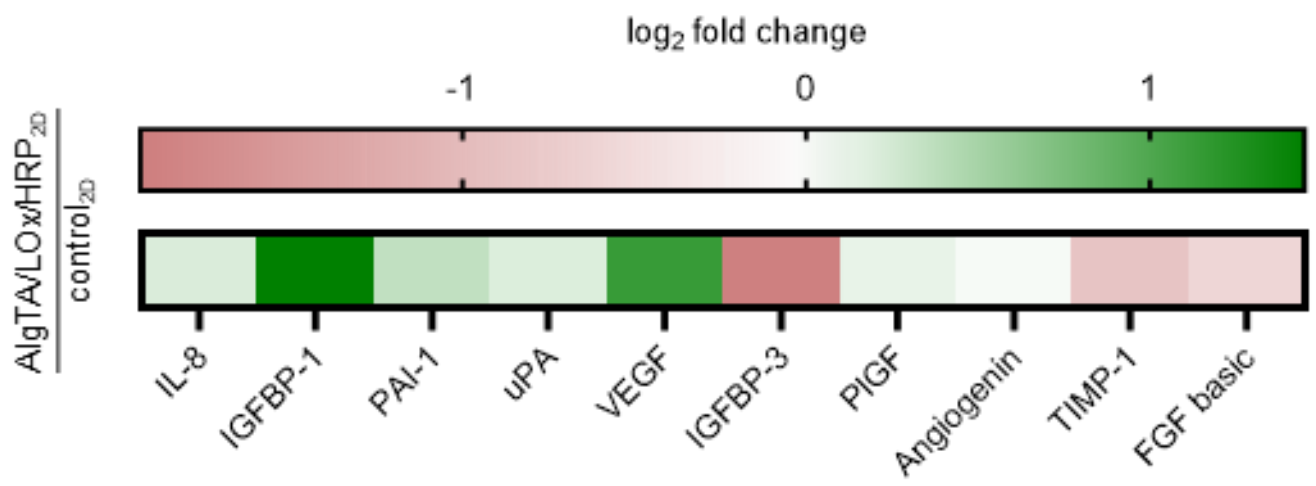

Fig. 3 | Endogenous lactate drives peroxide-mediated crosslinking and *in situ* gelation.
Unless stated otherwise, data are mean ± s.d. Statistics: unpaired two-tailed Welch's t-test; $P < 0.05$ (*), $P < 0.01$ (**), $P < 0.001$ (***), $P > 0.05$ (ns, not significant).
(d) Relative proteome profiling of selected tumour-associated proteins in AlgTA/LOx/HRP-treated cultures at day 2 versus matched controls (2 biological replicates, 2 on-membrane replicates). Shades of red indicate decreased, and shades of green indicate increased relative protein abundance.

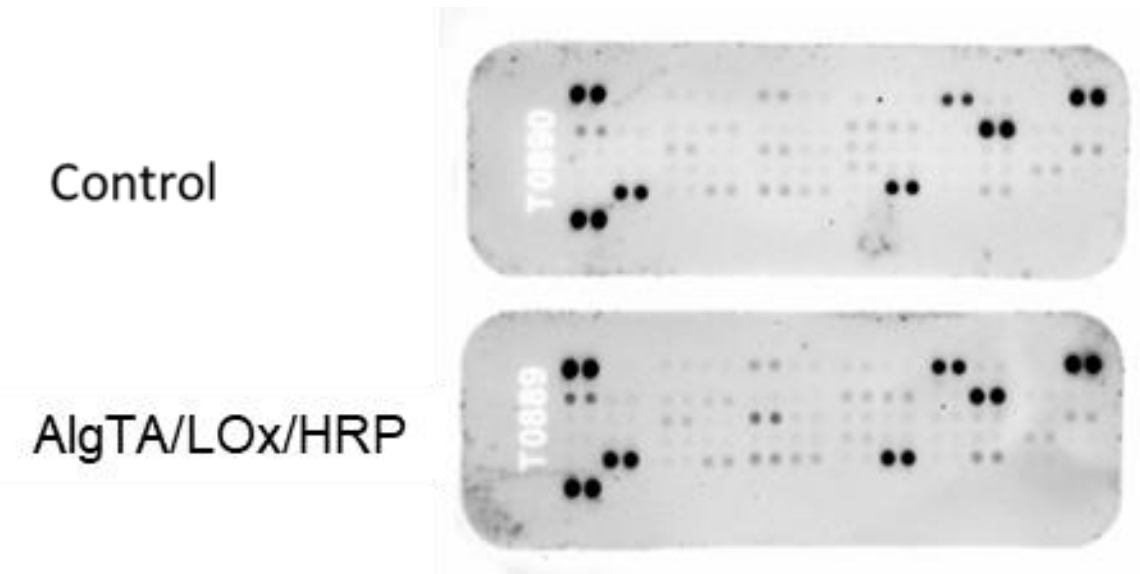

Supplementary Fig. 6 | Proteome Profiler membrane of 2D A549 cell experiments after 2 days of incubation.

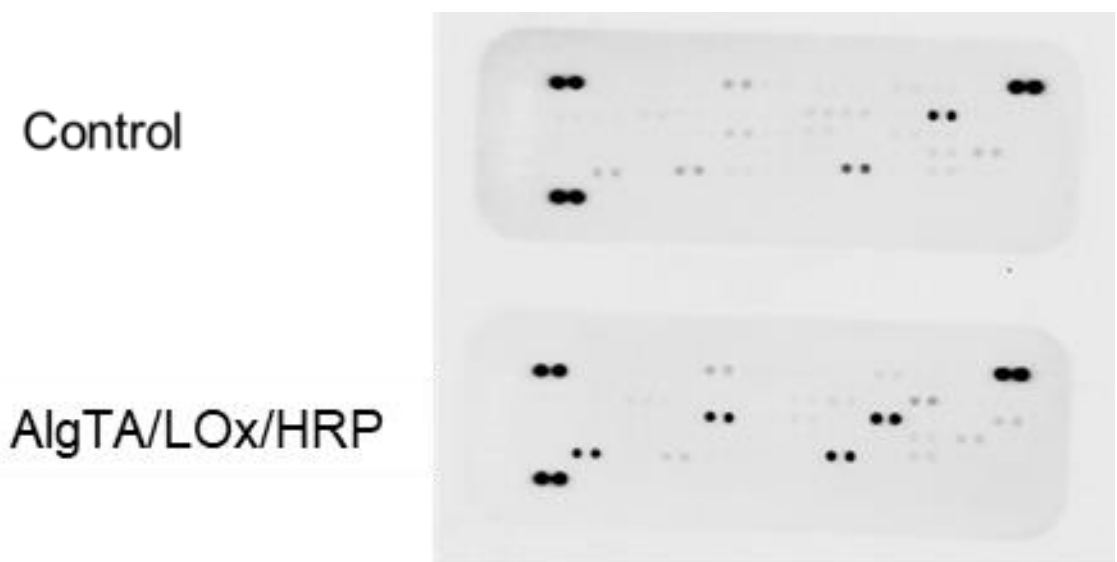

Supplementary Fig. 7 | Proteome Profiler membrane of 3D A549 spheroid experiments after 2 days of incubation.

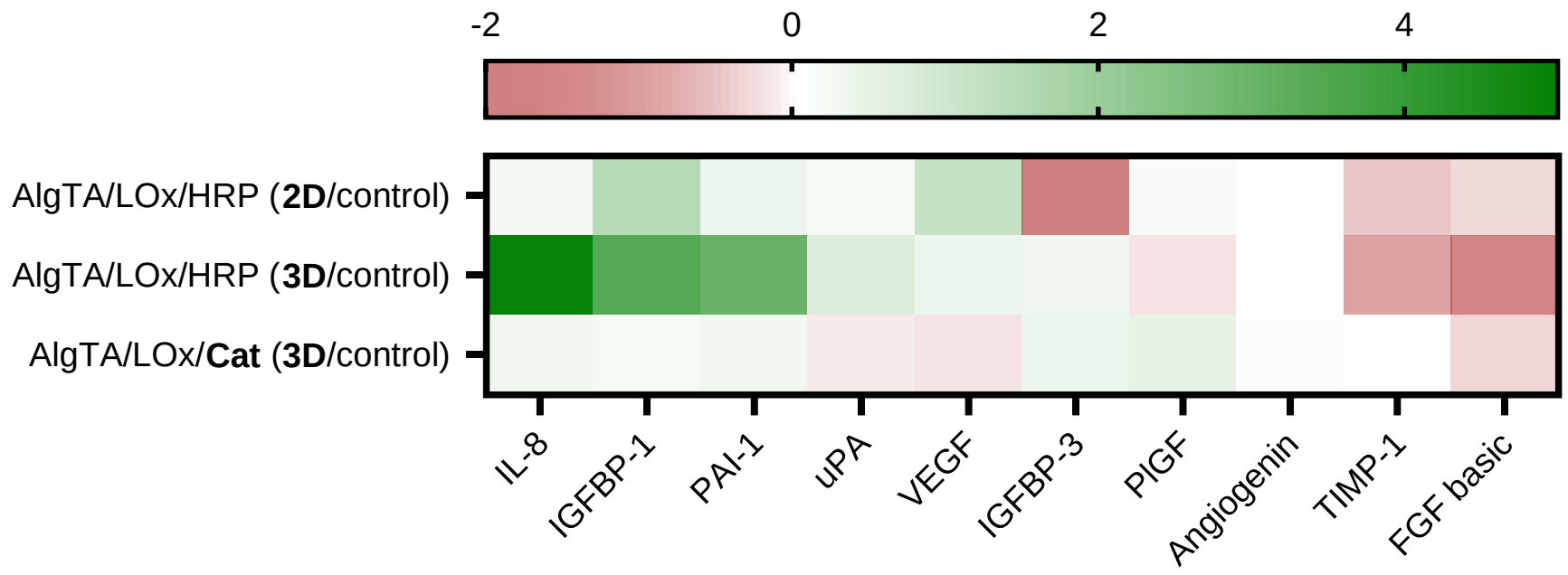

Supplementary Fig. 8 | Relative proteome profiling of selected tumour-associated proteins at day 2 versus matched controls. Replicates: AlgTA/LOx/HRP in 2D and AlgTA/LOx/Cat in 3D, n = 2 biological replicates with two on-membrane replicates; AlgTA/LOx/HRP in 3D, n = 3 biological replicates with two on-membrane replicates. Shades of red indicate decreased relative protein abundance, and shades of green indicate increased relative protein abundance. Data are presented as mean relative abundance.

9. The manuscript would benefit from clearer articulation of the intended applicability of the material system, particularly in light of the pronounced growth arrest and loss of viability observed upon spheroid encapsulation.

Reply

We agree that the pronounced growth arrest and reduced viability upon spheroid encapsulation require clearer functional framing. In the revised manuscript, we explicitly state that the material is not designed as a growth-supporting tissue-engineering scaffold. Instead, it is designed as a metabolite-gated containment matrix that uses elevated local lactate flux to trigger covalent network formation and restrict tumour-cell expansion or invasion.

We have therefore reframed the biological response as follows: early gelation is compatible with cells, but prolonged confinement within the AlgTA/LOx/HRP matrix suppresses spheroid expansion, reduces metabolic activity and increases cell death. This behaviour is aligned with the intended containment function of the material platform.

This revised framing is now included in the Results on **p. 14**, where spheroid expansion and metabolic activity are discussed with reference to **Fig. 4g,h**, and in the integration paragraph on **p. 17**. The Discussion now presents the system as a proof-of-concept bio-synthetic containment strategy for lactate-rich tumour environments and explicitly acknowledges that further in vivo, degradation, immunogenicity, hypoxia and biocompatibility studies are required before therapeutic conclusions can be drawn. These changes are included on **pp. 18–19**.

10, While the chemistry and conceptual framework are innovative, the intended therapeutic purpose of this development remains insufficiently articulated. The work appears novel; however, a clear and compelling application is not convincingly defined. The authors are asked to explicitly clarify their primary objective: Is this system envisioned

as a therapeutic intervention, a containment strategy, a drug delivery platform, or primarily as a proof-of-concept material responsive to disease-associated metabolites? At present, the translational direction and intended clinical use are ambiguous.
If positioned as a therapeutic alternative for cancer, the manuscript should clearly explain the clinical rationale behind this approach. Given that several well-established and increasingly precise therapeutic strategies already exist (e.g., targeted therapies, immunotherapies, antibody–drug conjugates, locoregional treatments), it is not evident what specific unmet need this material addresses or in what context it would provide a superior or complementary benefit.Alternatively, if the authors envision a methodological or diagnostic application, this should also be explicitly justified. Notably, robust and widely validated methodologies already exist for monitoring lactate levels, tumour metabolism, viability, hypoxia, and metastatic potential (e.g., metabolic imaging, biosensors, molecular markers). Therefore, the added value of this system over current technologies needs to be clearly defined.
Overall, the manuscript would be significantly strengthened by a more precise statement of intent, a clearly delineated use-case scenario, and a critical discussion positioning this platform within the existing therapeutic and technological landscape.

Reply
We thank the reviewer for the opportunity to define the translational positioning of this work more clearly. We have revised the manuscript to state that the primary objective is a proof-of-concept, metabolite-gated containment strategy rather than a diagnostic lactate sensor, passive drug-delivery vehicle or stand-alone cancer therapy. The material uses lactate not as a measurement endpoint, but as a biochemical fuel that is converted into local covalent matrix formation. The intended use-case is locoregional confinement of residual tumour cells in lactate-rich environments, with post-surgical glioblastoma resection cavities serving as one motivating example. This framing addresses an unmet need distinct from systemic targeted therapies, immunotherapies or molecular diagnostics: the material does not aim to detect metabolic activity or replace established treatments, but to convert local metabolic activity into a physical barrier that constrains invasion.
To support this translational positioning, we added experiments with patient-derived heterogeneous glioblastoma spheroids. Patient-derived spheroids were generated from three donor specimens, as described in the Methods on **p. 25**. The invasion assay and image-analysis workflow are described on **p. 30**. The study was conducted under institutional ethical approval and informed consent, as stated in the Ethics section on **p. 31**. The corresponding patient-derived glioblastoma data are now shown in **Fig. 5c–e** on **pp. 16–17**. Over 7 days, AlgTA/LOx/HRP treatment reduced the invasive area compared with the AlgTA-free control and promoted spheroid encapsulation. The final Fig. 5 legend reports the invasive-area comparison as **Fig. 5d**, with $P = 0.042$ on day 1, $P = 0.003$ on day 3 and $P < 0.001$ on day 7. A representative photograph of AlgTA/LOx/HRP-encapsulated patient-derived glioblastoma spheroids after 7 days is additionally provided in **Supplementary Fig. 32**.

We have expanded the section "Lactate-gated gelation extends to additional tumour spheroid models" on **p. 15** and the Discussion on **pp. 18–19** to clarify the intended application. The revised Results state:
"To further explore the translational relevance of this concept, we evaluated the system using patient-derived heterogeneous glioblastoma spheroids generated from surgically resected tumour material under institutional ethical approval and informed consent (**Fig. 5c**). Over a culture period of seven days, the material promoted encapsulation of the spheroids and significantly reduced the invasive area compared to the AlgTA-free control (**Fig. 5d,e**; **Supplementary Fig. 32**)."
The revised Discussion states:
"In a potential clinical scenario, such as the resection cavity following surgical removal of malignant glioblastoma tumours, the platform could be developed as a feedback-controlled mechanical containment barrier. Unlike conventional static hydrogels or cavity-filling materials, the system strengthens its network in response to elevated local lactate concentrations associated with highly glycolytic tumour regions. In this context, metabolic activity acts as a trigger for structural adaptation, enabling local matrix formation that may restrict the migration of residual infiltrative cells and therefore motivates evaluation as a locoregional strategy to reduce recurrence risk. We therefore envision this platform as a complementary locoregional strategy that could, in future, be combined with established surgical, chemotherapeutic or immunotherapeutic treatments rather than replacing them."
We also added a limitations statement to avoid overinterpreting the current data. The revised Discussion on **p. 19** now states that translation will require biodegradable or reversibly cleavable linkers, pharmacokinetic and biodistribution studies, evaluation in healthy and tumour-bearing tissue models, mitigation of enzyme immunogenicity, operation under hypoxic conditions, larger donor cohorts, in vivo validation, immune-competent models and orthogonal validation of proteomic markers before therapeutic conclusions can be drawn.

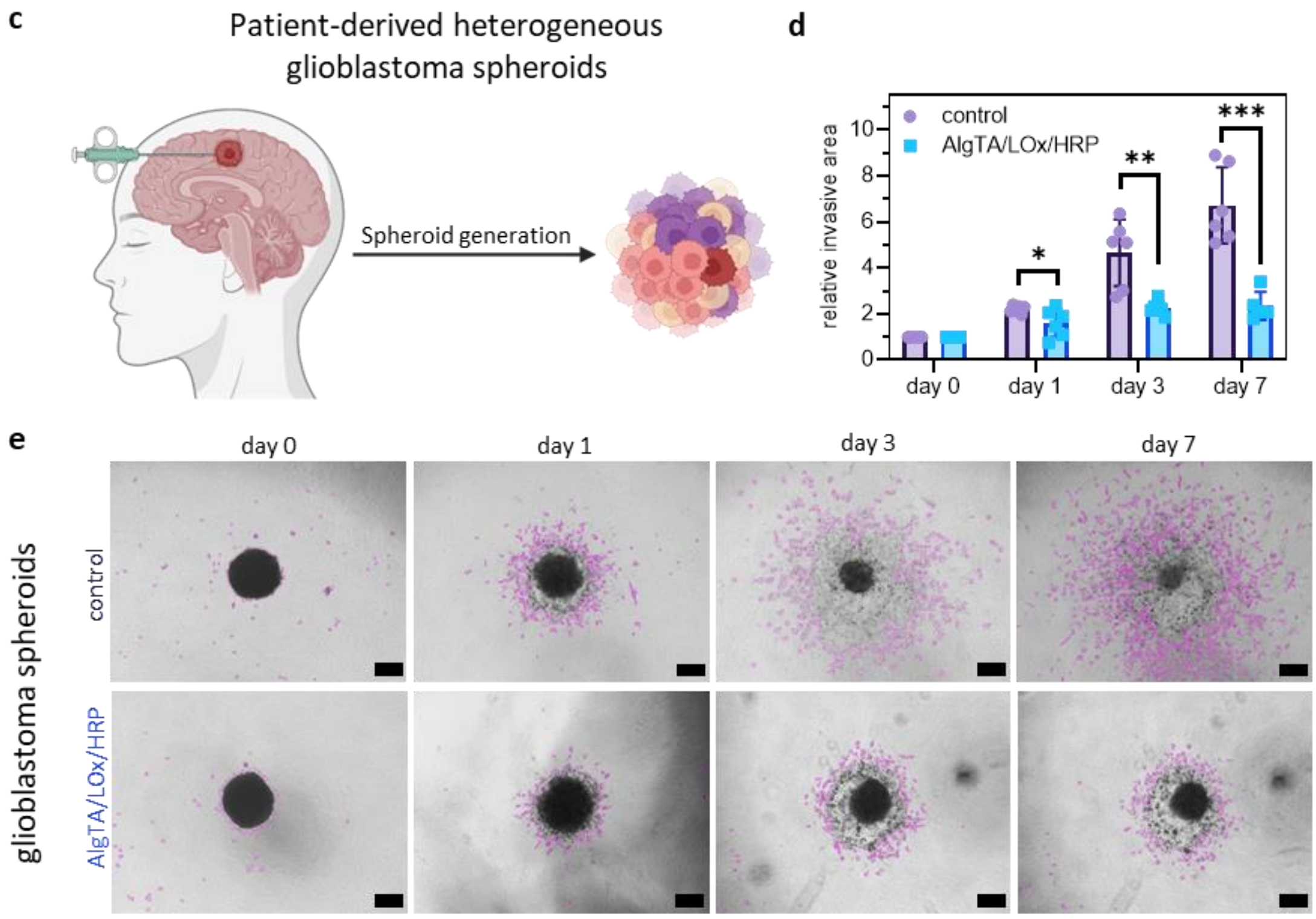

Fig. 5 | Applicability across glycolytic tumour models.

Unless stated otherwise, data are mean ± s.d. (day 2). Statistics: unpaired two-tailed Welch's t-test; $P < 0.05$ (*), $P < 0.01$ (**), $P < 0.001$ (***), $P > 0.05$ (ns, not significant). Formulation (where applicable): 3 wt% AlgTA, 10 U mL^{-1} HRP, 1 U mL^{-1} LOx; control: medium without AlgTA/LOx/HRP.

(c) Formation of patient-derived heterogeneous glioblastoma spheroids. Following surgical tumour resection, glioblastoma specimens are dissociated into single cells, which are subsequently used to generate stable spheroids. Patient-derived material was obtained under institutional ethical approval and informed consent; donor/sample information is provided in Methods.

(d) Invasive area of patient-derived glioblastoma spheroids with and without AlgTA/LOx/HRP after up to 7 days; ≥ 5 spheroids per condition. Data shown are representative of three independent experiments from different donors. Shown *P* values: control vs. AlgTA/LOx/HRP; day 1, $P = 0.042$; day 3, $P = 0.003$; day 7, $P < 0.001$.

(e) Representative brightfield micrographs of patient-derived glioblastoma spheroids after up to 7 days of incubation. Formulations: Control, regular medium without AlgTA/LOx/HRP; AlgTA/LOx/HRP, 3 wt%/1 U mL^{-1}/10 U mL^{-1}. Purple areas indicate the invasive area. Scale bar, 200 µm.

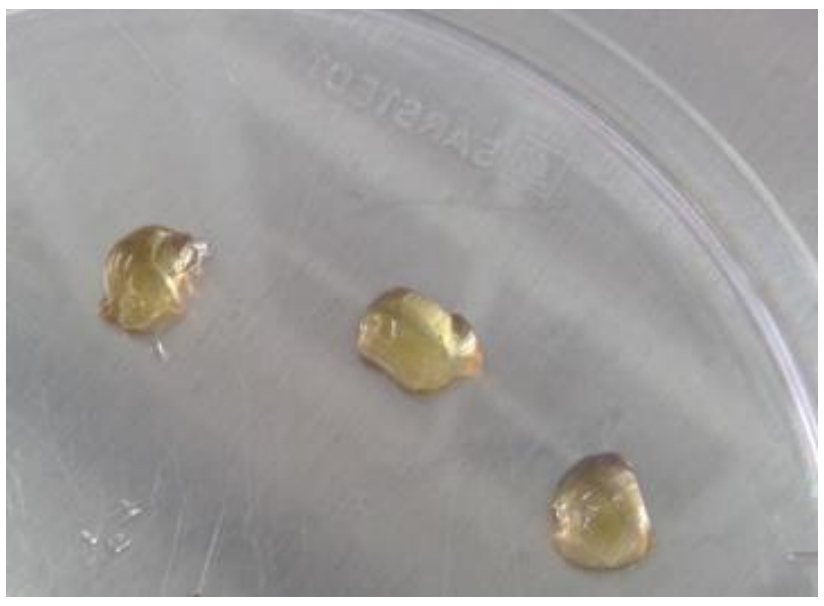

Supplementary Fig. 9 | Representative photograph of AlgTA/LOx/HRP-encapsulated patient-derived glioblastoma spheroids after 7 days of incubation

Reviewer #4 (Remarks to the Author):

The study "Lactate-instructed Hydrogelation Enables Cell-Driven Matrix and Phenotype Reconfiguration" by Braun et al. presents a metabolism-responsive alginate-tyramine (AlgTA) hydrogel system in which enzymatic cascades (LOx/HRP) initiate covalent crosslinking in response to lactate flux. The study aims to link material stiffening to cellular phenotype reconfiguration via a "redox landscape," providing a sophisticated approach to cell-instructed biomaterials.

In the PDF files, I provide details and specific annotations, with side comments, to support the criticism points below.

Major Revisions (as for scientific description and accuracy)

1. Figure 2 Labeling Mismatch

There is a significant discrepancy between the labeling of Figure 2 subplots and the figure caption. Subplots (b), (c), and (d) in the image do not correspond to the descriptions in the text (NMR is b, GPC is c, Schematic is d).#

Reply

We apologize for this oversight. The labels in **Fig. 2** have now been corrected to align with the caption. Specifically, panel b now corresponds to the ^{1}H NMR spectrum, panel c to the SEC/GPC traces, and panel d to the H_2O_2/HRP-mediated oxidative coupling schematic. The corrected figure and caption are included in the revised manuscript on **p. 6**.

2. Sample volume to Rheological Geometry Discrepancy

It is reported a 35 µL sample volume, for an 8 mm parallel-plate geometry, with a 0.5–0.6 mm gap. Geometrically, this volume exceeds the theoretical capacity of the gap (~25–30 µL). Please clarify the method (e.g., if the samples were trimmed, overfilled meniscus, etc.).

Reply

We thank the reviewer for pointing out this discrepancy. The reviewer is correct; the gap used for these measurements was 0.6–0.7 mm rather than the originally stated 0.5–0.6 mm. At a gap of 0.7 mm, the 35 µL sample volume correctly fills the 8 mm parallel-plate geometry and forms a stable meniscus. We have updated the Methods to reflect the accurate gap dimensions.

The revised Methods state:

"The experiments were conducted using an 8 mm parallel-plate geometry, with hydrogel samples of 35 µL volume, resulting in a gap size ranging from 0.6 to 0.7 mm."

This change is included in the revised manuscript on **p. 21**.

3. LVE Region Definition

The text states the Linear Viscoelastic (LVE) region was "identified at" 1% strain. As the LVE is a range, not a discrete point, the authors should instead define the range (Fig. S7) and state that 1% was selected as a strain amplitude within that range.

Reply

We agree with the reviewer's observation that the linear viscoelastic region is a range rather than a discrete point. We have revised the manuscript to state that the linear viscoelastic region extended to at least 10% strain at 1 Hz. A strain amplitude of 1% was therefore selected for subsequent time-sweep and frequency-sweep measurements because it lies safely within the linear viscoelastic region.

This clarification is included in the Results on **p. 7** and in the Methods on **p. 21**. The revised Methods state:

"The linear viscoelastic region extended to at least 10% strain at 1 Hz. Oscillatory time sweeps were therefore performed at 1% strain and 1 Hz at 25 °C."

The corresponding rheological amplitude- and frequency-sweep data are shown in **Supplementary Figs. 9 and 10** on **pp. S6–S7**.

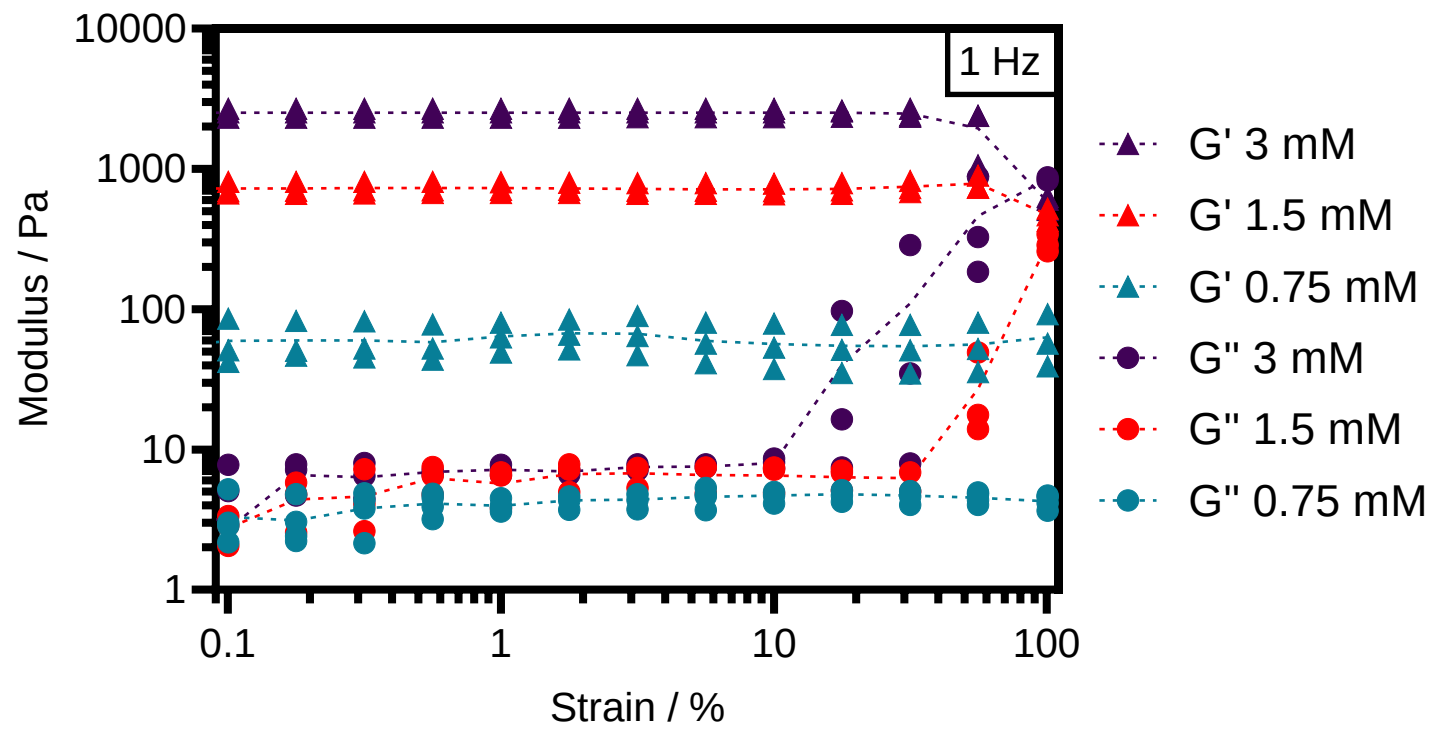

Supplementary Fig. 10 | Amplitude-sweep rheology of AlgTA in culture medium. Formulations: 3 wt% AlgTA, 10 U mL^{-1} HRP, 0.75, 1.5, or 3 mM H_2O_2. Connecting lines act as visual aids; points show individual replicates; data are mean with individual values shown (n = 3 independent gel formulations).

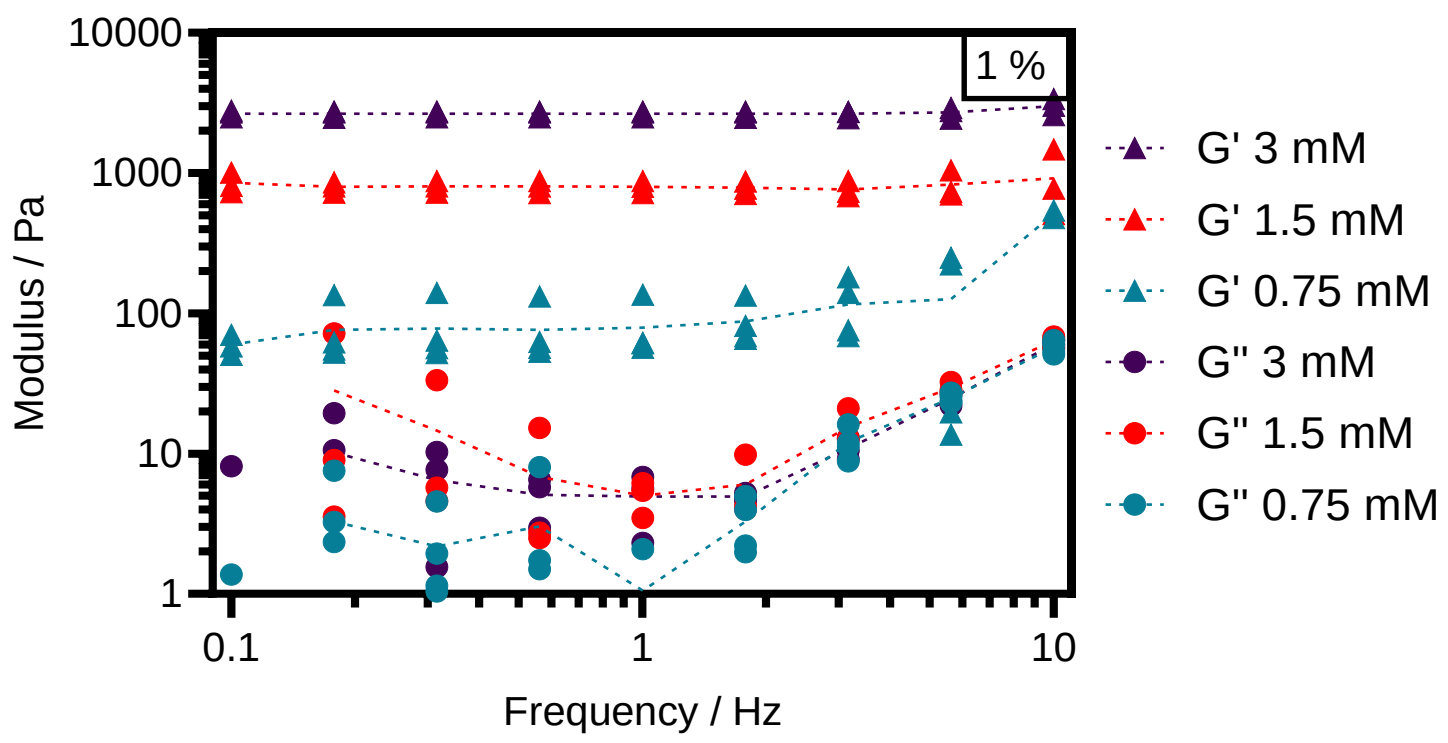

Supplementary Fig. 11 | Frequency-sweep rheology of AlgTA in culture medium. Formulations: 3 wt% AlgTA, 10 U mL^{-1} HRP, 0.75, 1.5, or 3 mM H_2O_2. Connecting lines act as visual aids; points show individual replicates; data are mean with individual values shown (n = 3 independent gel formulations).

4. Kinetics of Gelation (Fig. S10)

Is the data here once-through, or does it stand for n=3? In fact, the authors should recognize that they start already with a gel. Following an internal metastable transformation (induction period) of approximately 5–10 minutes, the material develops a significant secondary structure (increase in the storage modulus). This kinetic profile deserves a brief discussion (and possibly reference to prior work on physical vs. covalent gels) to clarify the observed change in the network's nature.

__Reply__

We thank the reviewer for this helpful comment. We have updated the lactate-triggered rheological data to show three independent gel formulations. These data are now provided as **Supplementary Fig. 13** on **p. S8**.

We have also revised the interpretation of the early rheological response. We agree that the initial material displays a viscoelastic response before the sharp increase in storage modulus. We now distinguish this initial physically entangled AlgTA solution (**Fig. R4**) from the subsequent chemically crosslinked network. The induction period reflects the time required for LOx-mediated H_2O_2 generation and HRP-mediated phenol coupling to establish a sufficient density of covalent junctions. The sharp increase in G′ therefore marks the transition from a physically entangled viscoelastic solution to a covalently crosslinked network.

This clarification has been added to the Results on **p. 8:**

"The low initial modulus before the rise in G′ is consistent with a physically entangled AlgTA solution, whereas the subsequent increase in G′ reflects accumulation of covalent phenol junctions." The corresponding measurement conditions are also described in the Methods on **p. 21**.

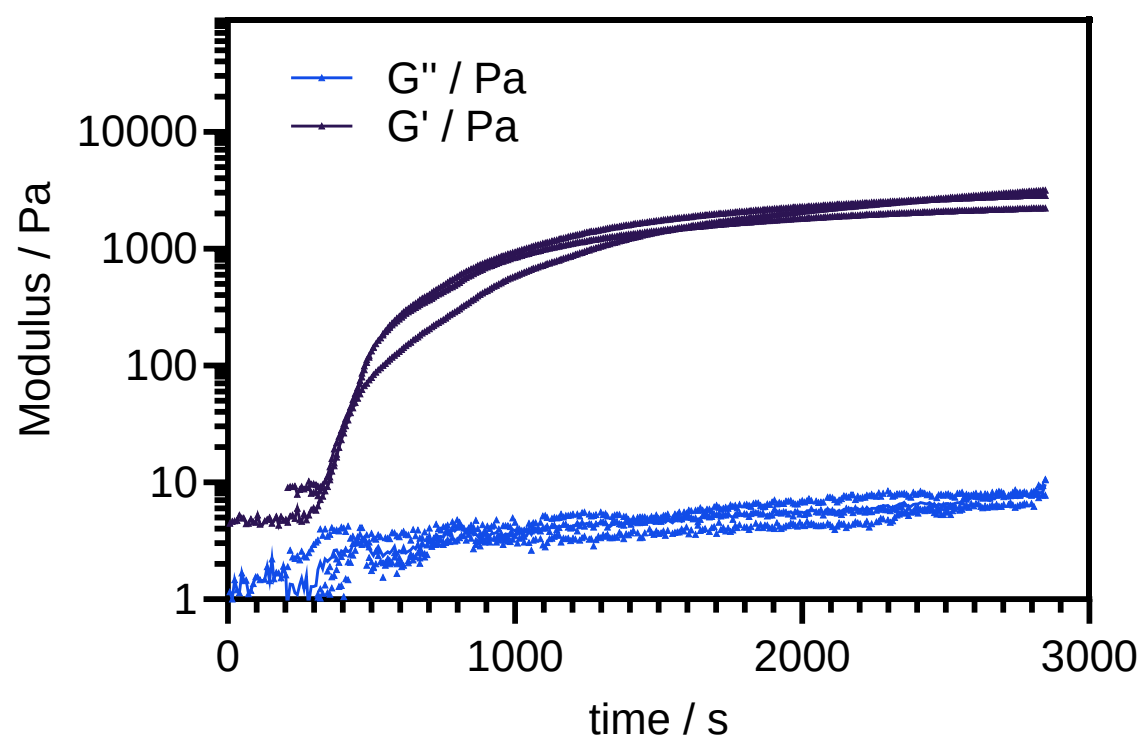

Supplementary Fig. 12 | Lactate-triggered gelation of AlgTA. Oscillatory time-sweep rheology of 3 wt% AlgTA in PBS after addition of 10 mM sodium lactate to activate the lactate oxidase–horseradish peroxidase cascade. Formulation: 3 wt% AlgTA, 10 U mL^{-1} HRP and 1 U mL^{-1} LOx. Measurements were performed at 1 Hz and 1% strain. Points show individual replicates; data are mean ± s.d. (n = 3 independent gel formulations).

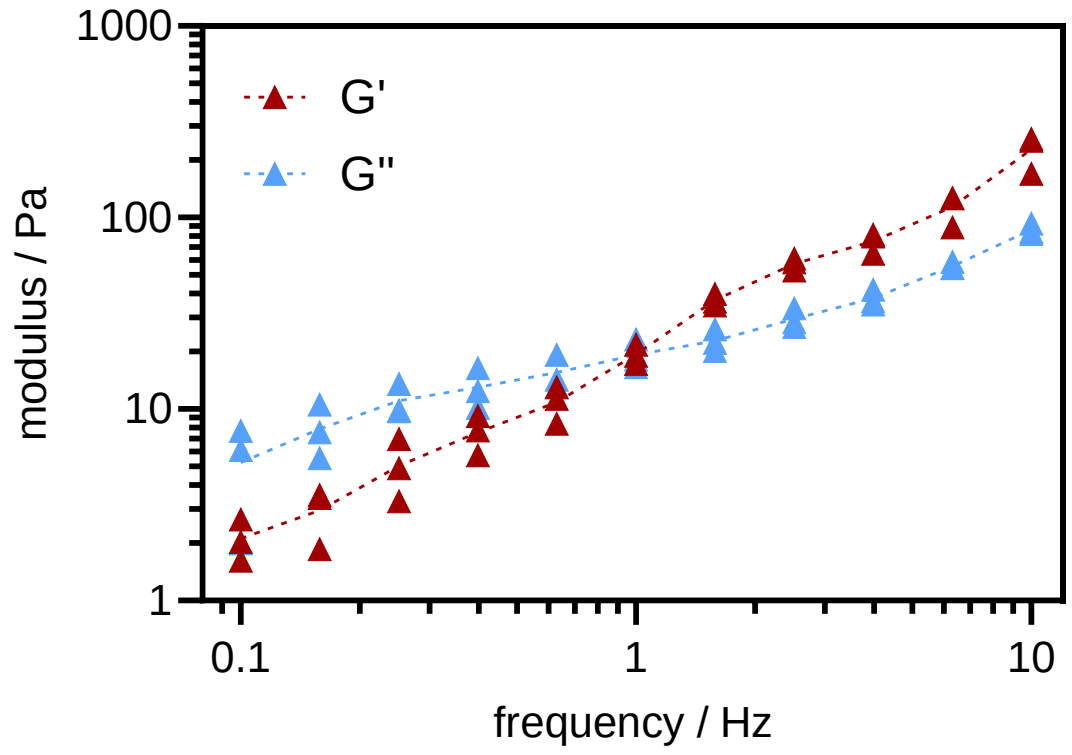

Fig. R4 Frequency-dependent viscoelastic response of alginate. Frequency-sweep rheology of 3 wt% low-viscosity alginate in PBS measured at 1% strain. Points show individual replicates; data are mean ± s.d. (n = 3).

5. Structural Gap and Ambiguous Notation (SI)

Supplementary Fig. 1 is never cited in the main text and should be linked to the relevant section. Furthermore, the integrals used for DS and G/M calculations (IA, IB, IC) are labeled and depicted inappropriately.

Reply

We appreciate the reviewer's comment. **Supplementary Fig. 1** is now explicitly cited in the main text within the section "Phenol-functionalised alginate supports peroxide-triggered covalent hydrogelation". We also revised the corresponding sentence to make the connection between the NMR spectra, G/M-ratio determination and degree-of-substitution calculation clearer. This change is included in the main manuscript on **p. 5**.

The revised manuscript states:

"Aromatic resonances at 7.10–7.60 ppm integrated relative to the glucuronic-acid anomeric signal at 5.35 ppm yielded a DS of ~2.5 mol% tyramine per uronate unit (Supplementary Figs. 1 and 2, Supplementary Equations 1 and 2; further discussion of alginate characterisation and functionalisation is provided in the Supporting Information)."

The revised Supporting Information now presents the alginate M/G-ratio determination and degree-of-substitution calculation in **Supplementary Equations 1 and 2**, with the associated NMR spectra shown in **Supplementary Figs. 1 and 2** on **pp. S2–S3**.

Supplementary Equation 1

$$\frac{M}{G} = \frac{I_B + I_C - I_A}{I_A} = \frac{4.67 + 1{,}00 - 1{,}82}{1.82} = 1.84$$

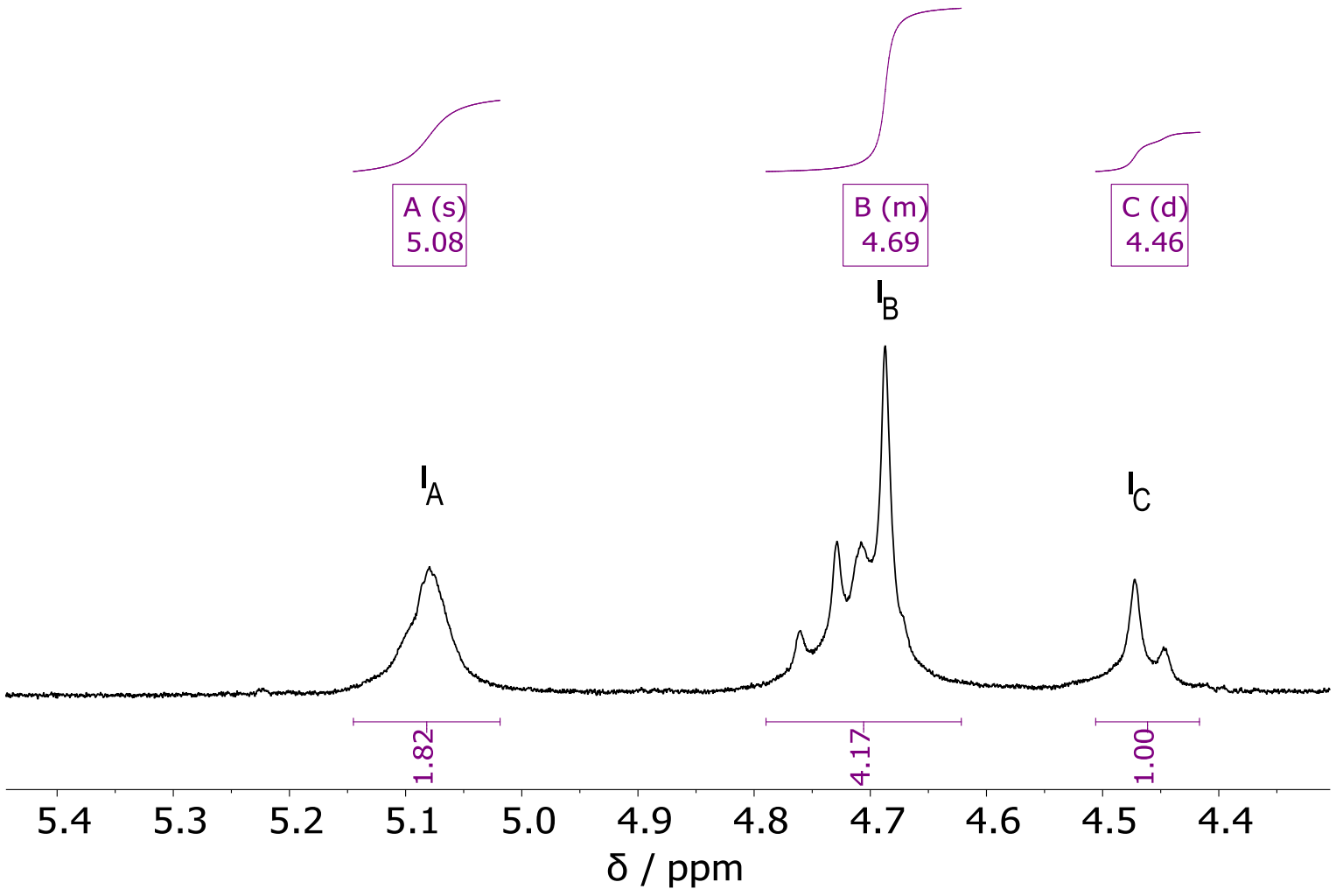

Supplementary Fig. 13 | ^{1}H NMR spectrum of sodium alginate in the spectral region from 4.3 to 5.4 ppm (700 MHz, D_2O, 363 K).

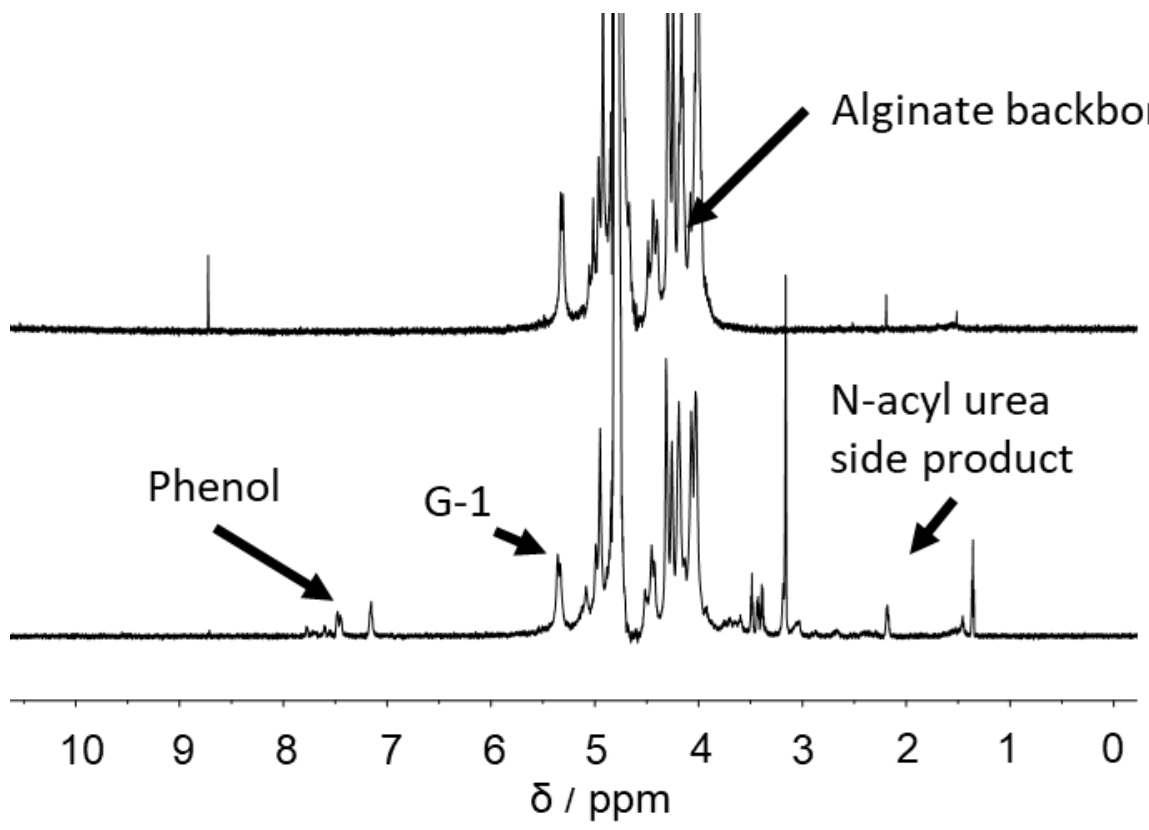

Supplementary Fig. 14 | ^{1}H NMR spectra of AlgTA and sodium alginate (0–10 ppm, 700 MHz, D_2O, 323 K).

6. Concluding discussion on Redox Landscape (Lines 376-378)

While the conceptual link between "metabolic flux" and "phenotype reconfiguration" is a strong point of the paper, the current narrative relies on shifting between different terms (signaling, landscape, flux) without a concrete bridge. A brief, focused discussion of the relationship between the rate of material gelation and specific biological signaling events should be provided.

Reply

We thank the reviewer for this important conceptual comment. We have substantially revised the manuscript to provide a clearer mechanistic bridge between lactate flux, H_2O_2 generation, covalent gelation and cellular response.

In the revised interpretation, lactate-derived H_2O_2 has two distinct roles depending on the experimental geometry. In the transwell setup, the AlgTA/LOx/HRP material is physically

separated from the cell layer. H_2O_2 generated during lactate oxidation can therefore diffuse and contribute to a redox-associated stress/adaptation response, which is reflected by altered relative abundance of IGFBP-1, IGFBP-3 and VEGF in the proteome-array analysis. In the 3D spheroid setup, by contrast, H_2O_2 is generated close to the spheroid surface and is consumed locally by HRP-mediated phenol coupling. The dominant feedback therefore shifts from soluble redox signalling toward pericellular network formation, transport restriction and mechanical confinement. This distinction now explains why the 2D and 3D proteomic responses differ and why VEGF is not further induced in encapsulated spheroids.

We revised the Results accordingly in the section "Local gelation redirects proteomic and morphological responses" on **p. 12**. We also revised the Discussion on **p. 18**, which now states: "The data also reveal compartment-dependent signalling. In the transwell geometry, diffusible H_2O_2 generated by the AlgTA/LOx/HRP cascade produces a redox-associated stress and adaptation signature. In spheroids, by contrast, locally generated H_2O_2 is consumed during phenol coupling near the spheroid surface, shifting the dominant feedback from soluble redox signalling toward pericellular network formation, transport restriction and mechanical confinement."

This revision avoids using "redox landscape" as an undefined conceptual term and instead describes the experimentally supported sequence of lactate flux, peroxide generation and consumption, gelation, transport restriction and phenotype remodelling.

Minor Revisions

I. Citation issue

While Ref 13 is mentioned in the discussion, it should be cited specifically in the description of the AlgTA (i.e., the established protocol of Schulz et al.).

<u>**Reply**</u>

We thank the reviewer for this correction. We have updated the manuscript to ensure that Schulz et al. is cited directly in the description of the AlgTA synthesis protocol. This change is included in the revised manuscript on **p. 4**.

<u>The revised manuscript states:</u>

"Phenol-functionalised alginate (AlgTA) was synthesised by carbodiimide-mediated conjugation of tyramine to sodium alginate using EDC/NHS chemistry, modified from Schulz et al."

The corresponding **Fig. 2** legend was also updated on **p. 6**.

II. Data Presentation in SI

The rheological plots in the Supporting Information are currently too small, and the y-axes (almost two orders of magnitude increase in G' axis to accommodate legends).

<u>**Reply**</u>

We thank the reviewer for the feedback regarding data presentation. We have revised the rheological plots in the Supporting Information to improve readability. Specifically, the rheological data in **Supplementary Figs. 8** (see below) **& 9–10** (see above) were reorganised and enlarged, and the captions were revised to define formulations, replicate numbers and data presentation more clearly. These changes are included in the Supporting Information on **pp. S6–S7**.

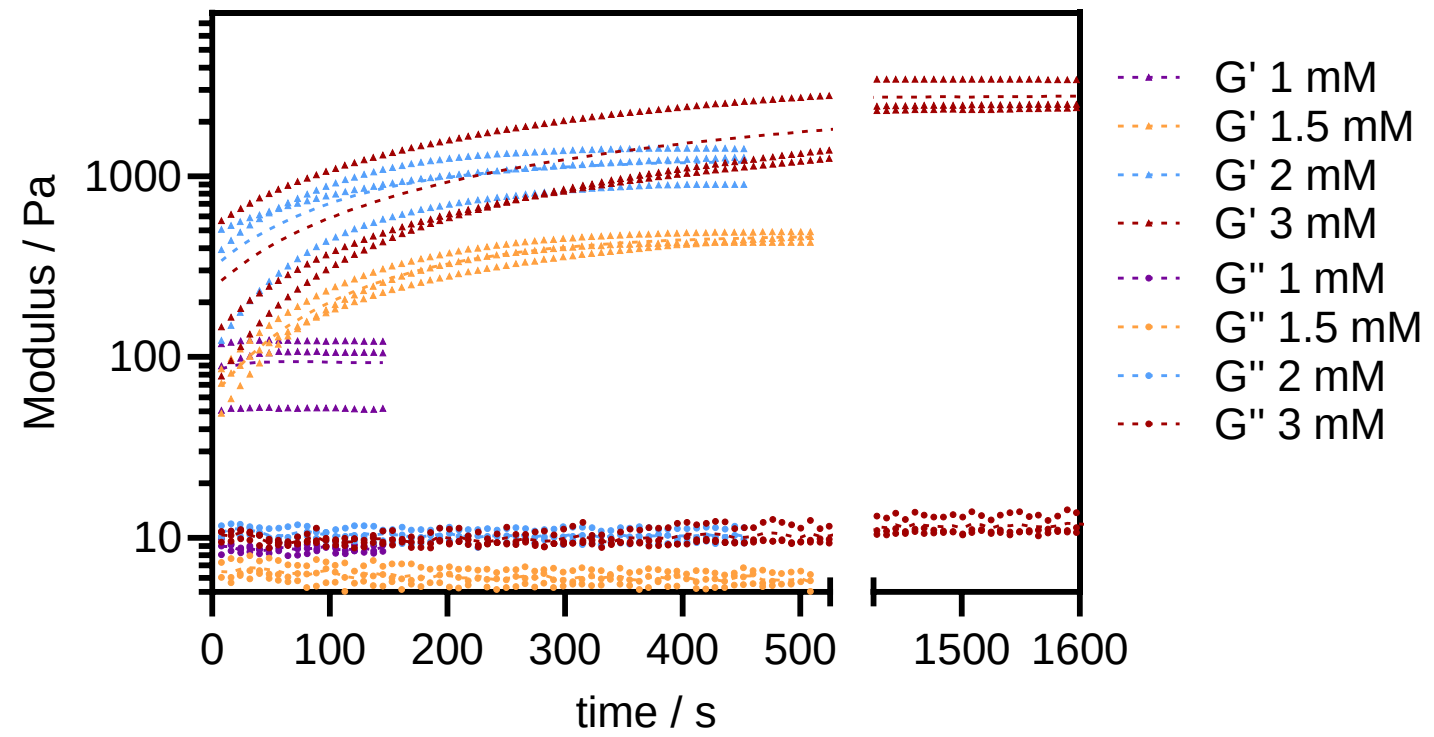

Supplementary Fig. 15 | Peroxide-triggered gelation of AlgTA in culture medium. Oscillatory time-sweep rheology of 3 wt% AlgTA in culture medium, 10 U mL-1 HRP and 1, 1.5, 2, or 3 mM H2O2. Connecting lines act as visual aids; points show individual replicates; data are mean with individual values shown (n = 3 independent gel formulations).

III. Time evolution of storage modulus (Fig. S9)

The 12-day stability study is presented as a single bar chart. Given the duration of the experiment, a time-course plot would be more appropriate to show the kinetics of the G'/G0' ratio, e.g., modulus vs. 2-day (or 3-day) plot.

Reply

We agree that a time-course plot better illustrates the stability of the network over time. We replaced the previous bar-chart presentation with a time-course plot showing the normalised storage modulus over 1, 3, 7 and 12 days. These data are now shown in **Supplementary Fig. 11** on **p. S7**. We also added representative macroscopic photographs of the hydrogels after incubation in cell lysate or buffer control for 1, 3, 7 and 12 days, now shown in **Supplementary Fig. 12** on **p. S8**.

The revised **Supplementary Fig. 11&12** caption states that hydrogels were incubated with A549 cell lysate or buffer control at 37 °C for 1, 3, 7 and 12 days, that gel integrity was quantified by oscillatory rheology and normalised to the corresponding PBS buffer control, and that all comparisons were not significant. The corrected P values are day 3, P = 0.771; day 7, P = 0.807; and day 12, P = 0.964.

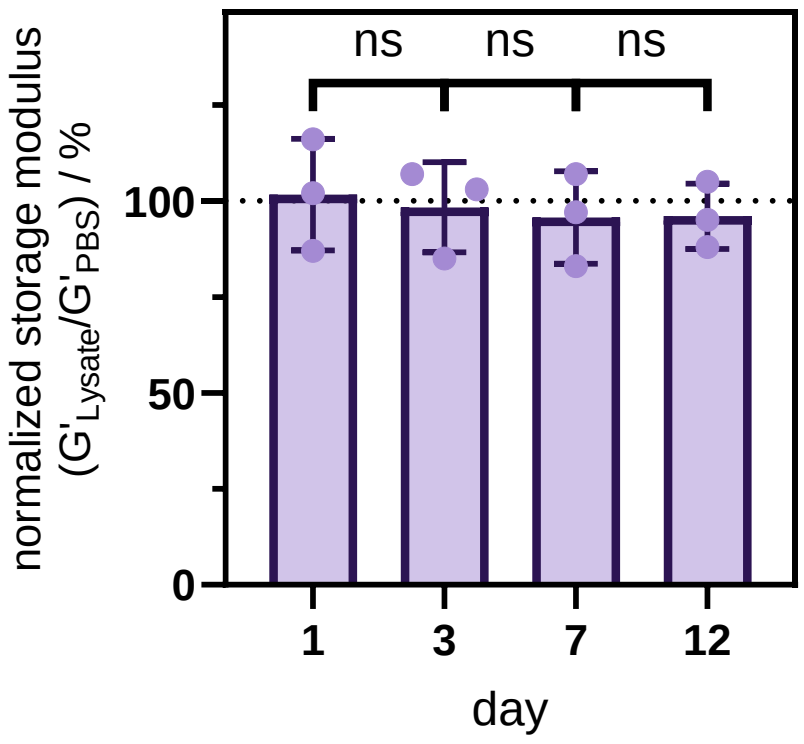

Supplementary Fig. 16 | Stability of hydrogels in the presence of cell lysate. Hydrogels were incubated with A549 cell lysate or buffer control at 37 °C for 1, 3, 7 and 12 days. Gel integrity was quantified by oscillatory rheology and normalised to the corresponding PBS buffer control. Data are mean ± s.d. (n = 3 independent gel formulations); statistics: unpaired two-tailed Welch's t-test. Shown P values: day 3, $P = 0.771$; day 7, $P = 0.807$; day 12, $P = 0.964$. All comparisons were not significant (ns, $P > 0.05$).

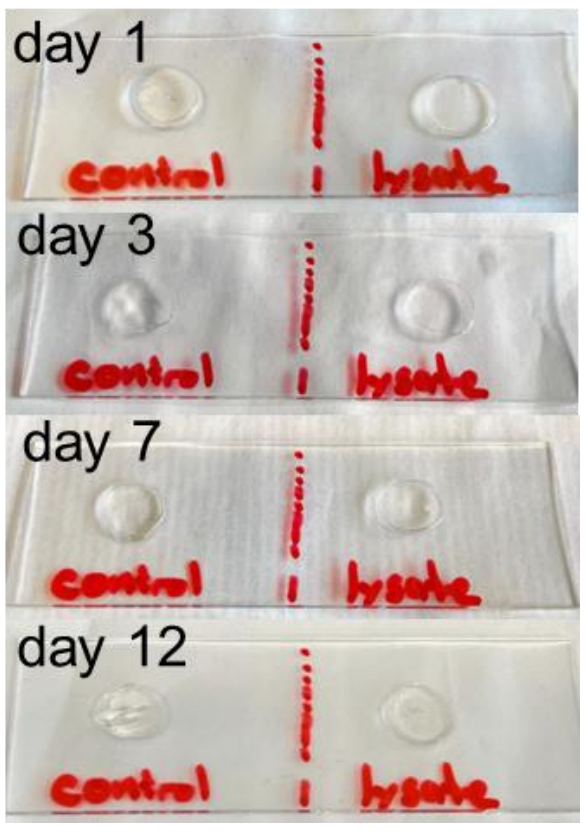

Supplementary Fig. 17 | Macroscopic stability of hydrogels in the presence of cell lysate. Hydrogels were incubated with A549 cell lysate or a buffer control at 37°C. Gels were removed from the incubation chambers and placed on glass slides for imaging after 1, 3, 7, and 12 days of incubation.

IV. Terminology Suggestion

The authors describe the system as "fully synthetic." Given the use of sodium alginate (a natural polysaccharide) and enzymes (HRP/LOx), "bio-hybrid" or "bio-derived" might be more accurate.

<u>Reply</u>

We agree that "fully synthetic" is inaccurate given the natural origin of sodium alginate and the use of enzymes, HRP and LOx. We have revised the terminology throughout the manuscript and now describe the platform as "bio-synthetic." This term reflects the combination of a bio-

derived polysaccharide backbone, enzymatic catalysis and synthetically programmed covalent crosslinking.
This terminology is now used in the Abstract on **p. 1**, the Introduction on **p. 3**, the Results and integration paragraph on **p. 17**, and the Discussion on **pp. 18–20**.
